# Retrieving biodiversity data from multiple sources: making secondary data standardised and accessible

**DOI:** 10.3897/BDJ.12.e133775

**Published:** 2024-09-20

**Authors:** Nubia Marques, Carla Danielle de Melo Soares, Daniel de Melo Casali, Erick Cristofore Guimarães, Fernanda Guimarães Fava, João Marcelo da Silva Abreu, Ligiane Martins Moras, Letícia Gomes da Silva, Raphael Matias, Rafael Leandro de Assis, Rafael Fraga, Sara Miranda Almeida, Vanessa Guimarães Lopes, Verônica Oliveira, Rafaela Missagia, Eduardo Costa Carvalho, Nikolas Jorge Carneiro, Ronnie Alves, Pedro Souza-Filho, Guilherme Oliveira, Margarida Miranda, Valéria da Cunha Tavares

**Affiliations:** 1 Vale Institute of Technology, Belém, Brazil Vale Institute of Technology Belém Brazil; 2 Universidade Estadual do Maranhão, São Luís, Brazil Universidade Estadual do Maranhão São Luís Brazil; 3 Federal University of Jataí, Jataí, Brazil Federal University of Jataí Jataí Brazil; 4 Instituto de Geociências, Universidade Federal do Pará, Pará, Brazil Instituto de Geociências, Universidade Federal do Pará Pará Brazil; 5 Museu Paraense Emílio Goeldi, MPEG, Pós-graduação em Biodiversidade e Evolução, Belém, Brazil Museu Paraense Emílio Goeldi, MPEG, Pós-graduação em Biodiversidade e Evolução Belém Brazil; 6 Pós-Graduação em Zoologia & Laboratório de Mamíferos, Departamento de Sistemática e Ecologia, Universidade Federal da Paraíba, João Pessoa, Brazil Pós-Graduação em Zoologia & Laboratório de Mamíferos, Departamento de Sistemática e Ecologia, Universidade Federal da Paraíba João Pessoa Brazil

**Keywords:** Darwin Core standard, FAIR data, Golfão Maranhense, secondary data

## Abstract

Biodiversity data, particularly species occurrence and abundance, are indispensable for testing empirical hypothesis in natural sciences. However, datasets built for research programmes do not often meet FAIR (findable, accessible, interoperable and reusable) principles, which raises questions about data quality, accuracy and availability. The 21^st^ century has markedly been a new era for data science and analytics and every effort to aggregate, standardise, filter and share biodiversity data from multiple sources have become increasingly necessary. In this study, we propose a framework for refining and conforming secondary biodiversity data to FAIR standards to make them available for use such as macroecological modelling and other studies. We relied on a Darwin Core base model to standardise and further facilitate the curation and validation of data related including the occurrence and abundance of multiple taxa of a region that encompasses estuarine ecosystems in an ecotonal area bordering the easternmost Amazonia. We further discuss the significance of feeding standardised public data repositories to advance scientific progress and highlight their role in contributing to the biodiversity management and conservation.

## Introduction

High-quality, openly available biodiversity datasets (e.g. species occurrence, abundance, traits) are indispensable for the monitoring of species and ecosystems and to improve the development of conservation and management policies (Wetzel et al. 2015, Wetzel et al. 2018). Biodiversity data under FAIR (findable, accessible, interoperable and reusable) principles also help optimising editorial processes for academic publications accelerating peer review, increasing the visibility of scientific papers and improving citation rates (Costello et al. 2013, Piwowar and Vision 2013). Efforts to build and maintain repositories of FAIR data principles have been undertaken by biodiversity data collectors and curators (Hackett et al. 2019), who strive to organise, standardise and share data from a variety of primary (e.g. fieldwork) and secondary (e.g. literature) sources, for example, global initiatives, such as the "Global Biodiversity Information Facility - GBIF" (https://www.gbif.org/), which provide access to comprehensive biodiversity datasets and facilitate their reuse. This is particularly important in the modern era of big data science, which challenges our ability to organise, filter and analyse large and complex datasets (Cao 2016).

The availability of biodiversity data is influenced by several factors, including geographic region, scientific interest and resource availability (e.g. financial and infrastructure constraints), which can affect the quality and type of data produced (Amano et al. 2016). In addition, biodiversity data are not always publicly available, requiring users to conduct extensive searches in scientific publications, technical reports or by directly contacting the researcher who collected the data (Costello et al. 2013). Therefore, conducting systematic literature reviews is often necessary to compile comprehensive databases focusing on biodiversity research programmes. This method is recommended as it provides a rigorous way to search for relevant literature, allowing for peer replication and ensuring data validity and reliability (Xiao and Watson 2019). However, the retrieved data may be in inconsistent and sometimes confusing formats, requiring additional effort from users, including finding separate metadata files, reorganising and renaming fields, integrating aggregated data and discarding poorly documented or questionable data (Parr et al. 2012, Hunt et al. 2015, Culley 2017). The fundamental goal of the structure and standardisation of biodiversity data is to enable them to be understood and used by anyone and to be continuously updated and integrated with other datasets (Borregaard and Hart 2016). There are several guidelines and standards for biodiversity data, including the Darwin Core (DwC; Wieczorek et al. (2012); Darwin Core Maintenance Group (2014) and the DMPTool (https://dmptool.org/).

Finally, data must be shared and archived to ensure findability, accessibility and reusability. Data sharing can occur in a variety of ways, ranging from private sharing on request to depositing data on a public platform. Often, authors make their data available as supplementary material in scientific publications and post datasets on public websites. Sharing biodiversity data is essential for ecological research, conservation and management, education and policy decision-making (Ganzevoort et al. 2017). In addition, ecologists often use shared data for comparative studies, syntheses (e.g. meta-analyses), model parameterisation and reproducibility testing (Michener 2015). Data archiving is a critical final step that allows data to be reused for further analyses and syntheses to address new questions. Whitlock (2011) outlines optimal procedures for archiving ecological and evolutionary data, including selecting the appropriate repository and ensuring the accuracy of data and metadata. Good archiving practices facilitate long-term preservation of data, making them accessible for future research and applications.

Research programmes from megadiverse regions, such as the Neotropics, face difficulties in retrieving, organising and providing quality data due to their intrinsic complexity, which includes unrecognised species and unresolved taxa complexes and taxonomy. To address these challenges and to improve the reusability of secondary biodiversity data, our study had three main objectives:

1. to create a scheme or "pipeline" to improve the usability of secondary data by locating the data, performing quality control, standardising the data and archiving and sharing it;
2. to test our pipeline through a case study, demonstrating the step-by-step management of secondary data according to the FAIR principles; and
3. to evaluate if and how our approach can improve our understanding of the dynamics of regional biodiversity distribution and conservation, promote new scientific studies and knowledge and enhance our ability to generate hypotheses.

Retrieving biodiversity data is not an easy task, as the use of systematic literature searches alone does not guarantee the quality of the data. Additionally, commonly used pipelines for retrieving, standardising and making secondary data available typically overlook grey literature, despite its potential for biodiversity studies. The proposed pipeline is a combination of data retrieval and data management tools that are typically used separately, such as systematic review and the Darwin Core Standard. Following this Biodiversity Data Retrieval Pipeline ensures that secondary data are cleaned, normalised, shared and archived according to the FAIR Data Principles. Additionally, we discuss the challenges of efficient data retrieval, the potential reuse of secondary data in future studies and its limitations. Finally, we predict that initiatives to collect biodiversity data and make it available for reuse can improve knowledge and advance conservation efforts to protect the species, communities and ecosystems of these regions.

## Material and methods

**Figure 1.**
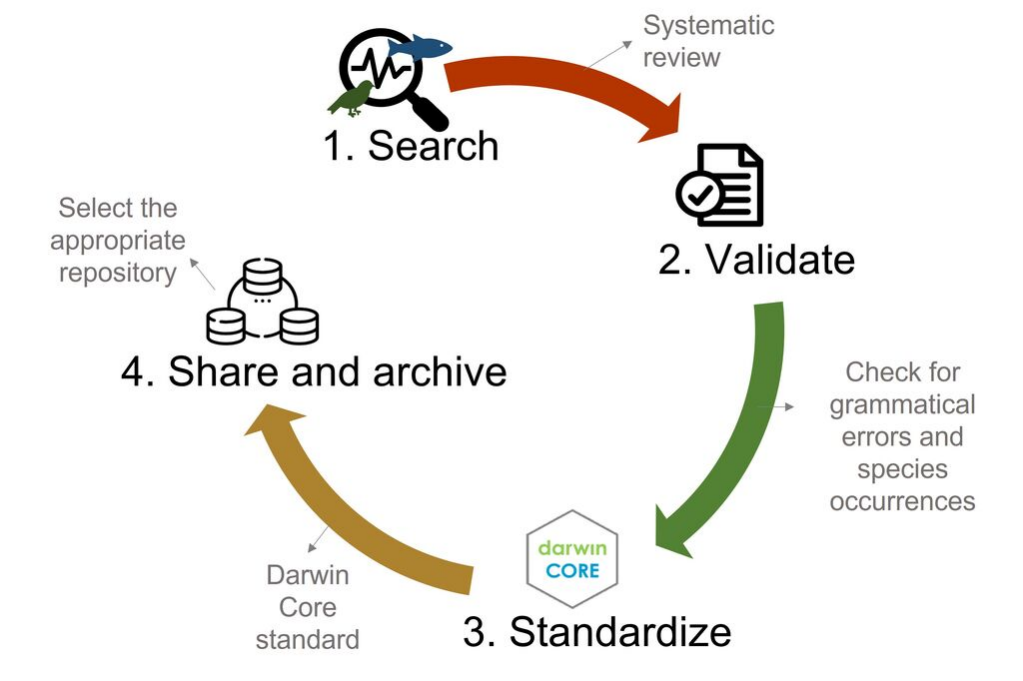
Step-by-step guide for the proposed Biodiversity Data Retrieval Pipeline to retrieve secondary biodiversity data from various sources (e.g. scientific articles, technical reports, theses, dissertations, databases) according to the FAIR principles (findable, accessible, interoperable and reusable).

The Biodiversity Data Retrieval Pipeline was built following four stages (Fig. 1): (1) Search; (2) Validate; (3) Standardise; and (4) Share and archive. All the steps are described below.

To test the pipeline, we selected the *Golfão Maranhense*, a region located in the extreme north of the Amazon (Brazil), due to its richness, ecological diversity and importance as an ecotonal mosaic between the Amazon Forest and the dry ecosystems of eastern South America and because of the scarcity of knowledge about the biodiversity in the region. Although reports on biodiversity in the region exist, they are presented in heterogeneous forms, including scientific articles and non-peer-reviewed technical reports, making it difficult to understand the true distribution of biodiversity richness in the region.

### Study area

**Figure 2.**
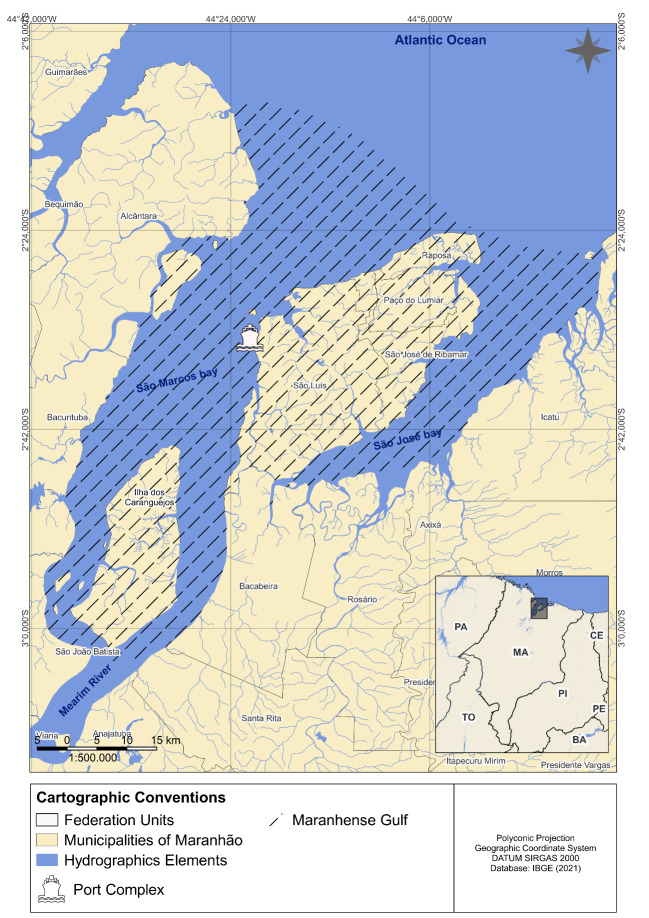
Map showing the *Golfão Maranhense* region, an estuary in the eastern Amazon (Brazil). This is where the secondary biodiversity data was retrieved.

The study was conducted in the *Golfão Maranhense* (Maranhão State, Brazil) including 13 municipalities in the surroundings (Fig. 2). The *Golfão Maranhense* is a vast estuarine complex located in eastern Amazonia (Brazil) and is formed by the *São Marcos* and *São José bays* separated by the island of *São Luís*. This region is an area of high ecological relevance known as the “Macromaré” Mangrove Coast of Amazonia where lies the largest continuous mangrove system in the world, with about 5,414 km of mangroves in north-western Maranhão and 2,177 km in north-eastern Pará (Souza Filho 2005). The climate is tropical humid, with well-defined dry (July to December) and rainy (January to June) seasons and average temperatures around 26^o^C. The area is characterised by semi-diurnal macrotidal with average variations of 4 m and maximum of 7 m, with maximum tidal currents exceeding 4 m/s (Rebelo-Mochel 1997). *São Marcos* and *São José* Bays are port areas that hold significant importance for maritime activities, trade and transportation within the region.

### Step 1: Search- systematic review

The first step in retrieving secondary data is to find the data. To do this, a systematic review of the literature is recommended. We conducted a systematic review performing searches in the platforms Science Direct and Google Scholar and public data repositories such as GBIF, VertNet, Wikiaves and SpeciesLink. During the search on the platforms, we included all the works found, such as scientific articles, books, theses and dissertations. Our search across platforms covered both published (i.e. papers and books) and unpublished literature (i.e. theses, dissertations and environmental consultancy reports focused on licensing). The searches were carried out over two months (June and July 2021) in Brazil. To ensure transparency, completeness and consistency, we followed the "Preferred Reporting Items for Systematic Reviews and Meta-Analyses (PRISMA)" guidelines. The PRISMA framework, with its checklist and flow diagram, facilitates reader comprehension and allows for the assessment of the reliability and validity of the findings.

We followed four steps:

1. Biotic group selection - We have chosen eight biotic groups that represent a substantial proportion of the terrestrial and aquatic biodiversity: mammals (Mammalia), reptiles including turtles, lizards and snakes (Testudines, Squamata), birds (Aves), amphibians (Amphibia), plants (Magnoliophyta), fishes, phytoplankton and benthos.
2. Keywords - keywords were defined considering each biotic group (Suppl. material 1). The keywords must include the name of the biotic group (e.g. Amphibians) and the study area (e.g. Maranhão) and may vary according to the needs of each biotic group.
3. Inclusion criteria - Our inclusion criteria were twofold: (a) studies that were conducted in *Golfão Maranhense* and; (b) studies that included both the geographic coordinates and the finest possible taxonomic level.
4. Data selection - We selected 76 variables to be extracted from the selected studies. These variables were classified into three main categories: (a) General Information - data about the published work, such as title, year, keywords and objectives of the study; (b) Sampling Events - information about when and where the sampling of target taxa occurred, such as date, sampling method, location and geographic coordinates; (c) Occurrences - description of the collected individual, such as epithet, life stage and conservation status. The species' conservation status was sourced from the International Union for Conservation of Nature (IUCN) and the Brazilian Ministry of the Environment (MMA) and applies at the species level rather than the individual level. All variables are described in Suppl. material 2.

### Step 2: Validate- data quality and control

To ensure that the data have the lowest possible error rate, they need to go through a validation process. This process reduces the chances that the final data will contain grammatical errors, which can make it difficult to understand and species that have been incorrectly identified.

We conducted a manual validation process for the *Golfão Maranhanse* data that we optimised in two steps:

1. Identifying and fixing errors - We conducted a thorough examination of the data to identify and correct any errors or inaccuracies.

These errors included:

(a) Scientific names (e.g. “*Boanaranipcs*” [wrong] vs. “*Boanaraniceps*” [correct]);

(b) Geographic coordinates (e.g. “44,321605 [typo without negative sign]” vs. “-44,321605 [correct]”);

(c) Date: we standardised the sampling dates to "Start Month" "Start Year" "End Month" and "End Year" (the original data column, verbatim date, contains the day of sampling, if available).

Additionally, our data cleaning process involved removing duplicates and standardising entries to ensure consistency. By meticulously correcting these issues, we ensured the data's integrity and reliability, making it suitable for further analysis and interpretation.

2. Checking the records - Species occurrence data are susceptible to misidentification and taxonomic inconsistencies, making this a challenging and dynamic task. To ensure the reliability and validity of the species occurrence data, we reviewed the relevant literature for known geographic species distributions and compared them with the collected points. Our team of taxa specialists meticulously checked each entry for inconsistencies and up-to-date taxonomy, according to the most recent accepted taxonomy of each group. Any mismatches between known and collected geographic distributions served as a first alert, indicating the need for further investigation. Additionally, we reviewed the literature for changes in synonymy and updated the occurrence records accordingly.

### Step 3: Standardise

To standardise data from the Golfão Maranhense region, we used the Darwin Core standard (DwC) (Wieczorek et al. 2012). In addition, we manually added columns for data that are not covered by the DwC (e.g. conservation status). DwC is one of the most widely used standards for biodiversity data used as a language for sharing biodiversity data that can be understood by human users and interpreted by computational systems. The DwC provides a straightforward, stable standard that simplifies the process of publishing biodiversity data, promoting the sharing, use and reuse of openly accessible biodiversity data (Wieczorek et al. 2012). Additionally, DwC allows users to adapt terms that name the columns for various applications, including the checklists of species in an area (DoNascimiento et al. 2017).

### Step 4: Share and archive

The last step is to choose the right repository to store the data. For species occurrence data, GBIF (GBIF 2024) may be the best option, as it is a specific repository for biodiversity data that guarantees data quality and open access. Other repositories that are popular and should be considered:

- Integrated Digitized Biocollections (iDigBio 2024);

- Dryad (2024);

- Zenodo (2024);

- Open Science Framework (OSF 2024);

### Statistical analysis

To test whether the number of occurrences depended on the number of taxa in each group, a simple linear regression was performed using R software.

## Results

### Step 1: Search- Systematic review

**Figure 3.**
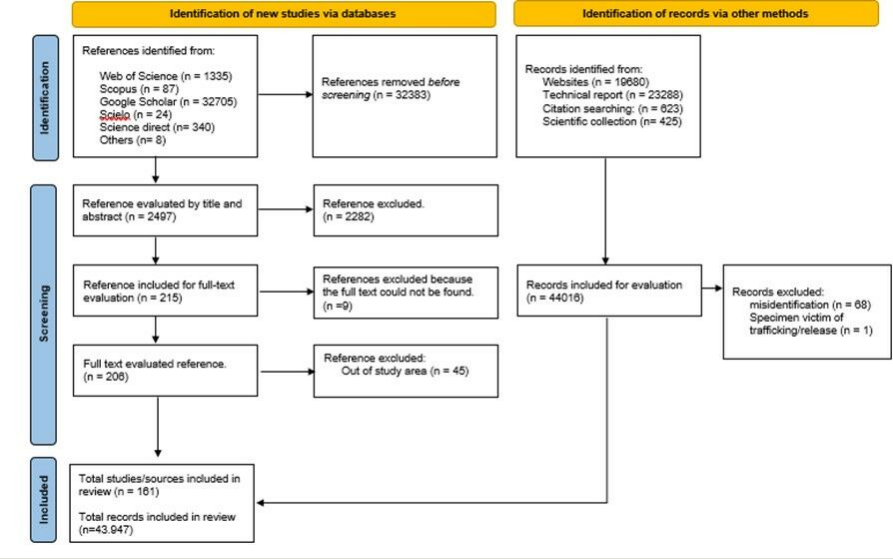
Flowchart of the Preferred Reporting Items for Systematic Reviews and Meta-Analyses (PRISMA) for all groups showing the process of selecting studies throughout systematic review. The selection process includes three stages: (1) identifying the database and choosing the papers; (2) scanning the references and selecting the papers to be included; (3) including the selected papers.

Considering all biotic groups, a total of 161 bibliographical references, including papers and technical reports were included in the systematic review of the literature (Fig. 3). In addition, we included species occurrence records from four public repositories (GBIF, VertNet, Wikiaves, SpeciesLink). Considering only published papers, the group included in the largest number of published papers and reports was plants (n = 59) and the group with the least data sources was benthos (n = 11) (Suppl. material 3 “Preferred Reporting Items for Systematic Reviews and Meta-Analyses – PRISMA”, separated by groups).

### Step 2: Validate- data quality and control

**Figure 4.**
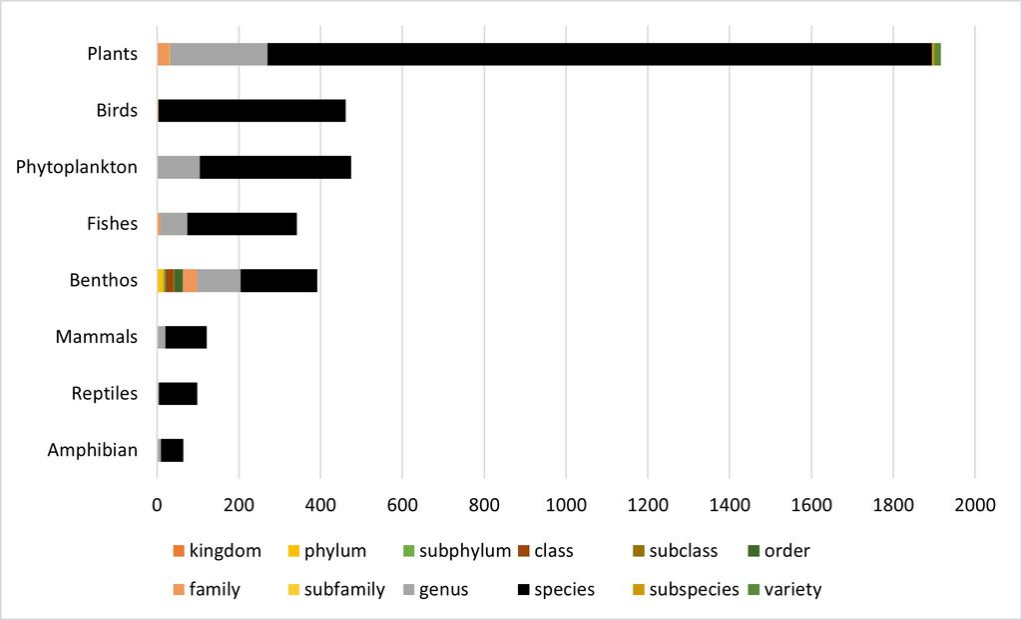
Proportion of each taxonomic level identified for each biological group in the secondary data recovered from the Golfão Maranhense area.

A total of 2,070 occurrence events were obtained from bibliographic references and 43,947 were obtained by public repositories (n = 46,017) from 3,871 taxa. These include birds (Aves, 458 species; three other taxonomic level), amphibians (Amphibia, 55 species; nine to the genus level), reptiles (two Crocodylia; 86 Squamata; 11 Testudines); mammals (Class Mammalia; 101 species; 21 to the genus level), fish (268 species, 74 other taxonomic levels), phytoplankton (370 species; 105 other taxonomic levels), benthos (188 species; 204 other taxonomic levels) and plants (1,624 species; 292 other taxonomic levels) (Suppl. material 4). Most of the taxa were identified to species (81%) and genus (14%) level (Fig. 4). Benthos accounted for the highest number of occurrence events, with 12,510 records and reptiles had the lowest number of occurrence events recorded (570).

Data were carefully analysed by specialists in each group to check for inconsistencies in identification, spelling and, as much as possible, potentiality of identification correctness (e.g. check if the geographic locations were within expected known geographic distribution for each taxon, checking vouchers when possible). A total of 93 occurrence events were deleted, including 92 from taxa that were not correctly identified (76 birds and 16 mammals) and one bird specimen that was a victim of animal trafficking.

### Step 3: Standardise

Of the 76 variables extracted from the studies, 59 were standardised using DwC terms and 17 were adapted due to the lack of appropriate terms for these variables within the current DwC models (Suppl. material 2).

### Step 4: Share and archive

We decided not to publish the database in a specialised database such as GBIF because it contains secondary data that includes information extracted from other databases, including GBIF itself. This would result in duplication of information. We decided to store the data in the Open Science Framework (OSF). OSF is an open access repository that maintains version control, allowing them to track changes to their projects over time. The OSF also assigns Digital Object Identifiers (DOIs), making the data citable and ensuring its long-term preservation. The database is publicly available on the link (Marques 2024). In addition, the database will be accessible through an online platform developed using PowerBI software (Microsoft corporation 2024). This platform will be developed and will be freely available, promoting the dissemination of knowledge related to the biodiversity of the Golfão Maranhense region.

### Statistical analysis

**Figure 5.**
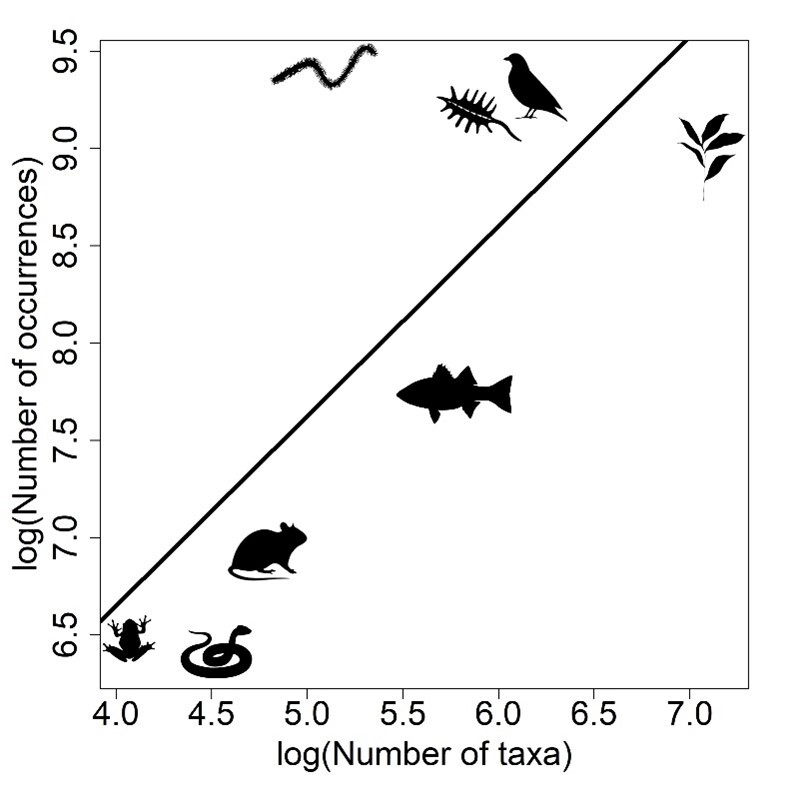
Relationships between numbers of taxa and occurrences gathered through an extensive review of secondary biodiversity data from the Golfão Maranhense area, in the estuarine regions of eastern Amazonia.

The number of occurrences was dependent on the number of taxa in each sampled group (R² = 0.47, p = 0.03). While amphibians and non-bird reptiles were represented by low numbers of both taxa and occurrences, plants, birds and phytoplankton were highly represented for both occurrences and richness. On the other hand, the group “benthos” had a high number of occurrences and a low number of taxa (Fig. 5).

## Discussion

We proposed a workflow to improve our ability to recover higher quality biodiversity data using secondary data sources. We were able to extract a large amount of information about the biodiversity of the *Golfão Maranhense* and transform this unrelated data into organised and re-usable data. This systematic approach ensured data accuracy and reliability, facilitating the potential reuse of information in future studies. A further step that we have begun to take for some groups is the systematic survey of museum collections and analyses, focusing on relevant questions that we have identified along the way (e.g. general patterns of occurrence of migratory birds, sampling biases and gaps for many groups etc.).

Researchers can use existing datasets, such as those obtained through our biodiversity data retrieval method, to conduct a wide range of studies to advance scientific research (Pernat et al. 2024). For example, secondary data can be used to conduct meta-analyses (e.g. Biggs et al. (2020)) for comparative studies across different geographic regions and time, to support ecological modelling of species distributions (e.g. Fletcher et al. (2019)), habitat preferences and potential impacts of environmental change (Bayraktarov et al. 2019) as long as they are used judiciously. However, finding high-quality secondary data can be challenging, as evidenced by a recent survey in which most researchers reported that data finding can be arduous (73%) or difficult (19%) (Gregory et al. 2020). Several initiatives have been launched to collect, standardise, store and make biodiversity data openly available (Costello et al. 2013). For example, international repositories, such as “GBIF” (GBIF 2024) and “Freshwater Biodiversity Data Portal- BioFresh” (http://data.freshwaterbiodiversity.eu/) (for other repositories, see Culina et al. (2018)).

Long-term monitoring datasets can help to understand patterns and changes in ecological variables over time (Costa and Magnusson 2010, Magnusson et al. 2021). This can help identify ecological shifts and potential drivers of biodiversity change (e.g. Marques et al. (2022)). We are currently using our database to increase our knowledge of mammal, bird, fish, amphibian, reptile, marine phytoplankton and benthic species in the *Golfão Maranhense*. We are also studying the patterns and determinants of floristic variation in the region and the temporal variation of migratory birds in the *São Marcos* Bay region. While data collected in standardised monitoring programmes, such as LTER (Vanderbilt et al. 2015), can be directly linked to FAIR's standardised data repositories, other secondary data that are not standardised may be important to rescue, treat and use, as they can be thoroughly revised and curated beforehand and stored with some rule of error estimation to build robust hypotheses to investigate and understand biodiversity patterns.

While secondary data can be a valuable resource for scientific research, it is crucial to recognise and address its limitations and ideally estimate the errors within. Common challenges include species identification accuracy, geographic coordinate precision and data entry errors. In addition, datasets from different studies may differ in their sampling methods, data structure and definitions of key variables, making direct comparisons difficult. Finally, some datasets may not be openly accessible, which has implications for data availability and complicates data access and sharing policies.

Other limitations are the sampling and temporal biases, which can arise when working with secondary data, making data interpretation more challenging. Sampling bias occurs when the data sampling disproportionately favours certain species or areas over others, for example, the concentration of specimen records in more easily accessible sites, such as major cities, roads and navigable rivers (Boakes et al. 2010). In addition, logistics and human interference are factors that can explain research probability (e.g. 64% of research probability in Amazon; Carvalho et al. (2023)). Temporal bias, on the other hand, refers to the uneven distribution of data across time periods. Secondary data sources may include data collected over different time spans, reflecting historical variations in research focus, funding availability or changes in data-recording practices. Consequently, certain time periods may be over-represented, while others may be sparsely covered or entirely absent. Additionally, the difficulty of conducting research in regions with limited accessibility introduces challenges that restrict the ability to gather data from remote areas. Thus, remote regions potentially hosting unique biodiversity hotspots are often under-represented or completely absent from the dataset.

In our study, sampling bias is evident in the *São Marcos Bay* area, where an industrial ship port is located. Thus, most of the data were obtained from environmental monitoring reports in the region linked to the environmental licensing process. These reports conducted in a port area inherently prioritise certain species and ecological aspects more relevant to the licensing process, overlooking other important components of biodiversity. Within our database, it becomes apparent that some species records originate from technical reports that are not easily available. For example, we found 365 species and varieties of phytoplankton in technical reports, but 101 were not previously catalogued on the Brazilian Biodiversity Platform REFLORA (Jardim Botânico do Rio de Janeiro 2024) for the Maranhão region. This underscores the fact that the retrieval of biodiversity data can yield enhancements in the comprehension of species composition existing within the defined geographical area.

## Conclusions

The workflow that we employed has facilitated the retrieval of biodiversity data from the ecologically rich and megadiverse *Golfão Maranhense* region in Maranhão, Brazil. By combining a systematic review approach with standardised worksheets with a Darwin Core base, we were able to effectively search and explore a wide range of scientific articles, technical reports and specialised public repositories. The potential use of secondary data for the advancement of scientific research is significant although it must be taken with care and analysed with precautions observing all bias limitation and filters involved. Many technical survey reports were produced in the *Golfão Maranhense* linked to environmental licensing process for the port and surrounding activities. By using existing datasets, researchers can carry out a wide range of activities which include meta-analyses, comparative studies, ecological modelling and, most of all, building hypotheses and producing experiment designs to monitor diversity in a standardised base. Our study highlights the value of systematic review methods and the need for an approach to address data limitations and biases. Likewise, this method can facilitate collaboration amongst researchers, enable comparative analyses across different datasets and support evidence-based conservation strategies and policy-making.

## Supplementary Material

6D9DC4BE-3907-517C-AF3D-DCB6029C5A84 10.3897/BDJ.12.e133775.suppl1 Supplementary material 1 Keywords Data type table Brief description Keywords used in the systematic review of each biotic group. File: oo_1105742.docx https://binary.pensoft.net/file/1105742 Nubia Marques

5F3B36F0-A023-53A7-90F2-33099EBDB0B5 10.3897/BDJ.12.e133775.suppl2 Supplementary material 2 Table Darwin Core (DwC) Data type table Brief description Table containing the Darwin Core (DwC) standard terms that were used to make the table and extract the information from the bibliographic references previously selected in the systematic review. Label = name of the column in the DwC standard; Definition = Brief definition of what each column means. File: oo_1105743.docx https://binary.pensoft.net/file/1105743 Nubia Marques

FD7DB886-9E05-51AA-92A1-93D141C50185 10.3897/BDJ.12.e133775.suppl3 Supplementary material 3 Flowchart of the Preferred Reporting Items for Systematic Reviews and Meta-Analyses (PRISMA) Data type images Brief description Flowchart of the Preferred Reporting Items for Systematic Reviews and Meta-Analyses (PRISMA) separated by groups showing the process of selecting studies throughout the systematic review. The selection process includes three stages: (1) identifying the database and choosing the papers; (2) scanning the references and selecting the papers to be included; (3) including the selected papers. File: oo_1105747.docx https://binary.pensoft.net/file/1105747 Nubia Marques

1A6094FB-5435-5F3C-8F73-AC20241F0692 10.3897/BDJ.12.e133775.suppl4 Supplementary material 4 List of species from the Golfão Maranhense (Maranhão State, Brazil) Data type Table Brief description List of species from the Golfão Maranhense (Maranhão State, Brazil) that were retrieved through the systematic literature review. File: oo_1105748.docx https://binary.pensoft.net/file/1105748 Nubia Marques
