# Supplementary material for: Retrieving biodiversity data from multiple sources: making secondary data standardised and accessible
Source: Biodivers Data J. 2024 Sep 20;12:e133775. doi: 10.3897/BDJ.12.e133775 (PMC11437127; doi:10.3897/BDJ.12.e133775)
Supplement: Supplementary material 1 — Keywords [file bdj-12-e133775-s001.docx]

**Appendix 1.** Keywords used in the systematic review of each biotic group.

| Group | Keywords |
| --- | --- |
| Amphibian | ("herpetofauna" OR "amphibians" OR "anfíbios") AND ("maranhão"); |
| Benthos | (”macrofauna bentônica” OR “poliquetas” OR “benthic” OR “polychaeta” OR “polychates” OR “Fauna bêntica”) AND (“Maranhão” OR “São Luís” OR “São José bay” OR “São Marcos bay” OR”Amazon coast”); |
| Birds | (bird* OR aves) AND (maranhão OR “amazon coast” OR “litoral amazônico” OR “golfão maranhense” OR “Gulf of Maranhão” OR “Baía de São Marcos” OR “São Marcos Bay” OR “Baía de São José” OR “São José Bay” OR “Amazônia oriental” OR “eastern Amazon”); |
| Fishes | ("Ichthyology ") AND (“São Marcos Bay” OR “São José Bay” OR "Maranhão Gulf" OR "Golfão Maranhense"); |
| Mammals | ("Mammals" OR “Mammalia” OR "Aquatic Mammals" OR "Marine Mammals") AND ("Maranhão" OR "São Luiz do Maranhão" OR "São Luiz Island" OR "Eastern Amazon" OR "Amazonia"); |
| Phytoplankton | ("Phytoplankton" AND "Marina") AND ("Maranhão" OR "São Luís" OR "São José bay" OR "São Marcos bay" OR "Amazon coast"); |
| Plants | (“Angiospermae” OR “Bryophyta” OR “Biodiversity” OR “Flora” OR “Floristics” OR “Forest Inventory” OR ”Gymnospermae” OR “Lycophyta” OR “Monilophyta” OR “Richness” OR “Phytosociological” OR “Plant” OR “Species” OR “Vegetation”) AND ("Maranhão" OR "São Luís" OR "São José bay" OR "São Marcos bay" OR"Amazon coast"); |
| Reptiles | ("herpetofauna" OR "reptiles" OR "répteis" OR "tartaruga marinha" OR "marine turtles" OR "sea turtle" OR "chelonia" OR "crocodylia" OR "squamata") AND ("maranhão"). |
