# Supplementary material for: Retrieving biodiversity data from multiple sources: making secondary data standardised and accessible
Source: Biodivers Data J. 2024 Sep 20;12:e133775. doi: 10.3897/BDJ.12.e133775 (PMC11437127; doi:10.3897/BDJ.12.e133775)
Supplement: Supplementary material 2 — Table Darwin Core (DwC) [file bdj-12-e133775-s002.docx]

**Appendix 2.** Table containing the Darwin Core (DwC) standard terms that were used to make the table and extract the information from the bibliographic references previously selected in the systematic review. Label= name of the column in the DwC standard; Definition= Brief definition of what each column means.

| **Source** | **Label** | **Definition** |
| --- | --- | --- |
| Darwin core | Event ID | An identifier for the set of information associated with an Event (something that occurs at a place and time). May be a global unique identifier or an identifier specific to the data set. |
| Darwin core | Parent Event ID | An identifier for the broader Event that groups this and potentially other Events. |
| Darwin core | Sampling Protocol | The name of, reference to, or description of the method or protocol used during an Event. |
| Darwin core | Sampling Effort | The amount of effort expended during an Event. |
| Darwin core | Sample Size Value | A numeric value for a measurement of the size (time duration, length, area, or volume) of a sample in a sampling event. |
| Darwin core | Sample Size Unit | The unit of measurement of the size (time duration, length, area, or volume) of a sample in a sampling event. |
| Darwin core | Event Date | The date-time or interval during which an Event occurred. For occurrences, this is the date-time when the event was recorded. Not suitable for a time in a geological context. |
| Darwin core | Verbatim Event Date | The verbatim original representation of the date and time information for an Event. |
| Darwin core | Event Remarks | Comments or notes about the Event. |
| Darwin core | Habitat | A category or description of the habitat in which the Event occurred. |
| Darwin core | Country | The name of the country or major administrative unit in which the Location occurs. |
| Darwin core | Country Code | The standard code for the country in which the Location occurs. |
| Darwin core | Locality | The specific description of the place. Less specific geographic information can be provided in other geographic terms (higherGeography, continent, country, stateProvince, county, municipality, waterBody, island, islandGroup). This term may contain information modified from the original to correct perceived errors or standardize the description. |
| Darwin core | Location ID | An identifier for the set of location information (data associated with dcterms- Location). May be a global unique identifier or an identifier specific to the data set. |
| Darwin core | Location Remarks | Comments or notes about the Location. |
| Darwin core | Decimal Latitude | The geographic latitude (in decimal degrees, using the spatial reference system given in geodeticDatum) of the geographic center of a Location. Positive values are north of the Equator, negative values are south of it. Legal values lie between -90 and 90, inclusive. |
| Darwin core | Decimal Longitude | The geographic longitude (in decimal degrees, using the spatial reference system given in geodeticDatum) of the geographic center of a Location. Positive values are east of the Greenwich Meridian, negative values are west of it. Legal values lie between -180 and 180, inclusive. |
| Darwin core | Geodetic Datum | The ellipsoid, geodetic datum, or spatial reference system (SRS) upon which the geographic coordinates given in decimalLatitude and decimalLongitude as based. |
| Darwin core | Georeference Remarks | Notes or comments about the spatial description determination, explaining assumptions made in addition or opposition to the those formalized in the method referred to in georeferenceProtocol. |
| Darwin core | Verbatim Coordinate System | The coordinate format for the verbatimLatitude and verbatimLongitude or the verbatimCoordinates of the Location. |
| Darwin core | Verbatim Latitude | The verbatim original latitude of the Location. The coordinate ellipsoid, geodeticDatum, or full Spatial Reference System (SRS) for these coordinates should be stored in verbatimSRS and the coordinate system should be stored in verbatimCoordinateSystem. |
| Darwin core | Verbatim Longitude | The verbatim original longitude of the Location. The coordinate ellipsoid, geodeticDatum, or full Spatial Reference System (SRS) for these coordinates should be stored in verbatimSRS and the coordinate system should be stored in verbatimCoordinateSystem. |
| Darwin core | Water Body | The name of the water body in which the Location occurs. |
| Darwin core | Georeferenced By | A list (concatenated and separated) of names of people, groups, or organizations who determined the georeference (spatial representation) for the Location. |
| Darwin core | Owner Institution Code | The name (or acronym) in use by the institution having ownership of the object(s) or information referred to in the record. |
| Darwin core | Event ID | An identifier for the set of information associated with an Event (something that occurs at a place and time). May be a global unique identifier or an identifier specific to the data set. |
| Darwin core | Occurrence ID | An identifier for the Occurrence (as opposed to a particular digital record of the occurrence). In the absence of a persistent global unique identifier, construct one from a combination of identifiers in the record that will most closely make the occurrenceID globally unique. |
| Darwin core | Basis of Record | The specific nature of the data record. |
| Darwin core | Occurrence Remarks | Comments or notes about the Occurrence. |
| Darwin core | Individual Count | The number of individuals represented present at the time of the Occurrence. |
| Darwin core | Organism Quantity | A number or enumeration value for the quantity of organisms. |
| Darwin core | Organism Quantity Type | The type of quantification system used for the quantity of organisms. |
| Darwin core | Occurrence Status | A statement about the presence or absence of a Taxon at a Location. |
| Darwin core | Scientific Name | The full scientific name, with authorship and date information if known. When forming part of an Identification, this should be the name in lowest level taxonomic rank that can be determined. This term should not contain identification qualifications, which should instead be supplied in the IdentificationQualifier term. |
| Darwin core | Kingdom | The full scientific name of the kingdom in which the taxon is classified. |
| Darwin core | Phylum | The full scientific name of the phylum or division in which the taxon is classified. |
| Darwin core | Class | The full scientific name of the class in which the taxon is classified. |
| Darwin core | Order | The full scientific name of the order in which the taxon is classified. |
| Darwin core | Family | The full scientific name of the family in which the taxon is classified. |
| Darwin core | Genus | The full scientific name of the genus in which the taxon is classified. |
| Darwin core | Subgenus | The full scientific name of the subgenus in which the taxon is classified. Values should include the genus to avoid homonym confusion. |
| Darwin core | Taxon Rank | The taxonomic rank of the most specific name in the scientificName. |
| Darwin core | Name Published In | A reference for the publication in which the scientificName was originally established under the rules of the associated nomenclaturalCode. |
| Darwin core | Name Published In Year | The four-digit year in which the scientificName was published. |
| Darwin core | Taxon Remarks | Comments or notes about the taxon or name. |
| Darwin core | Identification Remarks | Comments or notes about the Identification. |
| Darwin core | Recorded By | A list (concatenated and separated) of names of people, groups, or organizations responsible for recording the original Occurrence. The primary collector or observer, especially one who applies a personal identifier (recordNumber), should be listed first. |
| Darwin core | Disposition | The current state of a specimen with respect to the collection identified in collectionCode or collectionID. |
| Darwin core | Establishment Means | Statement about whether an organism or organisms have been introduced to a given place and time through the direct or indirect activity of modern humans. |
| Darwin core | Life Stage | The age class or life stage of the biological individual(s) at the time the Occurrence was recorded. |
| Darwin core | Reproductive Condition | The reproductive condition of the biological individual(s) represented in the Occurrence. |
| Darwin core | Sex | The sex of the biological individual(s) represented in the Occurrence. |
| Darwin core | Associated Media | A list (concatenated and separated) of identifiers (publication, global unique identifier, URI) of media associated with the Occurrence. |
| Darwin core | Associated References | A list (concatenated and separated) of identifiers (publication, bibliographic reference, global unique identifier, URI) of literature associated with the Occurrence. |
| Darwin core | Associated Sequences | A list (concatenated and separated) of identifiers (publication, global unique identifier, URI) of genetic sequence information associated with the Occurrence. |
| Darwin core | Collection Code | The name, acronym, coden, or initialism identifying the collection or data set from which the record was derived. |
| Darwin core | Collection ID | An identifier for the collection or dataset from which the record was derived. |
| Darwin core | Field Number | An identifier given to the event in the field. Often serves as a link between field notes and the Event. |
| Darwin core | Owner Institution Code | The name (or acronym) in use by the institution having ownership of the object(s) or information referred to in the record. |
| Additional terms | statusConsIUCN | Status of species conservation according to International Union for Conservation of Nature (IUCN) |
| Additional terms | statusConsMMA | The status "Endandered" and "Vulnerable" follow PORTARIA MMA Nº 148, DE 7 DE JUNHO DE 2022, while "DataDeficient", "LeastConcern", "Not Evaluated" and "Near Threatened" follow MMA 2014. |
| Additional terms | ID | Numeric identification |
| Additional terms | Reference | Complete reference |
| Additional terms | Year | Publication year |
| Additional terms | Keywords | Representative keywords of the work |
| Additional terms | Open access | Open access article |
| Additional terms | Target Group | Target group of the study |
| Additional terms | Target Taxon | Target taxon of the study |
| Additional terms | Target Environment | Target study environment |
| Additional terms | Project Purpose | Research purpose |
| Additional terms | Type of contribution | Type of the contribution to the review |
| Additional terms | Observation | Extras notes |
