# Supplementary material for: Retrieving biodiversity data from multiple sources: making secondary data standardised and accessible
Source: Biodivers Data J. 2024 Sep 20;12:e133775. doi: 10.3897/BDJ.12.e133775 (PMC11437127; doi:10.3897/BDJ.12.e133775)
Supplement: Supplementary material 3 — Flowchart of the Preferred Reporting Items for Systematic Reviews and Meta-Analyses (PRISMA) [file bdj-12-e133775-s003.docx]

**Appendix 4**. List of species from the *Golfão Maranhense* (*Maranhão State*, Brazil) that were retrieved through the systematic literature review.

| **Group** | **Taxon** | **Taxon rank** |
| --- | --- | --- |
| Amphibia | Boana Gray, 1825 | genus |
| Amphibia | Dendropsophus Fitzinger, 1843 | genus |
| Amphibia | Dendropsophus gr. Marmoratus | genus |
| Amphibia | Dendropsophus gr. Microcephalus | genus |
| Amphibia | Elachistocleis Parker, 1927 | genus |
| Amphibia | Physalaemus Fitzinger, 1826 | genus |
| Amphibia | Pseudopaludicola Miranda-Ribeiro, 1926 | genus |
| Amphibia | Rhinella Fitzinger, 1826 | genus |
| Amphibia | Scinax Wagler, 1830 | genus |
| Amphibia | Adelphobates galactonotus (Steindachner, 1864) | species |
| Amphibia | Adenomera andreae (Müller, 1923) | species |
| Amphibia | Adenomera hylaedactyla (Cope, 1868) | species |
| Amphibia | Boana albopunctata (Spix, 1824) | species |
| Amphibia | Boana geographica (Spix, 1824) | species |
| Amphibia | Boana multifasciata (Günther, 1859) | species |
| Amphibia | Boana punctata (Schneider, 1799) | species |
| Amphibia | Boana raniceps (Cope, 1862) | species |
| Amphibia | Caecilia gracilis Shaw, 1802 | species |
| Amphibia | Dendropsophus branneri (Cochran, 1948) | species |
| Amphibia | Dendropsophus decipiens (Lutz, 1925) | species |
| Amphibia | Dendropsophus leucophyllatus (Beireis, 1783) | species |
| Amphibia | Dendropsophus melanargyreus (Cope, 1887) | species |
| Amphibia | Dendropsophus microcephalus (Cope, 1886) | species |
| Amphibia | Dendropsophus minutus (Peters, 1872) | species |
| Amphibia | Dendropsophus nanus (Boulenger, 1889) | species |
| Amphibia | Dendropsophus soaresi (Caramaschi and Jim, 1983) | species |
| Amphibia | Elachistocleis "ovalis" (Schneider, 1799) | species |
| Amphibia | Elachistocleis bumbameuboi Caramaschi, 2010 | species |
| Amphibia | Elachistocleis piauiensis Caramaschi and Jim, 1983 | species |
| Amphibia | Hydrolaetare schmidti (Cochran and Goin, 1959) | species |
| Amphibia | Leptodactylus caatingae Heyer and Juncá, 2003 | species |
| Amphibia | Leptodactylus fuscus (Schneider, 1799) | species |
| Amphibia | Leptodactylus labyrinthicus (Spix, 1824) | species |
| Amphibia | Leptodactylus latrans (Steffen, 1815) | species |
| Amphibia | Leptodactylus macrosternum Miranda-Ribeiro, 1926 | species |
| Amphibia | Leptodactylus mystaceus (Spix, 1824) | species |
| Amphibia | Leptodactylus pentadactylus (Laurenti, 1768) | species |
| Amphibia | Leptodactylus petersii (Steindachner, 1864) | species |
| Amphibia | Leptodactylus podicipinus (Cope, 1862) | species |
| Amphibia | Leptodactylus pustulatus (Peters, 1870) | species |
| Amphibia | Leptodactylus troglodytes Lutz, 1926 | species |
| Amphibia | Leptodactylus vastus Lutz, 1930 | species |
| Amphibia | Osteocephalus leprieurii (Duméril and Bibron, 1841) | species |
| Amphibia | Osteocephalus taurinus Steindachner, 1862 | species |
| Amphibia | Physalaemus centralis Bokermann, 1962 | species |
| Amphibia | Physalaemus cuvieri Fitzinger, 1826 | species |
| Amphibia | Pipa pipa (Linnaeus, 1758) | species |
| Amphibia | Pithecopus hypochondrialis (Daudin, 1800) | species |
| Amphibia | Pseudis paradoxa (Linnaeus, 1758) | species |
| Amphibia | Pseudopaludicola mystacalis (Cope, 1887) | species |
| Amphibia | Rhinella diptycha (Cope, 1862) | species |
| Amphibia | Rhinella granulosa (Spix, 1824) | species |
| Amphibia | Rhinella margaritifera (Laurenti, 1768) | species |
| Amphibia | Rhinella marina (Linnaeus, 1758) | species |
| Amphibia | Rhinella mirandaribeiroi (Gallardo, 1965) | species |
| Amphibia | Scinax eurydice (Bokermann, 1968) | species |
| Amphibia | Scinax fuscomarginatus (Lutz, 1925) | species |
| Amphibia | Scinax nebulosus (Spix, 1824) | species |
| Amphibia | Scinax ruber (Laurenti, 1768) | species |
| Amphibia | Scinax similis (Cochran, 1952) | species |
| Amphibia | Scinax x-signatus (Spix, 1824) | species |
| Amphibia | Siphonops paulensis Boettger, 1892 | species |
| Amphibia | Sphaenorhynchus lacteus (Daudin, 1800) | species |
| Amphibia | Trachycephalus typhonius (Linnaeus, 1758) | species |
| Benthos | Bivalvia Linnaeus, 1758 | class |
| Benthos | Boonea jadisi (Olsson & McGinty, 1958) | class |
| Benthos | Copepoda Milne Edwards, 1840 | class |
| Benthos | Echinoidea Schumacher, 1817 | class |
| Benthos | Gastropoda Cuvier, 1795 | class |
| Benthos | Hexapoda Latreille, 1825 | class |
| Benthos | Holothuroidea Blainville, 1834 | class |
| Benthos | Hydrozoa Owen, 1843 | class |
| Benthos | Janthina exigua Lamarck, 1816 | class |
| Benthos | Nemertea Schultze, 1851 | class |
| Benthos | Oligochaeta Grube, 1850 | class |
| Benthos | Oligochaeta Grube, 1850 | class |
| Benthos | Ophiuroidea Gray, 1840 | class |
| Benthos | Ostracoda Latreille, 1802 | class |
| Benthos | Peracarida Calman, 1904 | class |
| Benthos | Polychaeta Grube, 1850 | class |
| Benthos | Scaphopoda Bronn, 1862 | class |
| Benthos | Scleractinia Bourne, 1900 | class |
| Benthos | Turbellaria Ehrenberg, 1831 | class |
| Benthos | Turbellaria; Ehrenberg, 1831 | class |
| Benthos | Ampharetidae Malmgren, 1866 | family |
| Benthos | Capitellidae Grube, 1862 | family |
| Benthos | Ceratopogonidae Newman, 1834 | family |
| Benthos | Chironomidae Newman, 1834 | family |
| Benthos | Cirratulidae Ryckholt, 1851 | family |
| Benthos | Dolichopodidae Latreille, 1809 | family |
| Benthos | Dytiscidae Leach, 1815 | family |
| Benthos | Eulepethidae Chamberlin, 1919 | family |
| Benthos | Eusyllinae Malaquin, 1893 | family |
| Benthos | Goniadidae Kinberg, 1866 | family |
| Benthos | Ischyroceridae Stebbing, 1899 | family |
| Benthos | Lumbrineridae Schmarda, 1861 | family |
| Benthos | Maldanidae Malmgren, 1867 | family |
| Benthos | Melitidae Bousfield, 1973 | family |
| Benthos | Mesoveliidae Douglas & Scott, 1867 | family |
| Benthos | Muricidae Rafinesque, 1815 | family |
| Benthos | Muscidae Latreille, 1802 | family |
| Benthos | Nemertea Schultze, 1851 | family |
| Benthos | Nephtyidae Grube, 1850 | family |
| Benthos | Oenonidae Kinberg, 1865 | family |
| Benthos | Onuphidae Kinberg, 1865 | family |
| Benthos | Opheliidae Malmgren, 1867 | family |
| Benthos | Orbiniidae Hartman, 1942 | family |
| Benthos | Owenia Delle Chiaje, 1844 | family |
| Benthos | Paraonidae Cerruti, 1909 | family |
| Benthos | Penaeidae Rafinesque, 1815 | family |
| Benthos | Phoronida Hatschek, 1888 | family |
| Benthos | Phoxocephalidae G.O. Sars, 1891 | family |
| Benthos | Psychodidae Newman, 1834 | family |
| Benthos | Ptilodactylidae Laporte de Castelnau, 1836 | family |
| Benthos | Sabellariidae Johnston, 1865 | family |
| Benthos | Sigalionidae Kinberg, 1856 | family |
| Benthos | Sphaeromatidae Latreille, 1825 | family |
| Benthos | Syllidae Grube, 1850 | family |
| Benthos | Tabanidae Latreille, 1802 | family |
| Benthos | Terebellidae Johnston, 1846 | family |
| Benthos | Abyssoninoe Orensanz, 1990 | genus |
| Benthos | Alitta Kinberg, 1865 | genus |
| Benthos | Aplysia Linnaeus, 1767 | genus |
| Benthos | Arabella Grube, 1850 | genus |
| Benthos | Aricidea (Aedicira) Hartman, 1957 | genus |
| Benthos | Aricidea Webster, 1879 | genus |
| Benthos | Armandia Filippi, 1861 | genus |
| Benthos | Barleeia W. Clark, 1853 | genus |
| Benthos | Benthonella Dall, 1889 | genus |
| Benthos | Blauneria Shuttleworth, 1854 | genus |
| Benthos | Branchiomma Kölliker, 1858 | genus |
| Benthos | Capitella Blainville, 1828 | genus |
| Benthos | Caprella Lamarck, 1801 | genus |
| Benthos | Carcinus Leach, 1814 | genus |
| Benthos | Cerapus Say, 1817 | genus |
| Benthos | Ceratia H. Adams & A. Adams, 1852 | genus |
| Benthos | Cerithiopsis Forbes & Hanley, 1850 | genus |
| Benthos | Chone Krøyer, 1856 | genus |
| Benthos | Claviramus Fitzhugh, 2002 | genus |
| Benthos | Coralliophila H. Adams & A. Adams, 1853 | genus |
| Benthos | Crassostrea Sacco, 1897 | genus |
| Benthos | Cyclostremiscus Pilsbry & Olsson, 1945 | genus |
| Benthos | Cylichna Lovén, 1846 | genus |
| Benthos | Cyrenoida Joannis, 1835 | genus |
| Benthos | Diopatra Audouin & Milne Edwards, 1833 | genus |
| Benthos | Diopatra Audouin & Milne Edwards, 1833 | genus |
| Benthos | Dorvillea Parfitt, 1866 | genus |
| Benthos | Dorvillea Parfitt, 1866 | genus |
| Benthos | Eulalia Savigny, 1822 | genus |
| Benthos | Eulimella Forbes & M'Andrew, 1846 | genus |
| Benthos | Eunoe Malmgren, 1865 | genus |
| Benthos | Eurydice Leach, 1815 | genus |
| Benthos | Eurytellina P. Fischer, 1887 | genus |
| Benthos | Exogone Örsted, 1845 | genus |
| Benthos | Gammarus Fabricius, 1775 | genus |
| Benthos | Glycera Lamarck, 1818 | genus |
| Benthos | Glycinde Müller, 1858 | genus |
| Benthos | Goniada Audouin & H Milne Edwards, 1833 | genus |
| Benthos | Gyptis Marion, 1874 | genus |
| Benthos | Heleobia W. Stimpson, 1865 | genus |
| Benthos | Hemipodia Kinberg, 1865 | genus |
| Benthos | Heteromastus Eisig, 1887 | genus |
| Benthos | Hypereteone Bergström, 1914 | genus |
| Benthos | Kinbergonuphis Fauchald, 1982 | genus |
| Benthos | Laeonereis Hartman, 1945 | genus |
| Benthos | Leiocapitella Hartman, 1947 | genus |
| Benthos | Leiocapitellides Hartmann-Schröder, 1960 | genus |
| Benthos | Leitoscoloplos Day, 1977 | genus |
| Benthos | Lepidonotus Leach, 1816 | genus |
| Benthos | Lumbrineriopsis Orensanz, 1973 | genus |
| Benthos | Lumbrineris Blainville, 1828 | genus |
| Benthos | Lysarete Kinberg, 1865 | genus |
| Benthos | Magelona F. Müller, 1858 | genus |
| Benthos | Magelona F. Müller, 1858 | genus |
| Benthos | Mediomastus Hartman, 1944 | genus |
| Benthos | Micronephthys Friedrich, 1939 | genus |
| Benthos | Miralda A. Adams, 1863 | genus |
| Benthos | Modulus Gray, 1842 | genus |
| Benthos | Monocorophium Bousfield & Hoover, 1997 | genus |
| Benthos | Nassarius Duméril, 1805 | genus |
| Benthos | Natica Scopoli, 1777 | genus |
| Benthos | Natica Scopoli, 1777 | genus |
| Benthos | Neanthes Kinberg, 1865 | genus |
| Benthos | Nephtys Cuvier, 1817 | genus |
| Benthos | Nereis Linnaeus, 1758 | genus |
| Benthos | Nereis Linnaeus, 1758 | genus |
| Benthos | Nicon Kinberg, 1865 | genus |
| Benthos | Nonatus Amaral, 1980 | genus |
| Benthos | Notomastus M. Sars, 1851 | genus |
| Benthos | Notomastus M. Sars, 1851 | genus |
| Benthos | Nucula Lamarck, 1799 | genus |
| Benthos | Nuculana Link, 1807 | genus |
| Benthos | Ocypode Weber, 1795 | genus |
| Benthos | Odontosyllis Claparède, 1863 | genus |
| Benthos | Odostomia J. Fleming, 1813 | genus |
| Benthos | Ogyrides Stebbing, 1914 | genus |
| Benthos | Olivella Swainson, 1831 | genus |
| Benthos | Ophelia Savigny, 1822 | genus |
| Benthos | Orbinia Quatrefages, 1866 | genus |
| Benthos | Paradoneis Hartman, 1965 | genus |
| Benthos | Paranaitis Southern, 1914 | genus |
| Benthos | Paraonis Grube, 1873 | genus |
| Benthos | Phyllodoce Lamarck, 1818 | genus |
| Benthos | Phylo Kinberg, 1866 | genus |
| Benthos | Polydora Bosc, 1802 | genus |
| Benthos | Polyophthalmus Quatrefages, 1850 | genus |
| Benthos | Sabellaria wilsoni Lana & Gruet, 1989 | genus |
| Benthos | Samoana Pilsbry, 1909 | genus |
| Benthos | Sayella Dall, 1885 | genus |
| Benthos | Scolelepis Blainville, 1828 | genus |
| Benthos | Scoloplos Blainville, 1828 | genus |
| Benthos | Scoloplos Blainville, 1828 | genus |
| Benthos | Sigambra Müller, 1858 | genus |
| Benthos | Spiophanes Grube, 1860 | genus |
| Benthos | Syllis Lamarck, 1818 | genus |
| Benthos | Tellina Linnaeus, 1758 | genus |
| Benthos | Terebella Linnaeus, 1767 | genus |
| Benthos | Tharyx Webster & Benedict, 1887 | genus |
| Benthos | Trochochaeta Levinsen, 1884 | genus |
| Benthos | Trypanosyllis Claparède, 1864 | genus |
| Benthos | Tubifex Lamarck, 1816 | genus |
| Benthos | Turbonilla Risso, 1826 | genus |
| Benthos | Upogebia Leach, 1814 | genus |
| Benthos | Urothoe Dana, 1852 | genus |
| Benthos | Volvarina Hinds, 1844 | genus |
| Benthos | Incertae sedis | Kingdom |
| Benthos | Actinaria  Blainville, 1830 | order |
| Benthos | Actiniaria, Hertwig, 1882 | order |
| Benthos | Amphipoda Latreille, 1816 | order |
| Benthos | Anomura MacLeay, 1838 | order |
| Benthos | Brachyura, Latreille, 1802 | order |
| Benthos | Cumacea Krøyer, 1846 | order |
| Benthos | Decapoda Latreille, 1802 | order |
| Benthos | Diptera Linnaeus, 1758 | order |
| Benthos | Hymenoptera Linnaeus, 1758 | order |
| Benthos | Insecta Linnaeus, 1758 | order |
| Benthos | Isopoda Latreille, 1817 | order |
| Benthos | Isoptera Brullé, 1832 | order |
| Benthos | Nemertea Schultze, 1851 | order |
| Benthos | Nudibranchia Cuvier, 1817 | order |
| Benthos | Oligochaeta Grube, 1850 | order |
| Benthos | Peracarida Calman, 1904 | order |
| Benthos | Scleractinia Bourne, 1900 | order |
| Benthos | Sipuncula Stephen, 1965 | order |
| Benthos | Stomatopoda Latreille, 1817 | order |
| Benthos | Tanaidacea Dana, 1849 | order |
| Benthos | Bryozoa Ehrenberg, 1831 | phylum |
| Benthos | Bryozoa Ehrenberg, 1831 | phylum |
| Benthos | Chaetognatha Leuckart, 1894 | phylum |
| Benthos | Cnidaria Hatschek, 1888 | phylum |
| Benthos | Crustacea Brünnich, 1772 | phylum |
| Benthos | Foraminifera d'Orbigny, 1826 | phylum |
| Benthos | Hemichordata Bateson, 1885 | phylum |
| Benthos | Nematoda Potts, 1932 | phylum |
| Benthos | Nematoda Potts, 1932 | phylum |
| Benthos | Nematomorpha vejdovsky, 1886 | phylum |
| Benthos | Nemertea Schultze, 1851 | phylum |
| Benthos | Ophiuroidea Gray, 1840 | phylum |
| Benthos | Sipuncula Stephen, 1965 | phylum |
| Benthos | Tunicata Lamarck, 1816 | phylum |
| Benthos | Turbinidae Rafinesque, 1815 | phylum |
| Benthos | Aclis underwoodae Bartsch, 1947 | species |
| Benthos | Alitta succinea (Leuckart, 1847) | species |
| Benthos | Alitta succinea (Leuckart, 1847) | species |
| Benthos | Alvania electa (Monterosato, 1874) | species |
| Benthos | Amarilladesma mactroides (Reeve, 1854) | species |
| Benthos | Ameritella diantha (Boss, 1964) | species |
| Benthos | Ammonia beccarii (Linnaeus, 1758) | species |
| Benthos | Ammothella longipes (Hodge, 1864) | species |
| Benthos | Amphibalanus amphitrite (Darwin, 1854) | species |
| Benthos | Amphibalanus venustus (Darwin, 1854) | species |
| Benthos | Amphiura joubini Koehler, 1912 | species |
| Benthos | Anadara brasiliana (Lamarck, 1819) | species |
| Benthos | Anadara notabilis (Röding, 1798) | species |
| Benthos | Anadara secernenda (E. Lamy, 1907) | species |
| Benthos | Anomalocardia flexuosa (Linnaeus, 1767) | species |
| Benthos | Anomalocardia flexuosa (Linnaeus, 1767) | species |
| Benthos | Anoplodactylus eroticus Stock, 1968 | species |
| Benthos | Arabella aracaensis Steiner & Amaral, 2009 | species |
| Benthos | Arabella iricolor (Montagu, 1804) | species |
| Benthos | Aricidea (Acmira) catherinae Laubier, 1967 | species |
| Benthos | Aricidea (Acmira) taylori Pettibone, 1965 | species |
| Benthos | Aricidea (Aricidea) albatrossae Pettibone, 1957 | species |
| Benthos | Aricidea (Aricidea) albatrossae Pettibone, 1957 | species |
| Benthos | Armandia hossfeldi Hartmann-Schröder, 1956 | species |
| Benthos | Armandia hossfeldi Hartmann-Schröder, 1956 | species |
| Benthos | Austinixa aidae (Righi, 1967) | species |
| Benthos | Austromacoma constricta (Bruguière, 1792) | species |
| Benthos | Austromacoma constricta (Bruguière, 1792) | species |
| Benthos | Blauneria heteroclita (Montagu, 1808 | species |
| Benthos | Boonea jadisi (Olsson & McGinty, 1958) | species |
| Benthos | Bowmaniella dissimilis (Coifmann, 1937) | species |
| Benthos | Bugula neritina (Linnaeus, 1758) | species |
| Benthos | Caecum achirona (de Folin, 1867) | species |
| Benthos | Caecum strigosum de Folin, 1868 | species |
| Benthos | Callinectes sapidus Rathbun, 1896 | species |
| Benthos | Calyptraea centralis (Conrad, 1841) | species |
| Benthos | Capitella aracaensis Silva et al., 2017 | species |
| Benthos | Capitella aracaensis Silva & Amaral, 2017 | species |
| Benthos | Capitella biota Silva & Amaral, 2017 | species |
| Benthos | Caprella penantis Leach, 1814 | species |
| Benthos | Caryocorbula swiftiana (C. B. Adams, 1852) | species |
| Benthos | Casmaria ponderosa (Gmelin, 1791) | species |
| Benthos | Ceratia rustica (Watson, 1885) | species |
| Benthos | Cerithiopsis lata (C. B. Adams, 1850) | species |
| Benthos | Cerithium atratum (Born, 1778) | species |
| Benthos | Chione cancellata (Linnaeus, 1767) | species |
| Benthos | Chione subrostrata Lamarck, 1818 | species |
| Benthos | Chione pubera (Bory Saint-Vincent,1827) | species |
| Benthos | Chlamydopleon dissimile (Coifmann, 1937) | species |
| Benthos | Circulus semisculptus (Olsson & McGinty, 1958) | species |
| Benthos | Clathrolucina costata (d'Orbigny, 1846) | species |
| Benthos | Clythrocerus carinatus Coelho, 1973 | species |
| Benthos | Coleoptera linnaeus, 1758 | species |
| Benthos | Coralliophila salebrosa H. Adams & A. Adams, 1864 | species |
| Benthos | Cossura yacy Sousa, Nogueira, Cutrim & Oliveira, 2019 | species |
| Benthos | Crassinella martinicensis (d'Orbigny, 1853) | species |
| Benthos | Crassostrea rhizophorae (Guilding, 1828) | species |
| Benthos | Crepidula protea d'Orbigny, 1841 | species |
| Benthos | Cronius ruber (Lamarck, 1818) | species |
| Benthos | Ctena orbiculata (Montagu, 1808) | species |
| Benthos | Dispio remanei Friedrich, 1956 | species |
| Benthos | Donax gemmula J. P. E. Morrison, 1971 | species |
| Benthos | Donax striatus Linnaeus, 1767 | species |
| Benthos | Donax trunculus Linnaeus, 1758 | species |
| Benthos | Ellobium dominicense (Férussac, 1821) | species |
| Benthos | Eurytellina lineata (W. Turton, 1819) | species |
| Benthos | Eurytellina punicea (Born, 1778) | species |
| Benthos | Excirolana armata (Dana, 1853) | species |
| Benthos | Excirolana braziliensis Richardson, 1912 | species |
| Benthos | Exogone Örsted, 1845 | species |
| Benthos | Exolaternula spengleri (Gmelin, 1791) | species |
| Benthos | Falsuszafrona pulchella (Blainville, 1829) | species |
| Benthos | Gammarus crinicornis Stock, 1966 | species |
| Benthos | Glycinde multidens Müller, 1858 | species |
| Benthos | Glycinde multidens Müller, 1858 | species |
| Benthos | Glypteuthria meridionalis (E. A. Smith, 1881) | species |
| Benthos | Goniada littorea Hartman, 1950 | species |
| Benthos | Goniadides carolinae Day, 1973 | species |
| Benthos | Graptacme perlonga (Dall, 1881) | species |
| Benthos | Grubeulepis serrata Cutrim, Praseres, Conceição, De Almeida, Júnior & De Oliveira, 2018 | species |
| Benthos | Grubeulepis serrata Cutrim, Praseres, Conceição, De Almeida, Júnior & De Oliveira, 2018 | species |
| Benthos | Heleobia australis (d'Orbigny, 1835) | species |
| Benthos | Hemiaegina minuta Mayer, 1890 | species |
| Benthos | Hemipholis elongata (Say, 1825) | species |
| Benthos | Hemipodia californiensis (Hartman, 1938) | species |
| Benthos | Hemipodia simplex (Grube, 1857) | species |
| Benthos | Hermundura tricuspis Müller, 1858 | species |
| Benthos | Heterodonax pacificus (Conrad, 1837) | species |
| Benthos | Homola barbata (Fabricius, 1793) | species |
| Benthos | Iphigenia brasiliensis (Lamarck, 181 | species |
| Benthos | Isolda pulchella Müller in Grube, 1858 | species |
| Benthos | Laeonereis culveri (Webster, 1879) | species |
| Benthos | Leodamas rubrus (Webster, 1879) | species |
| Benthos | Leodamas texana (Maciolek & Holland, 1978) | species |
| Benthos | Leptuca thayeri (Rathbun, 1900) | species |
| Benthos | Leucothoe incisa (Robertson, 1892) | species |
| Benthos | Leucothoe incisa Robertson, 1892 | species |
| Benthos | Littoraria angulifera (Lamarck, 1822) | species |
| Benthos | Littoraria angulifera (Lamarck, 1822) | species |
| Benthos | Littoraria flava (P. P. King, 1832) | species |
| Benthos | Macoploma tenta (Say, 1838) | species |
| Benthos | Mactra isabelleana d'Orbigny, 1846 | species |
| Benthos | Magelona papillicornis F. Müller, 1858 | species |
| Benthos | Magelona posterelongata Bolívar & Lana, 1986 | species |
| Benthos | Magelona riojai Jones, 1963 | species |
| Benthos | Marphysa sanguinea (Montagu, 1813) | species |
| Benthos | Mediomastus californiensis Hartman, 1944 | species |
| Benthos | Melampus coffea (Linnaeus, 1758) | species |
| Benthos | Membranipora membranacea (Linnaeus, 1767) | species |
| Benthos | Microcardium tinctum (Dall, 1881) | species |
| Benthos | Minuca burgersi (Holthuis, 1967) | species |
| Benthos | Minuca rapax (Smith, 1870) | species |
| Benthos | Monia nobilis (Reeve, 1859) | species |
| Benthos | Monocorophium acherusicum (Costa, 1853) | species |
| Benthos | Monokalliapseudes schubarti (Mañé-Garzón, 1949) | species |
| Benthos | Mytella guyanensis (Lamarck, 1819) | species |
| Benthos | Mytella strigata (Hanley, 1843) | species |
| Benthos | Naineris dendritica (Kinberg, 1867) | species |
| Benthos | Namalycastis abiuma (Grube, 1872) | species |
| Benthos | Natica marochiensis (Gmelin, 1791) | species |
| Benthos | Natica vitellus (Linnaeus, 1758) | species |
| Benthos | Nephtys californiensis Hartman, 1938 | species |
| Benthos | Nephtys fluviatilis Monro, 1937 | species |
| Benthos | Nephtys simoni Perkins, 1980 | species |
| Benthos | Nephtys simoni Perkins, 1980 | species |
| Benthos | Nereis oligohalina (Rioja, 1946) | species |
| Benthos | Nuculana concentrica (Say, 1824) | species |
| Benthos | Olivella minuta (Link, 1807) | species |
| Benthos | Onuphis eremita oculata Hartman, 1951 | species |
| Benthos | Ophiactis lymani Ljungman, 1872 | species |
| Benthos | Ophionereis squamulosa Koehler, 1914 | species |
| Benthos | Owenia fusiformis Delle Chiaje, 1844 | species |
| Benthos | Panopeus lacustris Desbonne in Desbonne & Schramm, 1867 | species |
| Benthos | Panopeus occidentalis de Saussure, 1857 | species |
| Benthos | Paradentalium infractum (Odhner, 1931) | species |
| Benthos | Paranaitis chitinosa Oliveira, Eibye-Jacobsen & Lana, 2018 | species |
| Benthos | Parvanachis obesa (C. B. Adams, 1845 | species |
| Benthos | Parvanachis obesa (C. B. Adams, 1845) | species |
| Benthos | Pendaloma otohimeae (Habe, 1952) | species |
| Benthos | Pentapycnon geayi Bouvier, 1911 | species |
| Benthos | Perinereis anderssoni Kinberg, 1865 | species |
| Benthos | Perinereis vancaurica (Ehlers, 1868) | species |
| Benthos | Petrolisthes amoenus (Guérin-Méneville, 1855) | species |
| Benthos | Phacoides pectinatus (Gmelin, 1791) | species |
| Benthos | Phimochirus occlusus (Henderson, 1888) | species |
| Benthos | Phoxocephalidae G.O. Sars, 1891 | species |
| Benthos | Phyllodoce brasiliensis Oliveira, Magalhães & Lana, 2021 | species |
| Benthos | Phyllodoce tupana Oliveira, Magalhães & Lana, 2021 | species |
| Benthos | Podocerus brasiliensis (Dana, 1853) | species |
| Benthos | Poroeponides lateralis (Terquem, 1878) | species |
| Benthos | Prionospio cirrifera Wirén, 1883 | species |
| Benthos | Progoniada regularis Hartman, 1965 | species |
| Benthos | Protothaca pectorina (Lamarck, 1818) | species |
| Benthos | Psammotella cruenta ([Lightfoot], 1786) | species |
| Benthos | Psammotreta brevifrons (Say, 1834) | species |
| Benthos | Rissoella caribaea Rehder, 1943 | species |
| Benthos | Sabellaria wilsoni Lana & Gruet, 1989 | species |
| Benthos | Scolelepis goodbodyi (Jones, 1962) | species |
| Benthos | Scoletoma atlantica (Kinberg, 1865) | species |
| Benthos | Scoletoma tetraura (Schmarda, 1861) | species |
| Benthos | Scoloplos capensis (Day, 1961) | species |
| Benthos | Scoloplos maranhensis Oliveira, Cutrim, Vieira, Ferreira, Almeida & Nogueira Júnior, 2019 | species |
| Benthos | Semele proficua (Pulteney, 1799) | species |
| Benthos | Semele radiata (Say, 1826) | species |
| Benthos | Sesarma crassipes Cano, 1889 | species |
| Benthos | Sigambra grubei (Müller, 1858) | species |
| Benthos | Solariorbis schumoi (Vanatta, 1913) | species |
| Benthos | Sphenia fragilis (H. Adams & A. Adams, 1854) | species |
| Benthos | Spiophanes duplex (Chamberlin, 1919) | species |
| Benthos | Spisiphon didymum (Watson, 1879) | species |
| Benthos | Stenothoe marina (Spence Bate, 1857) | species |
| Benthos | Stramonita brasiliensis Claremont & D. G. Reid, 2011 | species |
| Benthos | Stramonita brasiliensis Claremont & D. Reid, 2011 | species |
| Benthos | Stramonita haemastoma (Linnaeus, 1767) | species |
| Benthos | Streblospio benedicti Webster, 1879 | species |
| Benthos | Streblospio shrubsolii (Buchanan, 1890) | species |
| Benthos | Strigilla carnaria (Linnaeus, 1758) | species |
| Benthos | Strigilla pisiformis (Linnaeus, 1758) | species |
| Benthos | Syllis gracilis magellanica Augener, 1918 | species |
| Benthos | Syllis Lamarck, 1818 | species |
| Benthos | Tagelus plebeius ([Lightfoot], 1786) | species |
| Benthos | Tectonatica pusilla (Say, 1822) | species |
| Benthos | Thaisella coronata (Lamarck, 1816) | species |
| Benthos | Timarete ceciliae Magalhães, Seixas, Paiva & Elias, 2014 | species |
| Benthos | Uca maracoani (Latreille, 1803) | species |
| Benthos | Ucides cordatus (Linnaeus, 1763) | species |
| Benthos | Vitrinella filifera Pilsbry & T. L. McGinty, 1946 | species |
| Benthos | Vitta virginea (Linnaeus, 1758) | species |
| Benthos | Acari Leach, 1817 | subclass |
| Benthos | Collembola Lubbock, 1870 | subclass |
| Benthos | Insecta Linnaeus, 1758 | Subclass |
| Benthos | Arthropoda von Siebold, 1848 | subphylum |
| Benthos | Crustacea Brünnich, 1772 | subphylum |
| Benthos | Notomastus M. Sars, 1851 | subphylum |
| Benthos | Tanaidacea Dana, 1849 | subphylum |
| Birds | Strigidae | family |
| Birds | Trochilidae | family |
| Birds | Passeriformes | order |
| Birds | Accipiter bicolor (Vieillot, 1817) | species |
| Birds | Actitis macularius (Linnaeus, 1766) | species |
| Birds | Amazona amazonica (Linnaeus, 1766) | species |
| Birds | Amazona farinosa (Boddaert, 1783) | species |
| Birds | Amazonetta brasiliensis (Gmelin, 1789) | species |
| Birds | Ammodramus humeralis (Bosc, 1792) | species |
| Birds | Anhima cornuta  (Linnaeus, 1766) | species |
| Birds | Anhinga anhinga (Linnaeus, 1766) | species |
| Birds | Anous stolidus (Linnaeus, 1758) | species |
| Birds | Anthracothorax nigricollis (Vieillot, 1817) | species |
| Birds | Anthus chii Vieillot, 1818 | species |
| Birds | Antrostomus rufus (Boddaert, 1783) | species |
| Birds | Aramides cajaneus (Statius Muller, 1776) | species |
| Birds | Aramides mangle (Spix, 1825) | species |
| Birds | Aramus guarauna (Linnaeus, 1766) | species |
| Birds | Aratinga jandaya (Gmelin, 1788) | species |
| Birds | Ardea alba Linnaeus, 1758 | species |
| Birds | Ardea cocoi Linnaeus, 1766 | species |
| Birds | Arenaria interpres (Linnaeus, 1758) | species |
| Birds | Arremon taciturnus (Hermann, 1783) | species |
| Birds | Arundinicola leucocephala (Linnaeus, 1764) | species |
| Birds | Asemospiza fuliginosa (Wied, 1830) | species |
| Birds | Asio clamator (Vieillot, 1808) | species |
| Birds | Athene cunicularia (Molina, 1782) | species |
| Birds | Attila cinnamomeus (Gmelin, 1789) | species |
| Birds | Attila spadiceus (Gmelin, 1789) | species |
| Birds | Basileuterus culicivorus (Deppe, 1830) | species |
| Birds | Berlepschia rikeri (Ridgway, 1886) | species |
| Birds | Botaurus pinnatus (Wagler, 1829) | species |
| Birds | Brachygalba lugubris (Swainson, 1838) | species |
| Birds | Brotogeris chiriri (Vieillot, 1818) | species |
| Birds | Brotogeris chrysoptera (Linnaeus, 1766) | species |
| Birds | Bubulcus ibis (Linnaeus, 1758) | species |
| Birds | Buteo albonotatus Kaup, 1847 | species |
| Birds | Buteo brachyurus Vieillot, 1816 | species |
| Birds | Buteo nitidus (Latham, 1790) | species |
| Birds | Buteogallus aequinoctialis (Gmelin, 1788) | species |
| Birds | Butorides striata (Linnaeus, 1758) | species |
| Birds | Cacicus cela (Linnaeus, 1758) | species |
| Birds | Cacicus solitarius (Vieillot, 1816) | species |
| Birds | Cairina moschata (Linnaeus, 1758) | species |
| Birds | Calidris alba (Pallas, 1764) | species |
| Birds | Calidris canutus (Linnaeus, 1758) | species |
| Birds | Calidris fuscicollis (Vieillot, 1819) | species |
| Birds | Calidris mauri (Cabanis, 1857) | species |
| Birds | Calidris melanotos (Vieillot, 1819) | species |
| Birds | Calidris minutilla (Vieillot, 1819) | species |
| Birds | Calidris pusilla (Linnaeus, 1766) | species |
| Birds | Calliphlox amethystina (Boddaert, 1783) | species |
| Birds | Calonectris borealis (Cory, 1881) | species |
| Birds | Campephilus melanoleucos (Gmelin, 1788) | species |
| Birds | Campephilus rubricollis (Boddaert, 1783) | species |
| Birds | Camptostoma obsoletum (Temminck, 1824) | species |
| Birds | Campylorhynchus turdinus (Wied, 1831) | species |
| Birds | Cantorchilus leucotis (Lafresnaye, 1845) | species |
| Birds | Capsiempis flaveola (Lichtenstein, 1823) | species |
| Birds | Caracara plancus (Miller, 1777) | species |
| Birds | Caryothraustes canadensis (Linnaeus, 1766) | species |
| Birds | Casiornis fuscus Sclater & Salvin, 1873 | species |
| Birds | Cathartes aura (Linnaeus, 1758) | species |
| Birds | Cathartes burrovianus Cassin, 1845 | species |
| Birds | Cathartes melambrotus Wetmore, 1964 | species |
| Birds | Celeus elegans (Statius Muller, 1776) | species |
| Birds | Celeus flavescens (Gmelin, 1788) | species |
| Birds | Celeus flavus (Statius Muller, 1776) | species |
| Birds | Celeus ochraceus (Spix, 1824) | species |
| Birds | Cercomacroides laeta (Todd, 1920) | species |
| Birds | Certhiaxis cinnamomeus (Gmelin, 1788) | species |
| Birds | Chaetura brachyura (Jardine, 1846) | species |
| Birds | Chaetura meridionalis Hellmayr, 1907 | species |
| Birds | Chaetura spinicaudus (Temminck, 1839) | species |
| Birds | Charadrius collaris Vieillot, 1818 | species |
| Birds | Charadrius semipalmatus Bonaparte, 1825 | species |
| Birds | Charadrius wilsonia Ord, 1814 | species |
| Birds | Chelidoptera tenebrosa (Pallas, 1782) | species |
| Birds | Chionomesa fimbriata (Gmelin, 1788) | species |
| Birds | Chiroxiphia pareola (Linnaeus, 1766) | species |
| Birds | Chlidonias niger (Linnaeus, 1758) | species |
| Birds | Chlorestes notata (Reich, 1793) | species |
| Birds | Chloroceryle aenea (Pallas, 1764) | species |
| Birds | Chloroceryle amazona (Latham, 1790) | species |
| Birds | Chloroceryle americana (Gmelin, 1788) | species |
| Birds | Chloroceryle inda (Linnaeus, 1766) | species |
| Birds | Chlorostilbon lucidus (Shaw, 1812) | species |
| Birds | Chondrohierax uncinatus (Temminck, 1822) | species |
| Birds | Chordeiles acutipennis (Hermann, 1783) | species |
| Birds | Chordeiles minor (Forster, 1771) | species |
| Birds | Chroicocephalus cirrocephalus (Vieillot, 1818) | species |
| Birds | Chrysolampis mosquitus (Linnaeus, 1758) | species |
| Birds | Chrysomus ruficapillus (Vieillot, 1819) | species |
| Birds | Chrysuronia leucogaster (Gmelin, 1788) | species |
| Birds | Chrysuronia versicolor (Vieillot, 1818) | species |
| Birds | Circus buffoni (Gmelin, 1788) | species |
| Birds | Cissopis leverianus (Gmelin, 1788) | species |
| Birds | Claravis pretiosa (Ferrari-Perez, 1886) | species |
| Birds | Cnemotriccus fuscatus (Wied, 1831) | species |
| Birds | Coccycua minuta (Vieillot, 1817) | species |
| Birds | Coccyzus americanus (Linnaeus, 1758) | species |
| Birds | Coccyzus euleri Cabanis, 1873 | species |
| Birds | Coccyzus melacoryphus Vieillot, 1817 | species |
| Birds | Coccyzus minor (Gmelin, 1788) | species |
| Birds | Cochlearius cochlearius (Linnaeus, 1766) | species |
| Birds | Coereba flaveola (Linnaeus, 1758) | species |
| Birds | Colaptes campestris (Vieillot, 1818) | species |
| Birds | Colaptes melanochloros (Gmelin, 1788) | species |
| Birds | Columba livia Gmelin, 1789 | species |
| Birds | Columbina minuta (Linnaeus, 1766) | species |
| Birds | Columbina passerina (Linnaeus, 1758) | species |
| Birds | Columbina picui (Temminck, 1813) | species |
| Birds | Columbina squammata (Lesson, 1831) | species |
| Birds | Columbina talpacoti (Temminck, 1811) | species |
| Birds | Conirostrum bicolor (Vieillot, 1809) | species |
| Birds | Conirostrum speciosum (Temminck, 1824) | species |
| Birds | Conopophaga roberti Hellmayr, 1905 | species |
| Birds | Coragyps atratus (Bechstein, 1793) | species |
| Birds | Cotinga cotinga (Linnaeus, 1766) | species |
| Birds | Cranioleuca vulpina (Pelzeln, 1856) | species |
| Birds | Crotophaga ani Linnaeus, 1758 | species |
| Birds | Crotophaga major Gmelin, 1788 | species |
| Birds | Crypturellus cinereus (Gmelin, 1789) | species |
| Birds | Crypturellus parvirostris (Wagler, 1827) | species |
| Birds | Crypturellus soui (Hermann, 1783) | species |
| Birds | Crypturellus strigulosus (Temminck, 1815) | species |
| Birds | Crypturellus undulatus (Temminck, 1815) | species |
| Birds | Cyanerpes cyaneus (Linnaeus, 1766) | species |
| Birds | Cyanocorax cyanopogon (Wied, 1821) | species |
| Birds | Cyanoloxia rothschildii (Bartlett, 1890) | species |
| Birds | Cyclarhis gujanensis (Gmelin, 1789) | species |
| Birds | Dacnis cayana (Linnaeus, 1766) | species |
| Birds | Daptrius ater Vieillot, 1816 | species |
| Birds | Dendrocincla fuliginosa (Vieillot, 1818) | species |
| Birds | Dendrocolaptes medius Todd, 1920 | species |
| Birds | Dendrocygna autumnalis (Linnaeus, 1758) | species |
| Birds | Dendrocygna viduata (Linnaeus, 1766) | species |
| Birds | Dendroplex picus (Gmelin, 1788) | species |
| Birds | Diopsittaca nobilis (Linnaeus, 1758) | species |
| Birds | Donacobius atricapilla (Linnaeus, 1766) | species |
| Birds | Dromococcyx pavoninus Pelzeln, 1870 | species |
| Birds | Dromococcyx phasianellus (Spix, 1824) | species |
| Birds | Dryocopus lineatus (Linnaeus, 1766) | species |
| Birds | Egretta caerulea (Linnaeus, 1758) | species |
| Birds | Egretta thula (Molina, 1782) | species |
| Birds | Egretta tricolor (Statius Muller, 1776) | species |
| Birds | Elaenia chiriquensis Lawrence, 1865 | species |
| Birds | Elaenia cristata Pelzeln, 1868 | species |
| Birds | Elaenia flavogaster (Thunberg, 1822) | species |
| Birds | Elaenia spectabilis Pelzeln, 1868 | species |
| Birds | Elanoides forficatus (Linnaeus, 1758) | species |
| Birds | Elanus leucurus (Vieillot, 1818) | species |
| Birds | Emberizoides herbicola (Vieillot, 1817) | species |
| Birds | Empidonomus varius (Vieillot, 1818) | species |
| Birds | Estrilda astrild (Linnaeus, 1758) | species |
| Birds | Eucometis penicillata (Spix, 1825) | species |
| Birds | Eudocimus ruber (Linnaeus, 1758) | species |
| Birds | Eupetomena macroura (Gmelin, 1788) | species |
| Birds | Euphonia chlorotica (Linnaeus, 1766) | species |
| Birds | Euphonia violacea (Linnaeus, 1758) | species |
| Birds | Eupsittula aurea (Gmelin, 1788) | species |
| Birds | Eurypyga helias (Pallas, 1781) | species |
| Birds | Euscarthmus meloryphus Wied, 1831 | species |
| Birds | Falco femoralis Temminck, 1822 | species |
| Birds | Falco peregrinus Tunstall, 1771 | species |
| Birds | Falco rufigularis Daudin, 1800 | species |
| Birds | Falco sparverius Linnaeus, 1758 | species |
| Birds | Florisuga mellivora (Linnaeus, 1758) | species |
| Birds | Fluvicola albiventer (Spix, 1825) | species |
| Birds | Fluvicola nengeta (Linnaeus, 1766) | species |
| Birds | Formicarius analis (d'Orbigny & Lafresnaye, 1837) | species |
| Birds | Formicarius colma Boddaert, 1783 | species |
| Birds | Formicivora grisea (Boddaert, 1783) | species |
| Birds | Formicivora rufa (Wied, 1831) | species |
| Birds | Forpus xanthopterygius (Spix, 1824) | species |
| Birds | Fregata magnificens Mathews, 1914 | species |
| Birds | Furnarius figulus (Lichtenstein, 1823) | species |
| Birds | Furnarius leucopus Swainson, 1838 | species |
| Birds | Galbula ruficauda Cuvier, 1816 | species |
| Birds | Gallinago paraguaiae (Vieillot, 1816) | species |
| Birds | Gallinula galeata (Lichtenstein, 1818) | species |
| Birds | Gampsonyx swainsonii Vigors, 1825 | species |
| Birds | Gelochelidon nilotica (Gmelin, 1789) | species |
| Birds | Geothlypis aequinoctialis (Gmelin, 1789) | species |
| Birds | Geranoaetus albicaudatus (Vieillot, 1816) | species |
| Birds | Geranospiza caerulescens (Vieillot, 1817) | species |
| Birds | Glaucidium brasilianum (Gmelin, 1788) | species |
| Birds | Glaucis hirsutus (Gmelin, 1788) | species |
| Birds | Glyphorynchus spirurus (Vieillot, 1819) | species |
| Birds | Gnorimopsar chopi (Vieillot, 1819) | species |
| Birds | Griseotyrannus aurantioatrocristatus (d'Orbigny & Lafresnaye, 1837) | species |
| Birds | Guira guira (Gmelin, 1788) | species |
| Birds | Haematopus palliatus Temminck, 1820 | species |
| Birds | Harpagus diodon (Temminck, 1823) | species |
| Birds | Helicolestes hamatus (Temminck, 1821) | species |
| Birds | Heliomaster longirostris (Audebert & Vieillot, 1801) | species |
| Birds | Heliornis fulica (Boddaert, 1783) | species |
| Birds | Hemithraupis guira (Linnaeus, 1766) | species |
| Birds | Hemitriccus margaritaceiventer (d'Orbigny & Lafresnaye, 1837) | species |
| Birds | Hemitriccus striaticollis (Lafresnaye, 1853) | species |
| Birds | Herpetotheres cachinnans (Linnaeus, 1758) | species |
| Birds | Herpsilochmus atricapillus Pelzeln, 1868 | species |
| Birds | Herpsilochmus pectoralis Sclater, 1857 | species |
| Birds | Heterospizias meridionalis (Latham, 1790) | species |
| Birds | Himantopus mexicanus (Statius Muller, 1776) | species |
| Birds | Hirundinea ferruginea (Gmelin, 1788) | species |
| Birds | Hirundo rustica Linnaeus, 1758 | species |
| Birds | Hydrobates leucorhous (Vieillot, 1818) | species |
| Birds | Hydropsalis maculicaudus (Lawrence, 1862) | species |
| Birds | Hydropsalis parvula (Gould, 1837) | species |
| Birds | Hydropsalis torquata (Gmelin, 1789) | species |
| Birds | Hylophilus pectoralis Sclater, 1866 | species |
| Birds | Hypocnemoides maculicauda (Pelzeln, 1868) | species |
| Birds | Icterus jamacaii (Gmelin, 1788) | species |
| Birds | Ictinia plumbea (Gmelin, 1788) | species |
| Birds | Ixobrychus exilis (Gmelin, 1789) | species |
| Birds | Ixobrychus involucris (Vieillot, 1823) | species |
| Birds | Jabiru mycteria (Lichtenstein, 1819) | species |
| Birds | Jacana jacana (Linnaeus, 1766) | species |
| Birds | Larus dominicanus Lichtenstein, 1823 | species |
| Birds | Larus fuscus Linnaeus, 1758 | species |
| Birds | Laterallus exilis (Temminck, 1831) | species |
| Birds | Laterallus flaviventer (Boddaert, 1783) | species |
| Birds | Laterallus jamaicensis (Gmelin, 1789) | species |
| Birds | Laterallus melanophaius (Vieillot, 1819) | species |
| Birds | Lathrotriccus euleri (Cabanis, 1868) | species |
| Birds | Legatus leucophaius (Vieillot, 1818) | species |
| Birds | Leistes militaris (Linnaeus, 1758) | species |
| Birds | Leistes superciliaris (Bonaparte, 1850) | species |
| Birds | Lepidocolaptes angustirostris (Vieillot, 1818) | species |
| Birds | Leptodon cayanensis (Latham, 1790) | species |
| Birds | Leptopogon amaurocephalus Tschudi, 1846 | species |
| Birds | Leptotila rufaxilla (Richard & Bernard, 1792) | species |
| Birds | Leptotila verreauxi Bonaparte, 1855 | species |
| Birds | Leucophaeus atricilla (Linnaeus, 1758) | species |
| Birds | Leucophaeus pipixcan (Wagler, 1831) | species |
| Birds | Limnodromus griseus (Gmelin, 1789) | species |
| Birds | Lophotriccus galeatus (Boddaert, 1783) | species |
| Birds | Loriotus luctuosus (d'Orbigny & Lafresnaye, 1837) | species |
| Birds | Lurocalis semitorquatus (Gmelin, 1789) | species |
| Birds | Machetornis rixosa (Vieillot, 1819) | species |
| Birds | Malacoptila minor Sassi, 1911 | species |
| Birds | Manacus manacus (Linnaeus, 1766) | species |
| Birds | Megaceryle torquata (Linnaeus, 1766) | species |
| Birds | Megarynchus pitangua (Linnaeus, 1766) | species |
| Birds | Megascops choliba (Vieillot, 1817) | species |
| Birds | Melanerpes candidus (Otto, 1796) | species |
| Birds | Mesembrinibis cayennensis (Gmelin, 1789) | species |
| Birds | Micrastur ruficollis (Vieillot, 1817) | species |
| Birds | Micrastur semitorquatus (Vieillot, 1817) | species |
| Birds | Milvago chimachima (Vieillot, 1816) | species |
| Birds | Mimus gilvus (Vieillot, 1807) | species |
| Birds | Mimus saturninus (Lichtenstein, 1823) | species |
| Birds | Mionectes oleagineus (Lichtenstein, 1823) | species |
| Birds | Molothrus bonariensis (Gmelin, 1789) | species |
| Birds | Molothrus oryzivorus (Gmelin, 1788) | species |
| Birds | Momotus momota (Linnaeus, 1766) | species |
| Birds | Monasa nigrifrons (Spix, 1824) | species |
| Birds | Mustelirallus albicollis (Vieillot, 1819) | species |
| Birds | Mycteria americana Linnaeus, 1758 | species |
| Birds | Myiarchus ferox (Gmelin, 1789) | species |
| Birds | Myiarchus swainsoni Cabanis & Heine, 1859 | species |
| Birds | Myiarchus tyrannulus (Statius Muller, 1776) | species |
| Birds | Myiodynastes maculatus (Statius Muller, 1776) | species |
| Birds | Myiopagis caniceps (Swainson, 1835) | species |
| Birds | Myiopagis gaimardii (d'Orbigny, 1839) | species |
| Birds | Myiopagis viridicata (Vieillot, 1817) | species |
| Birds | Myiophobus fasciatus (Statius Muller, 1776) | species |
| Birds | Myiothlypis flaveola Baird, 1865 | species |
| Birds | Myiozetetes cayanensis (Linnaeus, 1766) | species |
| Birds | Myiozetetes similis (Spix, 1825) | species |
| Birds | Myrmotherula axillaris (Vieillot, 1817) | species |
| Birds | Nannopterum brasilianum (Gmelin, 1789) | species |
| Birds | Nasica longirostris (Vieillot, 1818) | species |
| Birds | Nemosia pileata (Boddaert, 1783) | species |
| Birds | Nengetus cinereus (Vieillot, 1816) | species |
| Birds | Neocrex erythrops (Sclater, 1867) | species |
| Birds | Neopelma pallescens (Lafresnaye, 1853) | species |
| Birds | Nomonyx dominicus (Linnaeus, 1766) | species |
| Birds | Notharchus tectus (Boddaert, 1783) | species |
| Birds | Nothura boraquira (Spix, 1825) | species |
| Birds | Numenius hudsonicus Latham, 1790 | species |
| Birds | Nyctanassa violacea (Linnaeus, 1758) | species |
| Birds | Nyctibius griseus (Gmelin, 1789) | species |
| Birds | Nycticorax nycticorax (Linnaeus, 1758) | species |
| Birds | Nyctidromus albicollis (Gmelin, 1789) | species |
| Birds | Nyctidromus nigrescens (Cabanis, 1849) | species |
| Birds | Nyctiprogne leucopyga (Spix, 1825) | species |
| Birds | Nystalus maculatus (Gmelin, 1788) | species |
| Birds | Onychoprion fuscatus (Linnaeus, 1766) | species |
| Birds | Opisthocomus hoazin (Statius Muller, 1776) | species |
| Birds | Ornithion inerme Hartlaub, 1853 | species |
| Birds | Ortalis superciliaris (Gray, 1867) | species |
| Birds | Orthopsittaca manilatus (Boddaert, 1783) | species |
| Birds | Pachyramphus marginatus (Lichtenstein, 1823) | species |
| Birds | Pachyramphus polychopterus (Vieillot, 1818) | species |
| Birds | Pachyramphus rufus (Boddaert, 1783) | species |
| Birds | Pachyramphus validus (Lichtenstein, 1823) | species |
| Birds | Pandion haliaetus (Linnaeus, 1758) | species |
| Birds | Panyptila cayennensis (Gmelin, 1789) | species |
| Birds | Pardirallus maculatus (Boddaert, 1783) | species |
| Birds | Paroaria dominicana (Linnaeus, 1758) | species |
| Birds | Passer domesticus (Linnaeus, 1758) | species |
| Birds | Patagioenas cayennensis (Bonnaterre, 1792) | species |
| Birds | Patagioenas picazuro (Temminck, 1813) | species |
| Birds | Patagioenas speciosa (Gmelin, 1789) | species |
| Birds | Penelope superciliaris Temminck, 1815 | species |
| Birds | Phaeomyias murina (Spix, 1825) | species |
| Birds | Phaethornis maranhaoensis Grantsau, 1968 | species |
| Birds | Phaethornis pretrei (Lesson & Delattre, 1839) | species |
| Birds | Phaethornis ruber (Linnaeus, 1758) | species |
| Birds | Phaetusa simplex (Gmelin, 1789) | species |
| Birds | Pheugopedius genibarbis (Swainson, 1838) | species |
| Birds | Philohydor lictor (Lichtenstein, 1823) | species |
| Birds | Phimosus infuscatus (Lichtenstein, 1823) | species |
| Birds | Piaya cayana (Linnaeus, 1766) | species |
| Birds | Picumnus pygmaeus (Lichtenstein, 1823) | species |
| Birds | Pilherodius pileatus (Boddaert, 1783) | species |
| Birds | Pionus menstruus (Linnaeus, 1766) | species |
| Birds | Piranga flava (Vieillot, 1822) | species |
| Birds | Pitangus sulphuratus (Linnaeus, 1766) | species |
| Birds | Platalea ajaja Linnaeus, 1758 | species |
| Birds | Platyrinchus mystaceus Vieillot, 1818 | species |
| Birds | Pluvialis dominica (Statius Muller, 1776) | species |
| Birds | Pluvialis squatarola (Linnaeus, 1758) | species |
| Birds | Podager nacunda (Vieillot, 1817) | species |
| Birds | Podilymbus podiceps (Linnaeus, 1758) | species |
| Birds | Poecilotriccus fumifrons (Hartlaub, 1853) | species |
| Birds | Poecilotriccus sylvia (Desmarest, 1806) | species |
| Birds | Polioptila atricapilla (Swainson, 1831) | species |
| Birds | Polioptila plumbea (Gmelin, 1788) | species |
| Birds | Polytmus guainumbi (Pallas, 1764) | species |
| Birds | Porphyrio flavirostris (Gmelin, 1789) | species |
| Birds | Porphyrio martinica (Linnaeus, 1766) | species |
| Birds | Primolius maracana (Vieillot, 1816) | species |
| Birds | Procnias averano (Hermann, 1783) | species |
| Birds | Progne chalybea (Gmelin, 1789) | species |
| Birds | Progne subis (Linnaeus, 1758) | species |
| Birds | Progne tapera (Linnaeus, 1766) | species |
| Birds | Psarocolius decumanus (Pallas, 1769) | species |
| Birds | Pseudastur albicollis (Latham, 1790) | species |
| Birds | Pseudocolopteryx sclateri (Oustalet, 1892) | species |
| Birds | Psittacara leucophthalmus (Statius Muller, 1776) | species |
| Birds | Pteroglossus aracari (Linnaeus, 1758) | species |
| Birds | Pteroglossus bitorquatus Vigors, 1826 | species |
| Birds | Pteroglossus inscriptus Swainson, 1822 | species |
| Birds | Pulsatrix perspicillata (Latham, 1790) | species |
| Birds | Pygochelidon cyanoleuca (Vieillot, 1817) | species |
| Birds | Pygochelidon melanoleuca (Wied, 1820) | species |
| Birds | Pyriglena leuconota (Spix, 1824) | species |
| Birds | Pyrrhura coerulescens Neumann, 1927 | species |
| Birds | Rallus longirostris Boddaert, 1783 | species |
| Birds | Ramphastos tucanus Linnaeus, 1758 | species |
| Birds | Ramphastos vitellinus Lichtenstein, 1823 | species |
| Birds | Ramphocaenus melanurus Vieillot, 1819 | species |
| Birds | Ramphocelus carbo (Pallas, 1764) | species |
| Birds | Rostrhamus sociabilis (Vieillot, 1817) | species |
| Birds | Rufirallus viridis (Statius Muller, 1776) | species |
| Birds | Rupornis magnirostris (Gmelin, 1788) | species |
| Birds | Rynchops niger Linnaeus, 1758 | species |
| Birds | Sakesphorus luctuosus (Lichtenstein, 1823) | species |
| Birds | Saltator coerulescens Vieillot, 1817 | species |
| Birds | Saltator maximus (Statius Muller, 1776) | species |
| Birds | Sarkidiornis sylvicola Ihering & Ihering, 1907 | species |
| Birds | Satrapa icterophrys (Vieillot, 1818) | species |
| Birds | Schistochlamys melanopis (Latham, 1790) | species |
| Birds | Sclateria naevia (Gmelin, 1788) | species |
| Birds | Setophaga pitiayumi (Vieillot, 1817) | species |
| Birds | Sicalis columbiana Cabanis, 1851 | species |
| Birds | Sicalis flaveola (Linnaeus, 1766) | species |
| Birds | Sittasomus griseicapillus (Vieillot, 1818) | species |
| Birds | Spatula discors (Linnaeus, 1766) | species |
| Birds | Spizaetus tyrannus (Wied, 1820) | species |
| Birds | Sporophila americana (Gmelin, 1789) | species |
| Birds | Sporophila angolensis (Linnaeus, 1766) | species |
| Birds | Sporophila bouvreuil (Statius Muller, 1776) | species |
| Birds | Sporophila caerulescens (Vieillot, 1823) | species |
| Birds | Sporophila collaris (Boddaert, 1783) | species |
| Birds | Sporophila leucoptera (Vieillot, 1817) | species |
| Birds | Sporophila lineola (Linnaeus, 1758) | species |
| Birds | Sporophila minuta (Linnaeus, 1758) | species |
| Birds | Sporophila nigricollis (Vieillot, 1823) | species |
| Birds | Sporophila ruficollis Cabanis, 1851 | species |
| Birds | Stelgidopteryx ruficollis (Vieillot, 1817) | species |
| Birds | Stercorarius parasiticus (Linnaeus, 1758) | species |
| Birds | Stercorarius pomarinus (Temminck, 1815) | species |
| Birds | Sterna dougallii Montagu, 1813 | species |
| Birds | Sterna hirundo Linnaeus, 1758 | species |
| Birds | Sterna paradisaea Pontoppidan, 1763 | species |
| Birds | Sternula antillarum Lesson, 1847 | species |
| Birds | Sternula superciliaris (Vieillot, 1819) | species |
| Birds | Stilpnia cayana (Linnaeus, 1766) | species |
| Birds | Streptoprocne zonaris (Shaw, 1796) | species |
| Birds | Strix huhula Daudin, 1800 | species |
| Birds | Suiriri suiriri (Vieillot, 1818) | species |
| Birds | Sula sula (Linnaeus, 1766) | species |
| Birds | Synallaxis albescens Temminck, 1823 | species |
| Birds | Synallaxis frontalis Pelzeln, 1859 | species |
| Birds | Synallaxis gujanensis (Gmelin, 1789) | species |
| Birds | Synallaxis rutilans Temminck, 1823 | species |
| Birds | Synallaxis sp. | species |
| Birds | Tachornis squamata (Cassin, 1853) | species |
| Birds | Tachybaptus dominicus (Linnaeus, 1766) | species |
| Birds | Tachycineta albiventer (Boddaert, 1783) | species |
| Birds | Tachyphonus rufus (Boddaert, 1783) | species |
| Birds | Taeniotriccus andrei (Berlepsch & Hartert, 1902) | species |
| Birds | Tapera naevia (Linnaeus, 1766) | species |
| Birds | Taraba major (Vieillot, 1816) | species |
| Birds | Thalasseus acuflavidus (Cabot, 1847) | species |
| Birds | Thalasseus maximus (Boddaert, 1783) | species |
| Birds | Thalurania furcata (Gmelin, 1788) | species |
| Birds | Thamnophilus amazonicus Sclater, 1858 | species |
| Birds | Thamnophilus doliatus (Linnaeus, 1764) | species |
| Birds | Thamnophilus palliatus (Lichtenstein, 1823) | species |
| Birds | Thamnophilus pelzelni Hellmayr, 1924 | species |
| Birds | Thlypopsis sordida (d'Orbigny & Lafresnaye, 1837) | species |
| Birds | Thraupis episcopus (Linnaeus, 1766) | species |
| Birds | Thraupis palmarum (Wied, 1821) | species |
| Birds | Thraupis sayaca (Linnaeus, 1766) | species |
| Birds | Tigrisoma lineatum (Boddaert, 1783) | species |
| Birds | Tityra inquisitor (Lichtenstein, 1823) | species |
| Birds | Tityra semifasciata (Spix, 1825) | species |
| Birds | Todirostrum cinereum (Linnaeus, 1766) | species |
| Birds | Todirostrum maculatum (Desmarest, 1806) | species |
| Birds | Tolmomyias flaviventris (Wied, 1831) | species |
| Birds | Tolmomyias poliocephalus (Taczanowski, 1884) | species |
| Birds | Tolmomyias sulphurescens (Spix, 1825) | species |
| Birds | Tringa flavipes (Gmelin, 1789) | species |
| Birds | Tringa inornata (Brewster, 1887) | species |
| Birds | Tringa melanoleuca (Gmelin, 1789) | species |
| Birds | Tringa semipalmata (Gmelin, 1789) | species |
| Birds | Tringa solitaria Wilson, 1813 | species |
| Birds | Troglodytes musculus Naumann, 1823 | species |
| Birds | Trogon curucui Linnaeus, 1766 | species |
| Birds | Trogon viridis Linnaeus, 1766 | species |
| Birds | Turdus amaurochalinus Cabanis, 1850 | species |
| Birds | Turdus fumigatus Lichtenstein, 1823 | species |
| Birds | Turdus leucomelas Vieillot, 1818 | species |
| Birds | Turdus nudigenis Lafresnaye, 1848 | species |
| Birds | Tyrannopsis sulphurea (Spix, 1825) | species |
| Birds | Tyrannulus elatus (Latham, 1790) | species |
| Birds | Tyrannus albogularis Burmeister, 1856 | species |
| Birds | Tyrannus melancholicus Vieillot, 1819 | species |
| Birds | Tyrannus savana Daudin, 1802 | species |
| Birds | Tyto furcata (Temminck, 1827) | species |
| Birds | Uropelia campestris (Spix, 1825) | species |
| Birds | Urubitinga urubitinga (Gmelin, 1788) | species |
| Birds | Vanellus cayanus (Latham, 1790) | species |
| Birds | Vanellus chilensis (Molina, 1782) | species |
| Birds | Veniliornis affinis (Swainson, 1821) | species |
| Birds | Veniliornis passerinus (Linnaeus, 1766) | species |
| Birds | Vireo chivi (Vieillot, 1817) | species |
| Birds | Volatinia jacarina (Linnaeus, 1766) | species |
| Birds | Xema sabini (Sabine, 1819) | species |
| Birds | Xenops minutus (Sparrman, 1788) | species |
| Birds | Xenopsaris albinucha (Burmeister, 1869) | species |
| Birds | Xiphorhynchus guttatoides (Lafresnaye, 1850) | species |
| Birds | Zebrilus undulatus (Gmelin, 1789) | species |
| Birds | Zenaida auriculata (Des Murs, 1847) | species |
| Birds | Zimmerius acer (Salvin & Godman, 1883) | species |
| Birds | Zonotrichia capensis (Statius Muller, 1776) | species |
| Fishes | Ariidae | family |
| Fishes | Carangidae | family |
| Fishes | Clupeidae | family |
| Fishes | Curimatidae | family |
| Fishes | Enchodontidae | family |
| Fishes | Semionotidae | family |
| Fishes | Sparidae | family |
| Fishes | Acestrorhynchus cf. falcatus (Bloch 1794) | genus |
| Fishes | Achirus sp. | genus |
| Fishes | Ageneiosus sp. | genus |
| Fishes | Amphichthys sp. | genus |
| Fishes | Anchoa sp. | genus |
| Fishes | Ancistrus sp. | genus |
| Fishes | Atherinichthys sp. | genus |
| Fishes | Auchenipterichthys sp. | genus |
| Fishes | Auchenipterus sp | genus |
| Fishes | Cathorops sp. | genus |
| Fishes | Chaetodipterus sp. | genus |
| Fishes | Cichlasoma zarskei Ottoni 2011 | genus |
| Fishes | Citharichthys sp. | genus |
| Fishes | Crenicichla sp. | genus |
| Fishes | Eigenmmania sp | genus |
| Fishes | Elops sp. | genus |
| Fishes | Eucinostomus sp. | genus |
| Fishes | Farlowella sp. | genus |
| Fishes | Geophagus sp | genus |
| Fishes | Gobioides sp. | genus |
| Fishes | Gymnotus sp. | genus |
| Fishes | Haemulon sp. | genus |
| Fishes | Hassar sp. | genus |
| Fishes | Hemiodus sp | genus |
| Fishes | Hoplias sp. | genus |
| Fishes | Hypostomus sp. | genus |
| Fishes | Lepidotes sp. | genus |
| Fishes | Leporinus sp | genus |
| Fishes | Loricaria sp. | genus |
| Fishes | Loricariichthys sp | genus |
| Fishes | Lycengraulis sp. | genus |
| Fishes | Metynnis sp | genus |
| Fishes | Micropoecilia sp. | genus |
| Fishes | Moenkhausia sp. | genus |
| Fishes | Mugil sp. | genus |
| Fishes | Notarius sp. | genus |
| Fishes | Parauchenipterus sp. | genus |
| Fishes | *Pellona harroweri* (Fowler 1917) | genus |
| Fishes | Pellona sp. | genus |
| Fishes | Pimelodella sp | genus |
| Fishes | Pimelodus sp. | genus |
| Fishes | Poecilia sp. | genus |
| Fishes | Pristipoma sp. | genus |
| Fishes | Pseudobunocephalus sp | genus |
| Fishes | Pseudopimelodus sp | genus |
| Fishes | Pterengraulis sp | genus |
| Fishes | Pterygoplichthys sp | genus |
| Fishes | Rhamdia sp. | genus |
| Fishes | Rhinosardinia sp. | genus |
| Fishes | Roeboides sp | genus |
| Fishes | Rypticus sp. | genus |
| Fishes | Satanoperca sp. | genus |
| Fishes | Scarus sp. | genus |
| Fishes | Schizodon sp. | genus |
| Fishes | Sciaena sp. | genus |
| Fishes | Serrapinnus sp | genus |
| Fishes | Serrasalmus sp | genus |
| Fishes | Sphoeroides sp | genus |
| Fishes | Sphyraena sp. | genus |
| Fishes | Stellifer sp. | genus |
| Fishes | Sternopygus sp. | genus |
| Fishes | Sturisoma sp. | genus |
| Fishes | Symphurus sp. | genus |
| Fishes | Trachelyopterus sp. | genus |
| Fishes | Trichopsetta sp. | genus |
| Fishes | Triportheus signatus (Garman 1890) | genus |
| Fishes | Siluriformes | order |
| Fishes | *Acanthostracion polygonium* Poey 1876 | species |
| Fishes | *Acanthurus coeruleus* Bloch & Schneider 1801 | species |
| Fishes | Acestrorhynchus cf. falcatus (Bloch 1794) | species |
| Fishes | Acestrorhynchus lacustris (Lutken 1875) | species |
| Fishes | Achirus achirus (Linnaeus 1758) | species |
| Fishes | Achirus lineatus (Linnaeus 1758) | species |
| Fishes | Aequidens tetramerus (Heckel 1840) | species |
| Fishes | Ageneiosus dentatus Kner 1858 | species |
| Fishes | Ageneiosus ucayalensis Castelnau 1855 | species |
| Fishes | Albula vulpes (Linnaeus 1758) | species |
| Fishes | *Aluterus monoceros* (Linnaeus 1758) | species |
| Fishes | *Amphiarius phrygiatus* (Valenciennes 1840) | species |
| Fishes | Amphiarius rugispinis (Valenciennes 1840) | species |
| Fishes | Anableps anableps (Linnaeus 1758) | species |
| Fishes | Anablepsoides urophthalmus (Günther 1866) | species |
| Fishes | Anchoa spinifer (Valenciennes 1848) | species |
| Fishes | *Anchovia clupeoides* (Swainson 1839) | species |
| Fishes | Anchovia surinamensis (Bleeker 1865) | species |
| Fishes | Anchoviella elongata (Meek & Hildebrand, 1923) | species |
| Fishes | Anchoviella lepidentostole (Fowler 1911) | species |
| Fishes | *Apionichthys dumerili* Kaup 1858 | species |
| Fishes | Apteronotus albifrons (Linnaeus 1766) | species |
| Fishes | *Archosargus probatocephalus* (Walbaum 1792) | species |
| Fishes | Aspistor luniscutis (Valenciennes 1840) | species |
| Fishes | Aspistor quadriscutis (Valenciennes 1840) | species |
| Fishes | Aspredinichthys filamentosus (Valenciennes, 1840) | species |
| Fishes | Aspredinichthys tibicen (Valenciennes 1840) | species |
| Fishes | Aspredo aspredo (Linnaeus 1758) | species |
| Fishes | Astyanax bimaculatus (Linnaeus 1758) | species |
| Fishes | Astyanax cf. bimaculatus (Linnaeus 1758) | species |
| Fishes | Atherinella brasiliensis (Quoy & Gaimard 1825) | species |
| Fishes | Auchenipterus menezesi Ferraris & Vari 1999 | species |
| Fishes | Auchenipterus nuchalis (Spix & Agassiz 1829) | species |
| Fishes | Bagre bagre (Linnaeus 1766) | species |
| Fishes | Bagre marinus (Mitchill, 1815) | species |
| Fishes | Bairdiella ronchus (Cuvier 1830) | species |
| Fishes | *Bathygobius soporator* (Valenciennes 1837) | species |
| Fishes | Batrachoides surinamensis (Bloch & Schneider 1801) | species |
| Fishes | Brachychalcinus orbicularis (Valenciennes 1850) | species |
| Fishes | Brachyhypopomus pinnicaudatus (Hopkins, Comfort, Bastian & Bass 1990) | species |
| Fishes | Callichthys callichthys (Linnaeus 1758) | species |
| Fishes | *Canthigaster figueiredoi* Moura & Castro 2002 | species |
| Fishes | *Canthigaster punctatissima* (Günther 1870) | species |
| Fishes | Caranx crysos (Mitchill 1815) | species |
| Fishes | Caranx hippos (Linnaeus 1766) | species |
| Fishes | Caranx latus Agassiz 1831 | species |
| Fishes | *Cathorops agassizii* (Eigenmann & Eigenmann 1888) | species |
| Fishes | *Cathorops arenatus* (Valenciennes 1840) | species |
| Fishes | Cathorops spixii (Agassiz 1829) | species |
| Fishes | Centropomus parallelus Poey 1860 | species |
| Fishes | Centropomus undecimalis (Bloch 1792) | species |
| Fishes | *Cephalopholis fulva* (Linnaeus 1758) | species |
| Fishes | Cetengraulis edentulus (Cuvier 1829) | species |
| Fishes | Chaetodipterus faber (Broussonet 1782) | species |
| Fishes | *Chaetodon striatus* Linnaeus 1758 | species |
| Fishes | Charax awa Guimarães, Brito, Ferreira & Ottoni 2018 | species |
| Fishes | Chilomycterus antillarum Jordan & Rutter,1897 | species |
| Fishes | Chloroscombrus chrysurus (Linnaeus 1766) | species |
| Fishes | Cichlasoma zarskei Ottoni 2011 | species |
| Fishes | *Citharichthys arenaceus* Evermann & Marsh 1900 | species |
| Fishes | Citharichthys spilopterus Günther 1862 | species |
| Fishes | Colomesus psittacus (Bloch & Schneider 1801) | species |
| Fishes | Colossoma macropomum (Cuvier 1816) | species |
| Fishes | *Conodon nobilis* (Linnaeus 1758) | species |
| Fishes | Crenicichla brasiliensis (Bloch 1792) | species |
| Fishes | Crenicichla sp. | species |
| Fishes | Ctenobrycon cf. hauxwellianus | species |
| Fishes | Ctenobrycon cf. spilurus | species |
| Fishes | Ctenobrycon hauxwellianus (Cope 1870) | species |
| Fishes | Ctenogobius smaragdus (Valenciennes 1837) | species |
| Fishes | *Ctenogobius stigmaticus* (Poey 1860) | species |
| Fishes | Curimata cf. roseni Vari, 1989 | species |
| Fishes | Curimata cyprinoides (Linnaeus 1766) | species |
| Fishes | Curimata macrops Eigenmann & Eigenmann 1889 | species |
| Fishes | Curimatella dorsalis (Eigenmann & Eigenmann 1889) | species |
| Fishes | Cynodon gibbus (Agassiz 1829) | species |
| Fishes | Cynoscion acoupa (Lacepède 1801) | species |
| Fishes | Cynoscion guatucupa (Cuvier 1830) | species |
| Fishes | Cynoscion jamaicensis (Vaillant & Bocourt 1883) | species |
| Fishes | Cynoscion leiarchus (Cuvier 1830) | species |
| Fishes | Cynoscion microlepidotus (Cuvier 1830) | species |
| Fishes | Cynoscion steindachneri (Jordan 1889) | species |
| Fishes | Cynoscion virescens (Cuvier, 1830) | species |
| Fishes | Cyphocharax voga (Hensel 1870) | species |
| Fishes | *Diapterus auratus* Ranzani 1842 | species |
| Fishes | Diapterus rhombeus (Cuvier 1829) | species |
| Fishes | *Diplectrum radiale* (Quoy & Gaimard 1824) | species |
| Fishes | *Echeneis naucrates* Linnaeus 1758 | species |
| Fishes | Electrophorus electricus (Linnaeus 1766) | species |
| Fishes | *Eleotris pisonis* (Gmelin 1789) | species |
| Fishes | Elops saurus Linnaeus 1766 | species |
| Fishes | Elops smithi McBride, Rocha, Ruiz-Carus & Bowen 2010 | species |
| Fishes | *Epinephelus adscensionis* (Osbeck 1765) | species |
| Fishes | Epinephelus itajara (Lichtenstein 1822) | species |
| Fishes | *Etropus crossotus* Jordan & Gilbert 1882 | species |
| Fishes | *Eucinostomus argenteus* Baird & Girard 1855 | species |
| Fishes | Eucinostomus gula (Quoy & Gaimard 1824) | species |
| Fishes | *Eucinostomus melanopterus* (Bleeker 1863) | species |
| Fishes | Eugerres brasilianus (Cuvier 1830) | species |
| Fishes | Eugerres plumieri (Cuvier, 1830) | species |
| Fishes | *Evorthodus lyricus* (Girard 1858) | species |
| Fishes | Fontitrygon geijskesi (Boeseman 1948) | species |
| Fishes | Gasteropelecus sternicla (Linnaeus 1758) | species |
| Fishes | Genidens barbus (Lacepède, 1803) | species |
| Fishes | *Genyatremus cavifrons* (Cuvier 1830) | species |
| Fishes | Genyatremus luteus (Bloch 1790) | species |
| Fishes | Geophagus parnaibae Staeck & Schindler 2006 | species |
| Fishes | Geophagus surinamensis (Bloch 1791) | species |
| Fishes | Gobioides broussonnetii Lacepède, 1800 | species |
| Fishes | Gobionellus oceanicus (Pallas, 1770) | species |
| Fishes | *Guavina guavina* (Valenciennes 1837) | species |
| Fishes | Gymnothorax funebris Ranzani 1839 | species |
| Fishes | Gymnotus carapo Linnaeus 1758 | species |
| Fishes | Gymnura micrura (Bloch & Schneider 1801) | species |
| Fishes | Haemulon aurolineatum Cuvier, 1830 | species |
| Fishes | Haemulon steindachneri (Jordan & Gilbert 1882) | species |
| Fishes | Haemulopsis corvinaeformis (Steindachner 1868) | species |
| Fishes | Halichoeres radiatus (Linnaeus 1758) | species |
| Fishes | Hassar affinis (Steindachner 1881) | species |
| Fishes | Hassar wilderi Kindle 1895 | species |
| Fishes | *Hemicaranx amblyrhynchus* (Cuvier 1833) | species |
| Fishes | *Hemigrammus guyanensis* Géry 1959 | species |
| Fishes | Hemigrammus ocellifer (Steindachner 1882) | species |
| Fishes | Hemigrammus rodwayi Durbin 1909 | species |
| Fishes | *Hemiramphus brasiliensis* (Linnaeus 1758) | species |
| Fishes | Hemisorubim platyrhynchos (Valenciennes 1840) | species |
| Fishes | Hippocampus reidi Ginsburg, 1933 | species |
| Fishes | *Holacanthus ciliaris* (Linnaeus 1758) | species |
| Fishes | Hoplerythrinus unitaeniatus (Spix & Agassiz 1829) | species |
| Fishes | Hoplias malabaricus (Bloch 1794) | species |
| Fishes | Hypanus guttatus (Bloch & Schneider 1801) | species |
| Fishes | Hypoptopoma incognitum Aquino & Schaefer 2010 | species |
| Fishes | Hypostomus plecostomus (Linnaeus 1758) | species |
| Fishes | Hypostomus watwata Hancock 1828 | species |
| Fishes | Isogomphodon oxyrhynchus (Müller & Henle 1839) | species |
| Fishes | Isopisthus parvipinnis (Cuvier 1830) | species |
| Fishes | Lagocephalus laevigatus (Linnaeus, 1766) | species |
| Fishes | *Larimus breviceps* Cuvier 1830 | species |
| Fishes | Leporinus friderici (Bloch 1794) | species |
| Fishes | Leporinus piau Fowler 1941 | species |
| Fishes | Lile piquitinga (Schreiner & Miranda Ribeiro 1903) | species |
| Fishes | Lobotes surinamensis (Bloch 1790) | species |
| Fishes | *Lonchurus lanceolatus* (Bloch 1788) | species |
| Fishes | Loricaria cataphracta Linnaeus 1758 | species |
| Fishes | Loricariichthys derbyi Fowler 1915 | species |
| Fishes | Loricariichthys nudirostris (Kner 1853) | species |
| Fishes | Lutjanidae | species |
| Fishes | *Lutjanus alexandrei* Moura & Lindeman 2007 | species |
| Fishes | *Lutjanus cyanopterus* (Cuvier 1828) | species |
| Fishes | Lutjanus jocu (Bloch & Schneider 1801) | species |
| Fishes | *Lutjanus synagris* (Linnaeus 1758) | species |
| Fishes | Lycengraulis batesii (Günther 1868) | species |
| Fishes | Lycengraulis grossidens (Spix & Agassiz 1829) | species |
| Fishes | Macrodon ancylodon (Bloch & Schneider 1801) | species |
| Fishes | Megalops atlanticus Valenciennes 1847 | species |
| Fishes | Menticirrhus americanus (Linnaeus 1758) | species |
| Fishes | Menticirrhus littoralis (Holbrook, 1847) | species |
| Fishes | Metynnis maculatus (Kner 1858) | species |
| Fishes | *Microgobius meeki* Evermann & Marsh 1899 | species |
| Fishes | Micropogonias furnieri (Desmarest 1823) | species |
| Fishes | Moenkhausia oligolepis (Günther 1864) | species |
| Fishes | *Mugil brevirostris* Miranda Ribeiro 1915 | species |
| Fishes | Mugil curema Valenciennes 1836 | species |
| Fishes | Mugil incilis Hancock 1830 | species |
| Fishes | *Mugil liza* Valenciennes 1836 | species |
| Fishes | *Mycteroperca bonaci* (Poey 1860) | species |
| Fishes | *Mycteroperca tigris* (Valenciennes 1833) | species |
| Fishes | *Myripristis jacobus* Cuvier 1829 | species |
| Fishes | Nannostomus beckfordi Günther 1872 | species |
| Fishes | Nebris microps Cuvier 1830 | species |
| Fishes | Notarius grandicassis (Valenciennes 1840) | species |
| Fishes | *Ocyurus chrysurus* (Bloch 1791) | species |
| Fishes | Odontognathus mucronatus Lacepède,1800 | species |
| Fishes | *Odontoscion dentex* (Cuvier 1830) | species |
| Fishes | Ogcocephalus vespertilio (Linnaeus 1758) | species |
| Fishes | Oligoplites palometa (Cuvier 1832) | species |
| Fishes | Oligoplites saurus (Bloch & Schneider 1801) | species |
| Fishes | Ophichthus cylindroideus (Ranzani 1839) | species |
| Fishes | Opisthonema oglinum (Lesueur 1818) | species |
| Fishes | Oreochromis niloticus (Linnaeus 1758) | species |
| Fishes | *Pachypops fourcroi* (Lacepède 1802) | species |
| Fishes | *Paralichthys brasiliensis* (Ranzani 1842) | species |
| Fishes | Peckoltia greedoi Armbruster, Werneke & Tan 2015 | species |
| Fishes | Pellona castelnaeana Valenciennes 1847 | species |
| Fishes | Pellona flavipinnis (Valenciennes 1837) | species |
| Fishes | Peprilus paru (Linnaeus 1758) | species |
| Fishes | Pimelodella cristata (Müller & Troschel 1849) | species |
| Fishes | Pimelodus blochii Valenciennes 1840 | species |
| Fishes | Pimelodus ornatus Kner 1858 | species |
| Fishes | Plagioscion squamosissimus (Heckel 1840) | species |
| Fishes | Platydoras brachylecis Piorski, Garavello, Arce H. & Sabaj Pérez 2008 | species |
| Fishes | Poecilia reticulata Peters 1859 | species |
| Fishes | Poecilia sarrafae Bragança & Costa 2011 | species |
| Fishes | Polydactylus oligodon (Günther 1860) | species |
| Fishes | Polydactylus virginicus (Linnaeus 1758) | species |
| Fishes | *Pomacanthus paru* (Bloch 1787) | species |
| Fishes | Poptella compressa (Günther 1864) | species |
| Fishes | Potamorhina squamoralevis (Braga & Azpelicueta 1983) | species |
| Fishes | *Prionotus punctatus* (Bloch 1793) | species |
| Fishes | Prochilodus brevis Steindachner 1875 | species |
| Fishes | Prochilodus lacustris Steindachner 1907 | species |
| Fishes | Prochilodus nigricans Spix & Agassiz 1829 | species |
| Fishes | Psectrogaster rhomboides Eigenmann & Eigenmann 1889 | species |
| Fishes | Psellogrammus kennedyi (Eigenmann 1903) | species |
| Fishes | Pseudauchenipterus nodosus (Bloch 1794) | species |
| Fishes | Pseudobatos horkelii (Müller & Henle 1841) | species |
| Fishes | Pseudoplatystoma fasciatum (Linnaeus 1766) | species |
| Fishes | Pterengraulis atherinoides (Linnaeus 1766) | species |
| Fishes | Pterygoplichthys lituratus (Kner 1854) | species |
| Fishes | Pterygoplichthys parnaibae (Weber 1991) | species |
| Fishes | Pterygoplichthys punctatus (Kner 1854) | species |
| Fishes | Pygocentrus nattereri Kner 1858 | species |
| Fishes | Rhamdia quelen (Quoy & Gaimard 1824) | species |
| Fishes | Rhamphichthys atlanticus Triques 1999 | species |
| Fishes | Rhamphichthys rostratus (Linnaeus 1766) | species |
| Fishes | Rhamphichthys sp. | species |
| Fishes | *Rhinosardinia amazonica* (Steindachner 1879) | species |
| Fishes | Rhizoprionodon lalandii (Valenciennes 1839) | species |
| Fishes | *Rhomboplites aurorubens* (Cuvier 1829) | species |
| Fishes | Roeboides affinis (Günther 1868) | species |
| Fishes | Roeboides margareteae Lucena 2003 | species |
| Fishes | *Rypticus randalli* Courtenay 1967 | species |
| Fishes | Sardinella aurita Valenciennes 1847 | species |
| Fishes | Schizodon dissimilis (Garman 1890) | species |
| Fishes | Sciades couma (Valenciennes 1840) | species |
| Fishes | Sciades herzbergii (Bloch 1794) | species |
| Fishes | Sciades passany (Valenciennes, 1840) | species |
| Fishes | Sciades proops (Valenciennes 1840) | species |
| Fishes | Scomberomorus cavalla (Cuvier 1829) | species |
| Fishes | *Scorpaena plumieri* Bloch 1789 | species |
| Fishes | *Selene brownii* (Cuvier 1816) | species |
| Fishes | Selene setapinnis (Mitchill 1815) | species |
| Fishes | Selene vomer (Linnaeus 1758) | species |
| Fishes | Serrapinnus piaba (Lütken 1875) | species |
| Fishes | Serrasalmus brandtii Lütken 1875 | species |
| Fishes | Serrasalmus rhombeus (Linnaeus 1766) | species |
| Fishes | Sorubim lima (Bloch & Schneider 1801) | species |
| Fishes | Sphoeroides greeleyi Gilbert 1900 | species |
| Fishes | *Sphoeroides testudineus* (Linnaeus 1758) | species |
| Fishes | *Sphyraena barracuda* (Edwards 1771) | species |
| Fishes | *Stegastes variabilis* (Castelnau 1855) | species |
| Fishes | Steindachnerina notonota (Miranda Ribeiro 1937) | species |
| Fishes | Stellifer brasiliensis (Schultz 1945) | species |
| Fishes | Stellifer microps (Steindachner 1864) | species |
| Fishes | Stellifer naso (Jordan 1889) | species |
| Fishes | *Stellifer punctatissimus* (Meek & Hildebrand 1925) | species |
| Fishes | Stellifer rastrifer (Jordan 1889) | species |
| Fishes | Stellifer stellifer (Bloch 1790) | species |
| Fishes | Stellifer venezuelae (Schultz, 1945) | species |
| Fishes | Sternopygus macrurus (Bloch & Schneider 1801) | species |
| Fishes | Strongylura marina (Walbaum 1792) | species |
| Fishes | Strongylura timucu (Walbaum 1792) | species |
| Fishes | *Syacium micrurum* Ranzani 1842 | species |
| Fishes | Symphurus diomedeanus (Goode & Bean 1885) | species |
| Fishes | Symphurus plagiusa (Linnaeus 1766) | species |
| Fishes | Symphurus plagusia (Bloch & Schneider 1801) | species |
| Fishes | Synbranchus marmoratus Bloch 1795 | species |
| Fishes | *Synodus foetens* (Linnaeus 1766) | species |
| Fishes | *Thalassophryne maculosa* Günther 1861 | species |
| Fishes | Thalassophryne nattereri Steindachner 1876 | species |
| Fishes | Trachelyopterus galeatus (Linnaeus 1766) | species |
| Fishes | Trachinotus cayennensis Cuvier, 1832 | species |
| Fishes | Trachinotus falcatus (Linnaeus 1758) | species |
| Fishes | Trichiurus lepturus Linnaeus 1758 | species |
| Fishes | Trinectes maculatus (Bloch & Schneider, 1801) | species |
| Fishes | *Trinectes paulistanus* (Miranda Ribeiro 1915) | species |
| Fishes | Triportheus signatus (Garman 1890) | species |
| Fishes | Tylosurus acus (Lacepède 1803) | species |
| Mammalia | Akodon Meyen, 1833 | genus |
| Mammalia | Artibeus (Dermanura) P. Gervais, 1856 | genus |
| Mammalia | Bradypus (Bradypus) Linnaeus, 1758 | genus |
| Mammalia | Cabassous McMurtrie, 1831 | genus |
| Mammalia | Chiroderma W. Peters, 1860 | genus |
| Mammalia | Cryptonanus Voss, Lunde & Jansa, 2005 | genus |
| Mammalia | Dasyprocta Illiger, 1811 | genus |
| Mammalia | Dasypus Linnaeus, 1758 | genus |
| Mammalia | Didelphis Linnaeus, 1758 | genus |
| Mammalia | Euryoryzomys Weksler et al. 2006 | genus |
| Mammalia | Galea Meyen, 1833 | genus |
| Mammalia | Glossophaga Geoffroy, 1818 | genus |
| Mammalia | Gracilinanus Gardner & Creighton, 1989 | genus |
| Mammalia | Guerlinguetus Gray, 1821 | genus |
| Mammalia | Hylaeamys Weksler et al., 2006 | genus |
| Mammalia | Leopardus Gray, 1842 | genus |
| Mammalia | Marmosops Matschie, 1916 | genus |
| Mammalia | Mazama Rafinesque, 1917 | genus |
| Mammalia | Monodelphis Burnett, 1830 | genus |
| Mammalia | Oecomys Thomas, 1906 | genus |
| Mammalia | Oligoryzomys Bangs, 1900 | genus |
| Mammalia | Aotus Illiger, 1811 | species |
| Mammalia | Aotus infulatus (Kuhl, 1820) | species |
| Mammalia | Artibeus (Artibeus) lituratus (Olfers, 1818) | species |
| Mammalia | Artibeus (Artibeus) obscurus (Schinz, 1821) | species |
| Mammalia | Artibeus (Artibeus) planirostris (Spix, 1823) | species |
| Mammalia | Artibeus (Dermanura) cinereus (Gervais, 1856) | species |
| Mammalia | Bradypus (Bradypus) variegatus Schinz, 1825 | species |
| Mammalia | Cabassous unicinctus (Linnaeus, 1758) | species |
| Mammalia | Caluromys philander (Linnaeus, 1758) | species |
| Mammalia | Carollia brevicauda (Schinz, 1821) | species |
| Mammalia | Carollia perspicillata (Linnaeus, 1758) | species |
| Mammalia | Cerdocyon thous (Linnaeus, 1766) | species |
| Mammalia | Choloepus didactylus (Linnaeus, 1758) | species |
| Mammalia | Coendou (Coendou) prehensilis (Linnaeus, 1758) | species |
| Mammalia | Conepatus semistriatus (Boddaert, 1785) | species |
| Mammalia | Cuniculus paca (Linnaeus, 1766) | species |
| Mammalia | Cyclopes didactylus (Linnaeus, 1758) | species |
| Mammalia | Dasyprocta azarae Lichtenstein, 1823 | species |
| Mammalia | Dasyprocta leporina (Linnaeus, 1758) | species |
| Mammalia | Dasyprocta prymnolopha Wagler, 1831 | species |
| Mammalia | Dasypus (Dasypus) novemcinctus Linnaeus, 1758 | species |
| Mammalia | Dasypus (Muletia) septemcinctus Linnaeus, 1758 | species |
| Mammalia | Desmodus rotundus (É. Geoffroy St.-Hilaire, 1810) | species |
| Mammalia | Diaemus youngii (Jentink, 1893) | species |
| Mammalia | Diclidurus albus Wied-Neuwied, 1820 | species |
| Mammalia | Didelphis albiventris Lund, 1840 | species |
| Mammalia | Didelphis marsupialis Linnaeus, 1758 | species |
| Mammalia | Eira barbara (Linnaeus, 1758) | species |
| Mammalia | Eptesicus brasiliensis (Desmarest, 1819) | species |
| Mammalia | Eumops perotis (Schinz, 1821) | species |
| Mammalia | Euphractus sexcinctus (Linnaeus, 1758) | species |
| Mammalia | Galea spixii (Wagler, 1831) | species |
| Mammalia | Galictis vittata (Schreber, 1776) | species |
| Mammalia | Glossophaga soricina (Pallas, 1766) | species |
| Mammalia | Glyphonycteris sylvestris Thomas, 1896 | species |
| Mammalia | Gracilinanus agilis (Burmeister, 1854) | species |
| Mammalia | Gracilinanus emiliae (Thomas, 1909) | species |
| Mammalia | Guerlinguetus aestuans (Linnaeus, 1766) | species |
| Mammalia | Guerlinguetus brasiliensis (Gmelin, 1788) | species |
| Mammalia | Herpailurus yagouaroundi (É. Geoffroy Saint-Hilaire, 1803) | species |
| Mammalia | Holochilus sciureus Wagner, 1842 | species |
| Mammalia | Hydrochoerus hydrochaeris (Linnaeus, 1766) | species |
| Mammalia | Leopardus emiliae (Thomas, 1914) | species |
| Mammalia | Leopardus pardalis (Linnaeus, 1758) | species |
| Mammalia | Leopardus wiedii (Schinz, 1821) | species |
| Mammalia | Lonchophylla mordax Thomas, 1903 | species |
| Mammalia | Lontra longicaudis (Olfers, 1818) | species |
| Mammalia | Lophostoma brasiliense Peters, 1867 | species |
| Mammalia | Lophostoma carrikeri (Allen, 1910) | species |
| Mammalia | Lycalopex vetulus (Lund, 1842) | species |
| Mammalia | Makalata didelphoides (Desmarest, 1817) | species |
| Mammalia | Makalata obscura (Wagner, 1840) | species |
| Mammalia | Marmosa (Marmosa) murina (Linnaeus, 1758) | species |
| Mammalia | Marmosa (Micoureus) demerarae (Thomas, 1905) | species |
| Mammalia | Marmosops (Sciophanes) woodalli (Pine, 1981) | species |
| Mammalia | Mazama gouazoubira (Fischer, 1814) | species |
| Mammalia | Megaptera novaeangliae (Borowski, 1781) | species |
| Mammalia | Metachirus myosuros (Temminck, 1824) | species |
| Mammalia | Micronycteris megalotis (Gray, 1842) | species |
| Mammalia | Micronycteris minuta (Gervais, 1856) | species |
| Mammalia | Molossus coibensis Allen, 1904 | species |
| Mammalia | Molossus molossus (Pallas, 1766) | species |
| Mammalia | Monodelphis (Monodelphis) domestica (Wagner, 1842) | species |
| Mammalia | Mus musculus Linnaeus, 1758 | species |
| Mammalia | Myotis nigricans (Schinz, 1821) | species |
| Mammalia | Myotis riparius Handley, 1960 | species |
| Mammalia | Nasua nasua (Linnaeus, 1766) | species |
| Mammalia | Necromys lasiurus (Lund, 1841) | species |
| Mammalia | Noctilio leporinus (Linnaeus, 1758) | species |
| Mammalia | Oligoryzomys mattogrossae (Allen, 1916) | species |
| Mammalia | Oligoryzomys nigripes (Olfers, 1818) | species |
| Mammalia | Oxymycterus amazonicus Hershkovitz, 1994 | species |
| Mammalia | Philander opossum (Linnaeus, 1758) | species |
| Mammalia | Phyllostomus discolor (Wagner, 1843) | species |
| Mammalia | Phyllostomus hastatus (Pallas, 1767) | species |
| Mammalia | Physeter macrocephalus Linnaeus, 1758 | species |
| Mammalia | Platyrrhinus recifinus (Thomas, 1901) | species |
| Mammalia | Procyon cancrivorus Cuvier, 1798 | species |
| Mammalia | Proechimys guyannensis (É. Geoffroy St.-Hilaire, 1803) | species |
| Mammalia | Proechimys roberti Thomas, 1901 | species |
| Mammalia | Pteronotus personatus (Wagner, 1843) | species |
| Mammalia | Pteronotus rubiginosus (Wagner, 1843) | species |
| Mammalia | Pteronura brasiliensis (Zimmermann, 1780) | species |
| Mammalia | Rattus rattus (Linnaeus, 1758) | species |
| Mammalia | Rhinophylla pumilio Peters, 1865 | species |
| Mammalia | Rhipidomys emiliae (Allen, 1916) | species |
| Mammalia | Rhynchonycteris naso (Wied-Neuwied, 1820) | species |
| Mammalia | Saccopteryx bilineata (Temminck, 1838) | species |
| Mammalia | Saccopteryx canescens Thomas, 1901 | species |
| Mammalia | Saguinus (Saguinus) ursula Hoffmannsegg, 1807 | species |
| Mammalia | Saimiri collinsi Osgood, 1916 | species |
| Mammalia | Sapajus apella (Linnaeus, 1758) | species |
| Mammalia | Sotalia fluviatilis (Gervais & Deville, 1853) | species |
| Mammalia | Sotalia guianensis (Van Bénéden, 1864) | species |
| Mammalia | Sturnira lilium (É. Geoffroy St.-Hilaire, 1810) | species |
| Mammalia | Sylvilagus brasiliensis (Linnaeus, 1758) | species |
| Mammalia | Tamandua tetradactyla (Linnaeus, 1758) | species |
| Mammalia | Thyroptera tricolor Spix, 1823 | species |
| Mammalia | Trichechus manatus Linnaeus, 1758 | species |
| Mammalia | Uroderma bilobatum Peters, 1866 | species |
| Mammalia | Vampyriscus bidens (Dobson, 1878) | species |
| Phytoplankton | Bacillariophyceae Haeckel, 1878 | class |
| Phytoplankton | Actinocyclus Ehrenberg, 1837 | genus |
| Phytoplankton | Actinoptychus Ehrenberg, 1843 | genus |
| Phytoplankton | Alexandrium Halim, 1960 | genus |
| Phytoplankton | Amphora Ehrenberg ex Kützing, 1844 | genus |
| Phytoplankton | Anabaena Bory ex Bornet & Flahault, 1886 | genus |
| Phytoplankton | Ankistrodesmus Corda, 1838 | genus |
| Phytoplankton | Aphanocapsa C.Nägeli, 1849 | genus |
| Phytoplankton | Asteromphalus Ehrenberg, 1844 | genus |
| Phytoplankton | Aulacodiscus Ehrenberg, 1844 | genus |
| Phytoplankton | Auliscus Ehrenberg, 1843 | genus |
| Phytoplankton | Biddulphia S.F.Gray, 1821 | genus |
| Phytoplankton | Borzia F.Cohn ex M.Gomont, 1892 | genus |
| Phytoplankton | Botryococcus Kützing, 1849 | genus |
| Phytoplankton | Brigantedinium P.C.Reid, 1977 | genus |
| Phytoplankton | Caloneis Cleve, 1894 | genus |
| Phytoplankton | Campylodiscus Ehrenberg ex Kützing, 1844 | genus |
| Phytoplankton | Ceratium F.Schrank, 1793 | genus |
| Phytoplankton | Chaetoceros Ehrenberg, 1844 | genus |
| Phytoplankton | Chlamydomonas Ehrenberg, 1833 | genus |
| Phytoplankton | Chlorococcum Meneghini, 1842 | genus |
| Phytoplankton | Cladopyxis F.Stein, 1883 | genus |
| Phytoplankton | Cocconeis Ehrenberg, 1836 | genus |
| Phytoplankton | Coelastrum Nägeli, 1849 | genus |
| Phytoplankton | Corethron Castracane, 1886 | genus |
| Phytoplankton | Coscinodiscus Ehrenberg, 1839 | genus |
| Phytoplankton | Coscinodiscus Ehrenberg, 1839 | genus |
| Phytoplankton | Cyclotella (Kützing) Brébisson, 1838 | genus |
| Phytoplankton | Cymatosira Grunow, 1862 | genus |
| Phytoplankton | Cymbella C.Agardh, 1830 | genus |
| Phytoplankton | Delphineis G.W.Andrews, 1977 | genus |
| Phytoplankton | Dimorphococcus Braun, 1855 | genus |
| Phytoplankton | Dinophysis Ehrenberg, 1839 | genus |
| Phytoplankton | Diploneis Cleve, P.T. (1894) | genus |
| Phytoplankton | Entomoneis Ehrenberg, 1845 | genus |
| Phytoplankton | Eudorina Ehrenberg, 1832 | genus |
| Phytoplankton | Euglena Ehrenberg, 1830 | genus |
| Phytoplankton | Eunotia Ehrenberg, 1837 | genus |
| Phytoplankton | Eupodiscus J.W.Bailey, 1851 | genus |
| Phytoplankton | Fragilaria Lyngbye, 1819 | genus |
| Phytoplankton | Fragilariopsis Hustedt, 1913 | genus |
| Phytoplankton | Frustulia Rabenhorst, 1853 | genus |
| Phytoplankton | Glenodinium Ehrenberg, 1836 | genus |
| Phytoplankton | Gomphonema Ehrenberg, 1832 | genus |
| Phytoplankton | Gonyaulax Diesing, 1866 | genus |
| Phytoplankton | Grammatophora Ehrenberg, 1840 | genus |
| Phytoplankton | Guinardia H.Peragallo, 1892 | genus |
| Phytoplankton | Gymnodinium F.Stein, 1878 | genus |
| Phytoplankton | Gyrosigma Hassall, 1845 | genus |
| Phytoplankton | Hemiaulus P.A.C. Heiberg, 1863 | genus |
| Phytoplankton | Licmophora C.Agardh, 1827 | genus |
| Phytoplankton | Lithodesmium Ehrenberg, 1839 | genus |
| Phytoplankton | Lyngbya C.Agardh ex Gomont, 1892 | genus |
| Phytoplankton | Lyrella Karayeva, 1978 | genus |
| Phytoplankton | Mastogloia Thwaites ex W.Smith, 1856 | genus |
| Phytoplankton | Melosira C.Agardh, 1824 | genus |
| Phytoplankton | Merismopedia Meyen, 1839 | genus |
| Phytoplankton | Microcystis Lemmermann, 1907 | genus |
| Phytoplankton | Mougeotia C.Agardh, 1824 | genus |
| Phytoplankton | Navicula Bory, 1822 | genus |
| Phytoplankton | Nitzschia Hassall, 1845 | genus |
| Phytoplankton | Nostoc Vaucher ex Bornet & Flahault, 1886 | genus |
| Phytoplankton | Odontella C.Agardh, 1832 | genus |
| Phytoplankton | Oedogonium Link ex Hirn, 1900 | genus |
| Phytoplankton | Oocystis Nägeli ex A.Braun, 1855 | genus |
| Phytoplankton | Oscillatoria Vaucher ex Gomont, 1892 | genus |
| Phytoplankton | Oxytoxum Stein, 1883 | genus |
| Phytoplankton | Paralia Heiberg, 1863 | genus |
| Phytoplankton | Pediastrum Meyen, 1829 | genus |
| Phytoplankton | Peranema Dujardin, 1841 | genus |
| Phytoplankton | Peridinium Ehrenberg, 1830 | genus |
| Phytoplankton | Phacus Dujardin, 1841 | genus |
| Phytoplankton | Pinnularia Ehrenberg, 1843 | genus |
| Phytoplankton | Plagiogramma Greville, 1859 | genus |
| Phytoplankton | Plagiotropis Pfitzer, 1871 | genus |
| Phytoplankton | Planktothrix Anagnostidis & Komárek, 1988 | genus |
| Phytoplankton | Pleurococcus Meneghini, 1837 | genus |
| Phytoplankton | Pleurosigma W.Smith, 1852 | genus |
| Phytoplankton | Pleurosigma W. Smith, 1852 | genus |
| Phytoplankton | Pleurosira (Meneghini) Trevisan, 1848 | genus |
| Phytoplankton | Prorocentrum Ehrenberg, 1834 | genus |
| Phytoplankton | Protoperidinium Bergh, 1881 | genus |
| Phytoplankton | Pseudanabaena Lauterborn, 1915 | genus |
| Phytoplankton | Pseudo-nitzschia H.Peragallo, 1900 | genus |
| Phytoplankton | Pyrophacus F.Stein, 1883 | genus |
| Phytoplankton | Rhabdonema Kützing, 1844 | genus |
| Phytoplankton | Rhizosolenia Brightwell, 1858 | genus |
| Phytoplankton | Rhopalodia O.Müller, 1895 | genus |
| Phytoplankton | Scenedesmus Meyen, 1829 | genus |
| Phytoplankton | Skeletonema Greville, 1865 | genus |
| Phytoplankton | Sphaerocystis R.Chodat, 1897 | genus |
| Phytoplankton | Spirogyra Link, 1820 | genus |
| Phytoplankton | Spirulina Turpin ex Gomont, 1892 | genus |
| Phytoplankton | Staurastrum Meyen ex Ralfs, 1848 | genus |
| Phytoplankton | Stauroneis Ehrenberg, 1843 | genus |
| Phytoplankton | Surirella Turpin, 1828 | genus |
| Phytoplankton | Synechococcus Nägeli, 1849 | genus |
| Phytoplankton | Synechocystis C.Sauvageau, 1892 | genus |
| Phytoplankton | Synedra Ehrenberg, 1830 | genus |
| Phytoplankton | Tetraedron Kützing, 1845 | genus |
| Phytoplankton | Thalassionema Grunow ex Mereschkowsky, 1902 | genus |
| Phytoplankton | Thalassiosira Cleve, 1873 | genus |
| Phytoplankton | Trachelomonas Ehrenberg, 1834 | genus |
| Phytoplankton | Triceratium Ehrenberg, 1839 | genus |
| Phytoplankton | Trichodesmium Ehrenberg ex Gomont, 1892 | genus |
| Phytoplankton | Achnanthes adnata Bory 1822 | species |
| Phytoplankton | Achnanthes brevipes var. intermedia (Kützing) Cleve 1895 | species |
| Phytoplankton | Actinocyclus curvatulus Janisch 1878 | species |
| Phytoplankton | Actinocyclus ingens var. ovalis R.Gersonde 1990 | species |
| Phytoplankton | Actinocyclus nebulosus M.Peragallo 1902 | species |
| Phytoplankton | Actinocyclus octonarius Ehrenberg 1837 | species |
| Phytoplankton | Actinocyclus octonarius var. crassus (W.Smith) Hendey 1954 | species |
| Phytoplankton | Actinocyclus octonarius var. ralfsii (W.Smith) Hendey 1954 | species |
| Phytoplankton | Actinocyclus ralfsii (W.Smith) Ralfs 1861 | species |
| Phytoplankton | Actinoptychus adriaticus Grunow 1863 | species |
| Phytoplankton | Actinoptychus annulatus (Wallich) Grunow 1883 | species |
| Phytoplankton | Actinoptychus aster J.-J.Brun 1892 | species |
| Phytoplankton | Actinoptychus campanulifer Schmidt 1875 | species |
| Phytoplankton | Actinoptychus minutus Greville 1866 | species |
| Phytoplankton | Actinoptychus octonarius (Ehrenberg) Kützing 1844 | species |
| Phytoplankton | Actinoptychus parvus A.Mann 1925 | species |
| Phytoplankton | Actinoptychus senarius (Ehrenberg) Ehrenberg 1843 | species |
| Phytoplankton | Actinoptychus splendens (Shadbolt) Ralfs 1861 | species |
| Phytoplankton | Actinoptychus vulgaris Schumann 1867 | species |
| Phytoplankton | Alveus marinus (Grunow) Kaczmarska & Fryxell 1996 | species |
| Phytoplankton | Amphipentas pentacrinus Ehrenberg 1841 | species |
| Phytoplankton | Amphiprora alata (Ehrenberg) Kützing 1844 | species |
| Phytoplankton | Amphora laevis W.Gregory 1857 | species |
| Phytoplankton | Amphora ovalis (Kützing) Kützing 1844 | species |
| Phytoplankton | Amphora proteus W.Gregory 1857 | species |
| Phytoplankton | Anagnostidinema amphibium (C.Agardh ex Gomont) Strunecký, Bohunická, J.R.Johansen & J.Komárek 2017 | species |
| Phytoplankton | Ankistrodesmus densus Korshikov 1953 | species |
| Phytoplankton | Ankistrodesmus falcatus (Corda) Ralfs 1848 | species |
| Phytoplankton | Asterionellopsis glacialis (Castracane) Round 1990 | species |
| Phytoplankton | Aulacodiscus kittonii Arnott ex Ralfs 1861 | species |
| Phytoplankton | Aulacodiscus margaritaceus Ralfs 1861 | species |
| Phytoplankton | Bacillaria paxillifera (O.F.Müller) T.Marsson 1901 | species |
| Phytoplankton | Bacteriastrum delicatulum Cleve 1897 | species |
| Phytoplankton | Bacteriastrum furcatum Shadbolt 1854 | species |
| Phytoplankton | Bacteriastrum hyalinum Lauder 1864 | species |
| Phytoplankton | Bellerochea horologicalis Stosch 1977 | species |
| Phytoplankton | Bellerochea malleus (Brightwell) Van Heurck 1885 | species |
| Phytoplankton | Biddulphia alternans (Bailey) Van Heurck 1885 | species |
| Phytoplankton | Biddulphia biddulphiana (J.E.Smith) Boyer 1900 | species |
| Phytoplankton | Biddulphia tridens (Ehrenberg) Ehrenberg 1841 | species |
| Phytoplankton | Biddulphiella tridens (Ehrenberg) P.A.Sims & M.P.Ashworth 2022 | species |
| Phytoplankton | Binuclearia lauterbornii (Schmidle) Proshkina-Lavrenko 1966 | species |
| Phytoplankton | Caloneis permagna (Bailey) Cleve 1894 | species |
| Phytoplankton | Campylodiscus clypeus (Ehrenberg) Ehrenberg ex Kützing 1844 | species |
| Phytoplankton | Campylodiscus neofastuosus Ruck & Nakov 2016 | species |
| Phytoplankton | Campylosira cymbelliformis (A.W.F.Schmidt) Grunow ex Van Heurck 1885 | species |
| Phytoplankton | Cerataulina H.Peragallo ex F.Schütt, 1896 | species |
| Phytoplankton | Cerataulina pelagica (Cleve) Hendey 1937 | species |
| Phytoplankton | Ceratium hirundinella (O.F.Müller) Dujardin 1841 | species |
| Phytoplankton | Chaetoceros abnormis Proshkina-Lavrenko 1953 | species |
| Phytoplankton | Chaetoceros aequatorialis Cleve 1901 | species |
| Phytoplankton | Chaetoceros affinis Lauder 1864 | species |
| Phytoplankton | Chaetoceros atlanticus Cleve 1873 | species |
| Phytoplankton | Chaetoceros atlanticus var. neapolitanus (Schroeder) Hustedt 1930 | species |
| Phytoplankton | Chaetoceros brevis F.Schütt 1895 | species |
| Phytoplankton | Chaetoceros coarctatus Lauder 1864 | species |
| Phytoplankton | Chaetoceros compressus Lauder 1864 | species |
| Phytoplankton | Chaetoceros constrictus Gran 1897 | species |
| Phytoplankton | Chaetoceros curvisetus Cleve 1889 | species |
| Phytoplankton | Chaetoceros danicus Cleve 1889 | species |
| Phytoplankton | Chaetoceros debilis Cleve 1894 | species |
| Phytoplankton | Chaetoceros decipiens Cleve 1873 | species |
| Phytoplankton | Chaetoceros densus (Cleve) Cleve 1899 | species |
| Phytoplankton | Chaetoceros diadema (Ehrenberg) Gran 1897 | species |
| Phytoplankton | Chaetoceros didymus Ehrenberg 1845 | species |
| Phytoplankton | Chaetoceros diversus Cleve 1873 | species |
| Phytoplankton | Chaetoceros gracilis Pantocsek 1892 | species |
| Phytoplankton | Chaetoceros holsaticus F.Schütt 1895 | species |
| Phytoplankton | Chaetoceros laciniosus F.Schütt 1895 | species |
| Phytoplankton | Chaetoceros lorenzianus Grunow 1863 | species |
| Phytoplankton | Chaetoceros pendulus Karsten 1905 | species |
| Phytoplankton | Chaetoceros peruvianus Brightwell 1856 | species |
| Phytoplankton | Chaetoceros pseudocurvisetus Mangin 1910 | species |
| Phytoplankton | Chaetoceros rostratus Ralfs 1864 | species |
| Phytoplankton | Chaetoceros simplex Ostenfeld 1902 | species |
| Phytoplankton | Chaetoceros subtilis Cleve 1896 | species |
| Phytoplankton | Chaetoceros subtilis var. abnormis Prosckina-Lavrenko 1961 | species |
| Phytoplankton | Chaetoceros teres Cleve 1896 | species |
| Phytoplankton | Chroococcus minutus (Kützing) Nägeli 1849 | species |
| Phytoplankton | Chroococcus turgidus (Kützing) Nägeli 1849 | species |
| Phytoplankton | Chrysanthemodiscus floriatus A.Mann 1925 | species |
| Phytoplankton | Closterium kuetzingii Brébisson 1856 | species |
| Phytoplankton | Closterium navicula (Brébisson) Lütkemüller 1905 | species |
| Phytoplankton | Closterium parvulum Nägeli 1849 | species |
| Phytoplankton | Closterium setaceum Ehrenberg ex Ralfs 1848 | species |
| Phytoplankton | Coelastrum pseudomicroporum Korshikov 1953 | species |
| Phytoplankton | Corethron hystrix Hensen 1887 | species |
| Phytoplankton | Corethron pennatum (Grunow) Ostenfeld 1909 | species |
| Phytoplankton | Coronia impressa (Grunow) Ruck & Guiry 2016 | species |
| Phytoplankton | Coscinodiscopsis jonesiana (Greville) E.A.Sar & I.Sunesen 2008 | species |
| Phytoplankton | Coscinodiscus apiculatus Ehrenberg 1844 | species |
| Phytoplankton | Coscinodiscus asteromphalus Ehrenberg 1844 | species |
| Phytoplankton | Coscinodiscus centralis Ehrenberg 1839 | species |
| Phytoplankton | Coscinodiscus concinnus W.Smith 1856 | species |
| Phytoplankton | Coscinodiscus curvatulus Grunow 1878 | species |
| Phytoplankton | Coscinodiscus gigas Ehrenberg 1841 | species |
| Phytoplankton | Coscinodiscus granii L.F.Gough 1905 | species |
| Phytoplankton | Coscinodiscus marginatus Ehrenberg 1843 | species |
| Phytoplankton | Coscinodiscus nitidulus Grunow 1878 | species |
| Phytoplankton | Coscinodiscus obscurus A.W.F.Schmidt 1878 | species |
| Phytoplankton | Coscinodiscus oculus-iridis (Ehrenberg) Ehrenberg 1840 | species |
| Phytoplankton | Coscinodiscus perforatus Ehrenberg 1844 | species |
| Phytoplankton | Coscinodiscus radiatus Ehrenberg 1840 | species |
| Phytoplankton | Coscinodiscus rothii (Ehrenberg) Grunow 1878 | species |
| Phytoplankton | Coscinodiscus wailesii Gran & Angst 1931 | species |
| Phytoplankton | Cosmioneis pusilla (W.Smith) D.G.Mann & A.J.Stickle 1990 | species |
| Phytoplankton | Craticula cuspidata (Kutzing) D.G.Mann 1990 | species |
| Phytoplankton | Cyclotella litoralis Lange & Syvertsen 1989 | species |
| Phytoplankton | Cyclotella meneghiniana Kützing 1844 | species |
| Phytoplankton | Cyclotella striata (Kützing) Grunow 1880 | species |
| Phytoplankton | Cyclotella stylorum Brightwell 1860 | species |
| Phytoplankton | Cylindrotheca closterium (Ehrenberg) Reimann & J.C.Lewin 1964 | species |
| Phytoplankton | Cymatosira belgica Grunow 1881 | species |
| Phytoplankton | Cymatosira lorenziana Grunow 1862 | species |
| Phytoplankton | Delphineis surirella (Ehrenberg) G.W.Andrews 1981 | species |
| Phytoplankton | Desmodesmus opoliensis (P.G.Richter) E.Hegewald 2000 | species |
| Phytoplankton | Dictyocha fibula Ehrenberg 1839 | species |
| Phytoplankton | Dictyosphaerium Nägeli, 1849 | species |
| Phytoplankton | Dimorphococcus lunatus A.Braun 1855 | species |
| Phytoplankton | Diploneis bombus (Ehrenberg) Ehrenberg 1853 | species |
| Phytoplankton | Diploneis caffra (M.H.Giffen) Witkowski, Lange-Bertalot & Metzeltin 2000 | species |
| Phytoplankton | Diploneis crabro (Ehrenberg) Ehrenberg 1854 | species |
| Phytoplankton | Diploneis gruendleri (A.W.F.Schmidt) Cleve 1894 | species |
| Phytoplankton | Diploneis interrupta (Kützing) Cleve 1894 | species |
| Phytoplankton | Diploneis ovalis (Hilse) Cleve 1891 | species |
| Phytoplankton | Diploneis smithii (Brébisson) Cleve 1894 | species |
| Phytoplankton | Diploneis vacillans (A.W.F.Schmidt) Cleve 1894 | species |
| Phytoplankton | Diploneis weissflogii (A.W.F.Schmidt) Cleve 1894 | species |
| Phytoplankton | Discostella stelligera (Cleve & Grunow) Houk & Klee 2004 | species |
| Phytoplankton | Ditylum brightwellii (T.West) Grunow, 1885 | species |
| Phytoplankton | Ditylum sol (Grunow) De Toni 1894 | species |
| Phytoplankton | Entomoneis alata (Ehrenberg) Ehrenberg 1845 | species |
| Phytoplankton | Entomoneis ornata (Bailey) Reimer 1975 | species |
| Phytoplankton | Euglena acus (O.F.Müller) Ehrenberg 1830 | species |
| Phytoplankton | Euglena gracilis G.A.Klebs 1883 | species |
| Phytoplankton | Euglena oxyuris Schmarda 1846 | species |
| Phytoplankton | Euglenaformis proxima (P.A.Dangeard) M.S.Bennett & Triemer 2014 | species |
| Phytoplankton | Eunotia minor (Kützing) Grunow 1881 | species |
| Phytoplankton | Eunotia pectinalis (Kützing) Rabenhorst 1864 | species |
| Phytoplankton | Eunotogramma debile Grunow 1883 | species |
| Phytoplankton | Eupodiscus antiquus (Cox) Hanna 1932 | species |
| Phytoplankton | Eupodiscus radiatus Bailey 1851 | species |
| Phytoplankton | Fragilaria capucina Desmazières 1830 | species |
| Phytoplankton | Fragilaria rumpens (Kützing) G.W.F.Carlson 1913 | species |
| Phytoplankton | Frickea lewisiana (Greville) Heiden 1906 | species |
| Phytoplankton | Frustulia asymmetrica (Cleve) Hustedt 1954 | species |
| Phytoplankton | Frustulia interposita (Lewis) De Toni 1891 | species |
| Phytoplankton | Frustulia rhomboides (Ehrenberg) De Toni 1891 | species |
| Phytoplankton | Grammatophora hamulifera Kützing 1844 | species |
| Phytoplankton | Grammatophora marina (Lyngbye) Kützing 1844 | species |
| Phytoplankton | Grammatophora oceanica Ehrenberg 1840 | species |
| Phytoplankton | Grammatophora undulata Ehrenberg 1840 | species |
| Phytoplankton | Guinardia delicatula (Cleve) Hasle 1997 | species |
| Phytoplankton | Guinardia flaccida (Castracane) H.Peragallo 1892 | species |
| Phytoplankton | Guinardia striata (Stolterfoth) Hasle 1996 | species |
| Phytoplankton | Gyrosigma attenuatum (Kützing) Rabenhorst 1853 | species |
| Phytoplankton | Gyrosigma balticum (Ehrenberg) Rabenhorst 1853 | species |
| Phytoplankton | Gyrosigma fasciola (Ehrenberg) J.W.Griffith & Henfrey 1856 | species |
| Phytoplankton | Gyrosigma macrum (W.Smith) J.W.Griffith & Henfrey 1856 | species |
| Phytoplankton | Hantzschia amphioxys (Ehrenberg) Grunow 1880 | species |
| Phytoplankton | Helicotheca tamesis (Shrubsole) M.Ricard 1987 | species |
| Phytoplankton | Hemiaulus chinensis Greville, 1865 | species |
| Phytoplankton | Hemiaulus sinensis Greville, 1865 | species |
| Phytoplankton | Hemiaulus indicus Karsten 1907 | species |
| Phytoplankton | Hendeyella dubia (Grunow) Li, Witkowski & Ashworth, nom. inval. 2016 | species |
| Phytoplankton | Hobaniella longicruris (Greville) P.A.Sims & D.M.Williams 2018 | species |
| Phytoplankton | Hydrosera triquetra Wallich 1858 | species |
| Phytoplankton | Kamptonema formosum (Bory ex Gomont) Strunecký, Komárek & J.Smarda 2014 | species |
| Phytoplankton | Lauderia annulata Cleve 1873 | species |
| Phytoplankton | Lepocinclis acus (O.F.Müller) B.Marin & Melkonian 2003 | species |
| Phytoplankton | Lepocinclis acus var. rigida (E.F.W.Hüber) Jurán 2017 | species |
| Phytoplankton | Lepocinclis oxyuris (Schmarda) B.Marin & Melkonian 2003 | species |
| Phytoplankton | Lepocinclis tripteris (Dujardin) B.Marin & Melkonian 2003 | species |
| Phytoplankton | Leptocylindrus danicus Cleve 1889 | species |
| Phytoplankton | Leptocylindrus minimus Gran 1915 | species |
| Phytoplankton | Leptolyngbya foveolarum (Gomont) Anagnostidis & Komárek 1988 | species |
| Phytoplankton | Leptolyngbya fragilis (Gomont) Anagnostidis & Komárek 1988 | species |
| Phytoplankton | Leptolyngbya tenuis (Gomont) Anagnostidis & Komárek 1988 | species |
| Phytoplankton | Licmophora remulus (Grunow) Grunow 1867 | species |
| Phytoplankton | Limnococcus limneticus (Lemmermann) Komárková, Jezberová, O.Komárek & Zapomelová 2010 | species |
| Phytoplankton | Lithodesmium undulatum Ehrenberg 1839 | species |
| Phytoplankton | Lyngbya semiplena J.Agardh ex Gomont 1892 | species |
| Phytoplankton | Lyrella lyra (Ehrenberg) Karayeva 1978 | species |
| Phytoplankton | Melosira moniliformis (Link) C.Agardh 1824 | species |
| Phytoplankton | Melosira nummuloides C.Agardh 1824 | species |
| Phytoplankton | Melosira sulcata (Ehrenberg) Kützing 1844 | species |
| Phytoplankton | Merismopedia elegans A.Braun ex Kützing 1849 | species |
| Phytoplankton | Merismopedia glauca (Ehrenberg) Kützing 1845 | species |
| Phytoplankton | Merismopedia tenuissima Lemmermann 1898 | species |
| Phytoplankton | Microcystis aeruginosa (Kützing) Kützing 1846 | species |
| Phytoplankton | Microcystis botrys Teiling 1942 | species |
| Phytoplankton | Microcystis viridis (A.Braun) Lemmermann 1903 | species |
| Phytoplankton | Monactinus simplex (Meyen) Corda 1839 | species |
| Phytoplankton | Mucidosphaerium pulchellum (H.C.Wood) C.Bock, Proschold & Krienitz 2011 | species |
| Phytoplankton | Navicula normalis Hustedt 1955 | species |
| Phytoplankton | Navicula pennata A.W.F.Schmidt 1876 | species |
| Phytoplankton | Navicula transitans var. derasa (Grunow) Cleve 1883 | species |
| Phytoplankton | Navicula viridula (Kützing) Ehrenberg 1836 | species |
| Phytoplankton | Neocalyptrella robusta (G.Norman ex Ralfs) Hernández-Becerril & Castillo 1997 | species |
| Phytoplankton | Nitzschia obtusa f. scalpelliformis Grunow in Cleve & Molle, 1879 | species |
| Phytoplankton | Nitzschia amphibia Grunow 1862 | species |
| Phytoplankton | Nitzschia bilobata W.Smith 1853 | species |
| Phytoplankton | Nitzschia dubia W.Smith 1853 | species |
| Phytoplankton | Nitzschia fasciculata (Grunow) Grunow 1881 | species |
| Phytoplankton | Nitzschia frustulum (Kützing) Grunow 1880 | species |
| Phytoplankton | Nitzschia incurva var. lorenziana R.Ross 1986 | species |
| Phytoplankton | Nitzschia lanceolata W.Smith 1853 | species |
| Phytoplankton | Nitzschia longa Grunow 1880 | species |
| Phytoplankton | Nitzschia longissima (Brébisson) Ralfs 1861 | species |
| Phytoplankton | Nitzschia longissima var. closterium (Ehrenberg) Van Heurck 1885 | species |
| Phytoplankton | Nitzschia obtusa W.Smith 1853 | species |
| Phytoplankton | Nitzschia pacifica Cupp 1943 | species |
| Phytoplankton | Nitzschia palea (Kützing) W.Smith 1856 | species |
| Phytoplankton | Nitzschia paleacea (Grunow) Grunow 1881 | species |
| Phytoplankton | Nitzschia panduriformis var. minor Grunow 1880 | species |
| Phytoplankton | Nitzschia recta Hantzsch ex Rabenhorst 1862 | species |
| Phytoplankton | Nitzschia reversa f. parva (Grunow) Bukhtiyarova 1995 | species |
| Phytoplankton | Nitzschia sicula (Castracane) Hustedt 1958 | species |
| Phytoplankton | Nitzschia sigma (Kützing) W.Smith 1853 | species |
| Phytoplankton | Nitzschia sigma var. rigida Grunow ex Van Heurck 1880 | species |
| Phytoplankton | Nitzschia sigma var. sigmatella Grunow 1878 | species |
| Phytoplankton | Nitzschia tryblionella Hantzsch 1860 | species |
| Phytoplankton | Nitzschia valida Cleve & Grunow 1878 | species |
| Phytoplankton | Noctiluca scintillans (Macartney) Kofoid & Swezy 1921 | species |
| Phytoplankton | Odontella aurita (Lyngbye) C.Agardh 1832 | species |
| Phytoplankton | Odontella longicruris (Greville) M.A.Hoban 1983 | species |
| Phytoplankton | Odontella obtusa Kützing 1844 | species |
| Phytoplankton | Odontella regia (M.Schultze) Simonsen 1974 | species |
| Phytoplankton | Odontella rhombus (Ehrenberg) Kützing 1849 | species |
| Phytoplankton | Odontella turgida (Ehrenberg) Kützing 1844 | species |
| Phytoplankton | Opephora marina (W.Gregory) Petit 1888 | species |
| Phytoplankton | Opephora pacifica (Grunow) Petit 1888 | species |
| Phytoplankton | Oscillatoria limosa C.Agardh ex Gomont 1892 | species |
| Phytoplankton | Oscillatoria princeps Vaucher ex Gomont 1892 | species |
| Phytoplankton | Palmerina hardmaniana (Greville) G.R.Hasle 1996 | species |
| Phytoplankton | Paralia sulcata (Ehrenberg) Cleve 1873 | species |
| Phytoplankton | Pediastrum duplex Meyen 1829 | species |
| Phytoplankton | Peridinium gatunense Nygaard 1925 | species |
| Phytoplankton | Peridinium lenticulatum Fauré-Fremiet 1908 | species |
| Phytoplankton | Petrodictyon gemma (Ehrenberg) D.G.Mann 1990 | species |
| Phytoplankton | Petroneis humerosa (Brébisson ex W.Smith) Stickle & D.G.Mann 1990 | species |
| Phytoplankton | Petroneis transfuga (Grunow ex Cleve) D.G.Mann 1990 | species |
| Phytoplankton | Phacus curvicauda Svirenko 1915 | species |
| Phytoplankton | Phacus longicauda (Ehrenberg) Dujardin 1841 | species |
| Phytoplankton | Phacus longicauda var. attenuatus (Pochmann) Huber-Pestalozzi 1955 | species |
| Phytoplankton | Phacus orbicularis Hübner 1886 | species |
| Phytoplankton | Phacus tortus (Lemmermann) Skvortsov 1928 | species |
| Phytoplankton | Phormidium Kützing ex Gomont, 1892 | species |
| Phytoplankton | Phormidium papyraceum Gomont 1892 | species |
| Phytoplankton | Pinnularia nobilis (Ehrenberg) Ehrenberg 1843 | species |
| Phytoplankton | Pinnularia viridis (Nitzsch) Ehrenberg 1843 | species |
| Phytoplankton | Plagiogramma minus (W.Gregory) Chunlian Li, Ashworth & Witkowski 2020 | species |
| Phytoplankton | Plagiogramma pulchellum Greville 1859 | species |
| Phytoplankton | Plagiotropis seriata (Cleve) Kuntze 1898 | species |
| Phytoplankton | Planktothrix agardhii (Gomont) Anagnostidis & Komárek 1988 | species |
| Phytoplankton | Pleurosigma angulatum (J.T.Quekett) W.Smith 1852 | species |
| Phytoplankton | Pleurosigma elongatum W.Smith 1852 | species |
| Phytoplankton | Pleurosigma formosum W.Smith 1852 | species |
| Phytoplankton | Pleurosigma normanii Ralfs 1861 | species |
| Phytoplankton | Pleurosira laevis (Ehrenberg) Compère 1982 | species |
| Phytoplankton | Podocystis adriatica (Kützing) Ralfs 1861 | species |
| Phytoplankton | Podosira stelligera (Bailey) A.Mann 1907 | species |
| Phytoplankton | Proboscia alata (Brightwell) Sundström 1986 | species |
| Phytoplankton | Proboscia indica (H.Peragallo) Hernández-Becerril 1995 | species |
| Phytoplankton | Progonoia musca (W.Gregory) H.-J.Schrader 1974 | species |
| Phytoplankton | Prorocentrum gracile F.Schütt 1895 | species |
| Phytoplankton | Prorocentrum micans Ehrenberg 1834 | species |
| Phytoplankton | Protoperidinium brevipes (Paulsen) Balech 1974 | species |
| Phytoplankton | Protoperidinium claudicans (Paulsen) Balech 1974 | species |
| Phytoplankton | Protoperidinium conicoides (Paulsen) Balech 1973 | species |
| Phytoplankton | Protoperidinium conicum (Gran) Balech 1974 | species |
| Phytoplankton | Protoperidinium crassipes (Kofoid) Balech 1974 | species |
| Phytoplankton | Protoperidinium divergens (Ehrenberg) Balech 1974 | species |
| Phytoplankton | Protoperidinium leonis (Pavillard) Balech 1974 | species |
| Phytoplankton | Protoperidinium pentagonum (Gran) Balech 1974 | species |
| Phytoplankton | Protoperidinium punctulatum (Paulsen) Balech 1974 | species |
| Phytoplankton | Protoperidinium pyriforme (Paulsen) Balech 1974 | species |
| Phytoplankton | Protoperidinium steinii (Jørgensen) Balech 1974 | species |
| Phytoplankton | Psammodictyon constrictum (W.Gregory) D.G.Mann 1990 | species |
| Phytoplankton | Psammodictyon panduriforme (W.Gregory) D.G.Mann 1990 | species |
| Phytoplankton | Pseudictyota dubia (Brightwell) P.A.Sims & D.M.Williams 2018 | species |
| Phytoplankton | Pseudo-nitzschia pungens (Grunow ex Cleve) Hasle 1993 | species |
| Phytoplankton | Pseudo-nitzschia pungens var. atlantica (Cleve) Moreno & Licea 1996 | species |
| Phytoplankton | Pseudo-nitzschia seriata (Cleve) H.Peragallo 1899 | species |
| Phytoplankton | Pseudosolenia calcar-avis (Schultze) B.G.Sundström 1986 | species |
| Phytoplankton | Pyrophacus steinii (Schiller) Wall & Dale 1971 | species |
| Phytoplankton | Pyxidicula mediterranea Grunow 1882 | species |
| Phytoplankton | Ralfsiella smithii (Ralfs) P.A.Sims, D.M.Williams & Ashworth 2018 | species |
| Phytoplankton | Rhabdonema adriaticum Kützing 1844 | species |
| Phytoplankton | Rhaphoneis amphiceros (Ehrenberg) Ehrenberg 1844 | species |
| Phytoplankton | Rhizosolenia acicularis B.G.Sundström 1986 | species |
| Phytoplankton | Rhizosolenia acuminata (H.Peragallo) H.Peragallo 1907 | species |
| Phytoplankton | Rhizosolenia hebetata J.W.Bailey 1856 | species |
| Phytoplankton | Rhizosolenia imbricata Brightwell 1858 | species |
| Phytoplankton | Rhizosolenia setigera Brightwell 1858 | species |
| Phytoplankton | Rhizosolenia styliformis T.Brightwell 1858 | species |
| Phytoplankton | Rhopalodia gibberula (Ehrenberg) O.Müller 1895 | species |
| Phytoplankton | Rhopalodia musculus (Kützing) O.Müller 1900 | species |
| Phytoplankton | Romeria victoriae Komárek & Cronberg 2001 | species |
| Phytoplankton | Scenedesmus quadricauda (Turpin) Brébisson 1835 | species |
| Phytoplankton | Scenedesmus quadricauda var. quadrispina (Chodat) G.M.Smith 1916 | species |
| Phytoplankton | Shionodiscus oestrupii (Ostenfeld) A.J.Alverson, S.-H.Kang & E.C.Theriot 2006 | species |
| Phytoplankton | Skeletonema costatum (Greville) Cleve 1873 | species |
| Phytoplankton | Skeletonema tropicum Cleve 1900 | species |
| Phytoplankton | Staurastrum leptocladum Nordstedt 1870 | species |
| Phytoplankton | Strombomonas Deflandre, 1930 | species |
| Phytoplankton | Surirella minuta Brébisson ex Kützing 1849 | species |
| Phytoplankton | Surirella febigeri F.W.Lewis 1861 | species |
| Phytoplankton | Surirella fluminensis Grunow 1862 | species |
| Phytoplankton | Surirella minuta Brébisson ex Kützing, nom. illeg. 1849 | species |
| Phytoplankton | Surirella robusta Ehrenberg 1841 | species |
| Phytoplankton | Synedra ulna (Nitzsch) Ehrenberg 1832 | species |
| Phytoplankton | Tabularia affinis (Kützing) Snoeijs 1992 | species |
| Phytoplankton | Tabularia fasciculata (C.Agardh) D.M.Williams & Round 1986 | species |
| Phytoplankton | Tabularia tabulata (C.Agardh) Snoeijs 1992 | species |
| Phytoplankton | Terpsinoë americana (Bailey) Ralfs 1861 | species |
| Phytoplankton | Terpsinoë musica Ehrenberg 1843 | species |
| Phytoplankton | Thalassionema frauenfeldii (Grunow) Tempère & Peragallo 1910 | species |
| Phytoplankton | Thalassionema nitzschioides (Grunow) Mereschkowsky 1902 | species |
| Phytoplankton | Thalassionema nitzschioides var. capitulatum (H.J.Schrader) J.L.Moreno-Ruíz 1995 | species |
| Phytoplankton | Thalassionema nitzschioides var. claviforme (H.J.Schrader) J.L.Moreno-Ruiz 1993 | species |
| Phytoplankton | Thalassionema nitzschioides var. lanceolatum (Grunow) Heiden 1928 | species |
| Phytoplankton | Thalassiosira striata Harwood & Maruyama, 1992 | species |
| Phytoplankton | Thalassiosira eccentrica (Ehrenberg) Cleve 1904 | species |
| Phytoplankton | Thalassiosira gravida Cleve 1896 | species |
| Phytoplankton | Thalassiosira leptopus (Grunow) Hasle & G.Fryxell 1977 | species |
| Phytoplankton | Thalassiosira lineata Jousé 1968 | species |
| Phytoplankton | Thalassiosira nanolineata (A.Mann) Fryxell & Hasle 1977 | species |
| Phytoplankton | Thalassiosira oestrupii (Ostenfeld) Proshkina-Lavrenko ex Hasle 1960 | species |
| Phytoplankton | Thalassiosira simonsenii Hasle & G.Fryxell 1977 | species |
| Phytoplankton | Thalassiosira subtilis (Ostenfeld) Gran 1900 | species |
| Phytoplankton | Thalassiothrix longissima Cleve & Grunow 1880 | species |
| Phytoplankton | Trachelomonas armata (Ehrenberg) F.Stein 1878 | species |
| Phytoplankton | Trachyneis aspera (Ehrenberg) Cleve 1894 | species |
| Phytoplankton | Triadinium orientale (Lindemann) J.D.Dodge 1981 | species |
| Phytoplankton | Triceratium excavatum (Heiberg) Strelnikova, 1974 | species |
| Phytoplankton | Triceratium broeckii Leuduger-Fortmorel 1879 | species |
| Phytoplankton | Triceratium contortum Shadbolt 1854 | species |
| Phytoplankton | Triceratium favus Ehrenberg 1839 | species |
| Phytoplankton | Triceratium favus f. quadratum (Grunow) Hustedt 1930 | species |
| Phytoplankton | Triceratium reticulum Ehrenberg 1844 | species |
| Phytoplankton | Triceratium robertsianum Greville 1863 | species |
| Phytoplankton | Trieres mobiliensis (Bailey) Ashworth & E.C.Theriot 2013 | species |
| Phytoplankton | Trieres chinensis (Greville) Ashworth & E.C.Theriot 2013 | species |
| Phytoplankton | Trieres mobiliensis (Bailey) Ashworth & E.C.Theriot 2013 | species |
| Phytoplankton | Trieres regia (M.Schultze) Ashworth & E.C.Theriot 2013 | species |
| Phytoplankton | Trinacria excavata Heiberg, 1863 | species |
| Phytoplankton | Tripos furca (Ehrenberg) F.Gómez 2013 | species |
| Phytoplankton | Tripos fusus (Ehrenberg) F.Gómez 2013 | species |
| Phytoplankton | Tripos lineatus (Ehrenberg) F.Gómez 2021 | species |
| Phytoplankton | Tripos longipes (Bailey) F.Gómez 2021 | species |
| Phytoplankton | Tripos macroceros (Ehrenberg) Hallegraeff & Huisman 2020 | species |
| Phytoplankton | Tripos massiliensis (Gourret) F.Gómez 2021 | species |
| Phytoplankton | Tripos muelleri Bory 1826 | species |
| Phytoplankton | Tripos pentagonus (Gourret) F.Gómez 2021 | species |
| Phytoplankton | Tripos teres (Kofoid) F.Gómez 2013 | species |
| Phytoplankton | Tripos trichoceros (Ehrenberg) Gómez 2013 | species |
| Phytoplankton | Tryblionella angustata W.Smith 1853 | species |
| Phytoplankton | Tryblionella coarctata (Grunow) D.G.Mann 1990 | species |
| Phytoplankton | Tryblionella compressa (Bailey) Poulin 1990 | species |
| Phytoplankton | Tryblionella granulata (Grunow) D.G.Mann 1990 | species |
| Phytoplankton | Tryblionella hantzschiana Grunow 1862 | species |
| Phytoplankton | Tryblionella hyalina (Amossé) T.Ohtsuka 2005 | species |
| Phytoplankton | Tryblionella punctata W.Smith 1853 | species |
| Phytoplankton | Tryblionella victoriae Grunow 1862 | species |
| Phytoplankton | Tryblioptychus cocconeiformis (Grunow) Hendey 1958 | species |
| Phytoplankton | Ulnaria delicatissima (W.Smith) Aboal & P.C.Silva 2004 | species |
| Phytoplankton | Ulnaria ulna (Nitzsch) Compère 2001 | species |
| Phytoplankton | Zygoceros ehrenbergii E.A.Sar 2016 | species |
| Phytoplankton | Zygoceros rhombus Ehrenberg 1839 | species |
| Reptilia | Amerotyphlops (Hedges, Marion, Lipp, Marin & Vidal, 2014) | genus |
| Reptilia | Cnemidophorus Wagler, 1830 | genus |
| Reptilia | Liophis sp.1 Dixon JR (1980) | genus |
| Reptilia | Liophis sp.2 Dixon JR (1980) | genus |
| Reptilia | Mabuya Fitzinger, 1826 | genus |
| Reptilia | Ameiva ameiva (LINNAEUS, 1758) | species |
| Reptilia | Ameivula ocellifera (SPIX, 1825) | species |
| Reptilia | Amerotyphlops brongersmianus (VANZOLINI, 1976) | species |
| Reptilia | Amerotyphlops reticulatus (LINNAEUS, 1758) | species |
| Reptilia | Amphisbaena alba LINNAEUS, 1758 | species |
| Reptilia | Amphisbaena fuliginosa LINNAEUS, 1758 | species |
| Reptilia | Amphisbaena mitchelli PROCTER, 1923 | species |
| Reptilia | Amphisbaena vermicularis WAGLER, 1824 | species |
| Reptilia | Anilius scytale (LINNAEUS, 1758) | species |
| Reptilia | Anolis brasiliensis VANZOLINI & WILLIAMS, 1970 | species |
| Reptilia | Anolis chrysolepis DUMÉRIL & BIBRON, 1837 | species |
| Reptilia | Anolis fuscoauratus D’ORBIGNY, 1837 | species |
| Reptilia | Anolis ortonii COPE, 1868 | species |
| Reptilia | Anolis punctatus DAUDIN, 1802 | species |
| Reptilia | Apostolepis longicaudata GOMES, 1921 | species |
| Reptilia | Arthrosaura reticulata (O’SHAUGHNESSY, 1881) | species |
| Reptilia | Aspronema dorsivittatum (COPE, 1862) | species |
| Reptilia | Boa constrictor LINNAEUS, 1758 | species |
| Reptilia | Bothrops atrox (LINNAEUS, 1758) | species |
| Reptilia | Caiman crocodilus (LINNAEUS, 1758) | species |
| Reptilia | Caretta caretta (LINNAEUS, 1758) | species |
| Reptilia | Cercosaura olivacea (GRAY, 1845) | species |
| Reptilia | Chatogekko amazonicus (ANDERSSON, 1918) | species |
| Reptilia | Chelonia mydas (LINNAEUS, 1758) | species |
| Reptilia | Chironius bicarinatus (WIED-NEUWIED, 1820) | species |
| Reptilia | Chironius carinatus (LINNAEUS, 1758) | species |
| Reptilia | Chironius exoletus (LINNAEUS, 1758) | species |
| Reptilia | Chlorosoma viridissimum (LINNAEUS, 1758) | species |
| Reptilia | Clelia clelia (DAUDIN, 1803) | species |
| Reptilia | Coleodactylus meridionalis (BOULENGER, 1888) | species |
| Reptilia | Colobosaura modesta (REINHARDT & LÜTKEN, 1862) | species |
| Reptilia | Copeoglossum nigropunctatum (SPIX, 1825) | species |
| Reptilia | Corallus batesii (GRAY, 1860) | species |
| Reptilia | Corallus hortulana (LINNAEUS, 1758) | species |
| Reptilia | Crotalus durissus LINNAEUS, 1758 | species |
| Reptilia | Dermochelys coriacea (VANDELLI, 1761) | species |
| Reptilia | Dipsas catesbyi (SENTZEN, 1796) | species |
| Reptilia | Dipsas mikanii SCHLEGEL, 1837 | species |
| Reptilia | Drymarchon corais (BOIE, 1827) | species |
| Reptilia | Epicrates cenchria (LINNAEUS, 1758) | species |
| Reptilia | Eretmochelys imbricata (LINNAEUS, 1766) | species |
| Reptilia | Erythrolamprus cobella (LINNAEUS, 1758) | species |
| Reptilia | Erythrolamprus poecilogyrus (WIED-NEUWIED, 1824) | species |
| Reptilia | Erythrolamprus reginae (LINNAEUS, 1758) | species |
| Reptilia | Erythrolamprus taeniogaster (JAN, 1863) | species |
| Reptilia | Erythrolamprus viridis (GÜNTHER, 1862) | species |
| Reptilia | Eunectes murinus (LINNAEUS, 1758) | species |
| Reptilia | Gonatodes humeralis (GUICHENOT, 1855) | species |
| Reptilia | Helicops angulatus (LINNAEUS, 1758) | species |
| Reptilia | Hemidactylus mabouia (MOREAU DE JONNÈS, 1818) | species |
| Reptilia | Hoplocercus spinosus FITZINGER, 1843 | species |
| Reptilia | Hydrodynastes gigas (DUMÉRIL, BIBRON & DUMÉRIL, 1854) | species |
| Reptilia | Hydrops triangularis (WAGLER, 1824) | species |
| Reptilia | Iguana iguana (LINNAEUS, 1758) | species |
| Reptilia | Imantodes cenchoa (LINNAEUS, 1758) | species |
| Reptilia | Kentropyx calcarata SPIX, 1825 | species |
| Reptilia | Kinosternon scorpioides (LINNAEUS, 1766) | species |
| Reptilia | Lepidochelys olivacea (ESCHSCHOLTZ, 1829) | species |
| Reptilia | Leposternon polystegum (DUMÉRIL, 1851) | species |
| Reptilia | Leptodeira annulata (LINNAEUS, 1758) | species |
| Reptilia | Leptophis ahaetulla (LINNAEUS, 1758) | species |
| Reptilia | Manciola guaporicola (DUNN, 1935) | species |
| Reptilia | Mastigodryas boddaerti (SENTZEN, 1796) | species |
| Reptilia | Micrablepharus maximiliani (REINHARDT & LÜTKEN, 1862) | species |
| Reptilia | Micrurus lemniscatus (LINNAEUS, 1758) | species |
| Reptilia | Micrurus spixii WAGLER, 1824 | species |
| Reptilia | Micrurus surinamensis (CUVIER, 1816) | species |
| Reptilia | Oxybelis aeneus (WAGLER, 1824) | species |
| Reptilia | Oxybelis fulgidus (DAUDIN, 1803) | species |
| Reptilia | Oxyrhopus guibei HOGE & ROMANO, 1977 | species |
| Reptilia | Oxyrhopus petolarius (LINNAEUS, 1758) | species |
| Reptilia | Paleosuchus palpebrosus (CUVIER, 1807) | species |
| Reptilia | Palusophis bifossatus (RADDI, 1820) | species |
| Reptilia | Philodryas olfersii (LICHTENSTEIN, 1823) | species |
| Reptilia | Phrynops geoffroanus (SCHWEIGGER, 1812) | species |
| Reptilia | Platemys platycephala (SCHNEIDER, 1792) | species |
| Reptilia | Polychrus marmoratus (LINNAEUS, 1758) | species |
| Reptilia | Pseudoboa nigra (DUMÉRIL, BIBRON & DUMÉRIL, 1854) | species |
| Reptilia | Pseudoeryx plicatilis (LINNAEUS, 1758) | species |
| Reptilia | Psomophis joberti (SAUVAGE, 1884) | species |
| Reptilia | Rhinoclemmys punctularia (DAUDIN, 1801) | species |
| Reptilia | Sibon nebulatus (LINNAEUS, 1758) | species |
| Reptilia | Siphlophis cervinus (LAURENTI, 1768) | species |
| Reptilia | Siphlophis compressus (DAUDIN, 1803) | species |
| Reptilia | Spilotes pullatus (LINNAEUS, 1758) | species |
| Reptilia | Tantilla melanocephala (LINNAEUS, 1758) | species |
| Reptilia | Thamnodynastes pallidus (LINNAEUS, 1758) | species |
| Reptilia | Thecadactylus rapicauda (HOUTTUYN, 1782) | species |
| Reptilia | Trachemys adiutrix (VANZOLINI, 1995) | species |
| Reptilia | Tropidurus hispidus (SPIX, 1825) | species |
| Reptilia | Tupinambis teguixin (LINNAEUS, 1758) | species |
| Reptilia | Uranoscodon superciliosus (Linnaeus, 1758) | species |
| Reptilia | Xenodon rabdocephalus (WIED-NEUWIED, 1824) | species |
| Reptilia | Xenoxybelis argenteus (DAUDIN, 1803) | species |
| Vegetation | Acanthaceae Juss. | family |
| Vegetation | Aizoaceae Martinov | family |
| Vegetation | Anacardiaceae R.Br. | family |
| Vegetation | Annonaceae Juss. | family |
| Vegetation | Araceae Juss. | family |
| Vegetation | Asteraceae Bercht. & J.Presl | family |
| Vegetation | Celastraceae R.Br. | family |
| Vegetation | Chrysobalanaceae R.Br. | family |
| Vegetation | Convolvulaceae Juss. | family |
| Vegetation | Cyperaceae Juss. | family |
| Vegetation | Euphorbiaceae Juss. | family |
| Vegetation | Fabaceae Lindl. | family |
| Vegetation | Gentianaceae Juss. | family |
| Vegetation | Lauraceae Juss. | family |
| Vegetation | Lecythidaceae A.Rich. | family |
| Vegetation | Malpighiaceae Juss. | family |
| Vegetation | Melastomataceae A.Juss. | family |
| Vegetation | Meliaceae A.Juss. | family |
| Vegetation | Moraceae Gaudich. | family |
| Vegetation | Myrtaceae Juss. | family |
| Vegetation | Nyctaginaceae Juss. | family |
| Vegetation | Poaceae Barnhart | family |
| Vegetation | Portulacaceae Juss. | family |
| Vegetation | Rubiaceae Juss. | family |
| Vegetation | Rutaceae A.Juss. | family |
| Vegetation | Salicaceae Mirb. | family |
| Vegetation | Sapindaceae Juss. | family |
| Vegetation | Sapotaceae Juss. | family |
| Vegetation | Simaroubaceae DC. | family |
| Vegetation | Verbenaceae J.St.-Hil. | family |
| Vegetation | Acacia Mill. | genus |
| Vegetation | Aechmea Ruiz & Pav. | genus |
| Vegetation | Albizia Durazz. | genus |
| Vegetation | Alchornea Sw. | genus |
| Vegetation | Alibertia A.Rich. ex DC. | genus |
| Vegetation | Allamanda L. | genus |
| Vegetation | Alternanthera Forssk. | genus |
| Vegetation | Amaranthus L. | genus |
| Vegetation | Amasonia L.f. | genus |
| Vegetation | Ananas Mill. | genus |
| Vegetation | Anaxagorea A.St.-Hil. | genus |
| Vegetation | Andira Lam. | genus |
| Vegetation | Anemopaegma Mart. ex Meisn. | genus |
| Vegetation | Aniba Aubl. | genus |
| Vegetation | Annona L. | genus |
| Vegetation | Apeiba Aubl. | genus |
| Vegetation | Apuleia Mart. | genus |
| Vegetation | Aristolochia L. | genus |
| Vegetation | Aspidosperma Mart. & Zucc. | genus |
| Vegetation | Astrocaryum G.Mey. | genus |
| Vegetation | Avicennia L. | genus |
| Vegetation | Axonopus P. Beauv. | genus |
| Vegetation | Azolla Lam. | genus |
| Vegetation | Bacopa Aubl. | genus |
| Vegetation | Bactris Jacq. ex Scop. | genus |
| Vegetation | Banara Aubl. | genus |
| Vegetation | Bauhinia L. | genus |
| Vegetation | Bignonia L. | genus |
| Vegetation | Blepharodon Decne. | genus |
| Vegetation | Bombax L. | genus |
| Vegetation | Bonamia Thouars | genus |
| Vegetation | Bougainvillea Comm. ex Juss. | genus |
| Vegetation | Bredemeyera Willd. | genus |
| Vegetation | Bromelia L. | genus |
| Vegetation | Bulbostylis Kunth | genus |
| Vegetation | Byrsonima Rich. ex Kunth | genus |
| Vegetation | Campomanesia Ruiz et Pav. | genus |
| Vegetation | Casearia Jacq. | genus |
| Vegetation | Cassia L. | genus |
| Vegetation | Cecropia Loefl. | genus |
| Vegetation | Ceiba Mill. | genus |
| Vegetation | Celtis L. | genus |
| Vegetation | Cenchrus L. | genus |
| Vegetation | Centrolobium Mart. ex Benth. | genus |
| Vegetation | Cereus Mill. | genus |
| Vegetation | Cestrum L. | genus |
| Vegetation | Chomelia Jacq. | genus |
| Vegetation | Chrysophyllum L. | genus |
| Vegetation | Cissus L. | genus |
| Vegetation | Clitoria L. | genus |
| Vegetation | Clusia L. | genus |
| Vegetation | Cnidoscolus Pohl | genus |
| Vegetation | Coccoloba P.Browne | genus |
| Vegetation | Cochlospermum Kunth | genus |
| Vegetation | Commelina L. | genus |
| Vegetation | Connarus L. | genus |
| Vegetation | Copaifera L. | genus |
| Vegetation | Cordia L. | genus |
| Vegetation | Cordiera A.Rich. ex DC. | genus |
| Vegetation | Cosmos Cav. | genus |
| Vegetation | Costus L. | genus |
| Vegetation | Crenea Aubl. | genus |
| Vegetation | Crinum L. | genus |
| Vegetation | Crotalaria L. | genus |
| Vegetation | Croton L. | genus |
| Vegetation | Cucurbita L. | genus |
| Vegetation | Cupania L. | genus |
| Vegetation | Cuphea P.Browne | genus |
| Vegetation | Cybianthus Mart. | genus |
| Vegetation | Cyperus L. | genus |
| Vegetation | Dalbergia L.f. | genus |
| Vegetation | Dendropanax Decne. & Planch. | genus |
| Vegetation | Derris Lour. | genus |
| Vegetation | Desmoncus Mart. | genus |
| Vegetation | Didymopanax Decne. & Planch. | genus |
| Vegetation | Digitaria Haller | genus |
| Vegetation | Dimorphandra Schott | genus |
| Vegetation | Dinizia Ducke | genus |
| Vegetation | Diodia L. | genus |
| Vegetation | Dioscorea L. | genus |
| Vegetation | Duguetia A.St.-Hil. | genus |
| Vegetation | Dulacia Vell. | genus |
| Vegetation | Echinochloa P.Beauv. | genus |
| Vegetation | Eleocharis R.Br. | genus |
| Vegetation | Emilia Cass. | genus |
| Vegetation | Ephedranthus S.Moore | genus |
| Vegetation | Eragrostis Wolf | genus |
| Vegetation | Eriochloa Kunth | genus |
| Vegetation | Eriotheca Schott & Endl. | genus |
| Vegetation | Erythrina L. | genus |
| Vegetation | Erythroxylum P.Browne | genus |
| Vegetation | Eschweilera Mart. ex DC. | genus |
| Vegetation | Eucalyptus L'Hér. | genus |
| Vegetation | Eugenia L. | genus |
| Vegetation | Eupatorium L. | genus |
| Vegetation | Faramea Aubl. | genus |
| Vegetation | Ficus L. | genus |
| Vegetation | Fimbristylis Vahl | genus |
| Vegetation | Fridericia Mart. | genus |
| Vegetation | Galeandra Lindl. | genus |
| Vegetation | Garcinia L. | genus |
| Vegetation | Griffinia Ker Gawl. | genus |
| Vegetation | Guazuma Adans. | genus |
| Vegetation | Guettarda L. | genus |
| Vegetation | Helicteres L. | genus |
| Vegetation | Heliotropium L. | genus |
| Vegetation | Heteropterys Kunth | genus |
| Vegetation | Hexasepalum Bartl. ex DC. | genus |
| Vegetation | Hibiscus L. | genus |
| Vegetation | Hieronyma Allemão | genus |
| Vegetation | Himatanthus Willdenow | genus |
| Vegetation | Hirtella L. | genus |
| Vegetation | Hymenaea L. | genus |
| Vegetation | Ilex L. | genus |
| Vegetation | Inga Mill. | genus |
| Vegetation | Ipomoea L. | genus |
| Vegetation | Lacistema Sw. | genus |
| Vegetation | Lafoensia Vand. | genus |
| Vegetation | Lamium L. | genus |
| Vegetation | Lantana L. | genus |
| Vegetation | Lecythis Loefl. | genus |
| Vegetation | Leucaena Benth. | genus |
| Vegetation | Licania Aubl. | genus |
| Vegetation | Lippia L. | genus |
| Vegetation | Lonchocarpus Kunth | genus |
| Vegetation | Ludwigia L. | genus |
| Vegetation | Mabea Aubl. | genus |
| Vegetation | Machaerium Pers. | genus |
| Vegetation | Maclura Nutt. | genus |
| Vegetation | Macroptilium (Benth.) Urb. | genus |
| Vegetation | Malva L. | genus |
| Vegetation | Mandevilla Lindl. | genus |
| Vegetation | Mandevilla Lindl. | genus |
| Vegetation | Manilkara Adans. | genus |
| Vegetation | Margaritaria L.f. | genus |
| Vegetation | Marlierea Cambess. | genus |
| Vegetation | Marsilea L. | genus |
| Vegetation | Matayba Aubl. | genus |
| Vegetation | Maytenus Molina | genus |
| Vegetation | Mendoncia Vell. ex Vand. | genus |
| Vegetation | Merremia Dennst. ex Endl. | genus |
| Vegetation | Miconia Ruiz & Pav. | genus |
| Vegetation | Micropholis (Griseb.) Pierre | genus |
| Vegetation | Mimosa L. | genus |
| Vegetation | Musa L. | genus |
| Vegetation | Myrcia DC. | genus |
| Vegetation | Myrciaria O.Berg | genus |
| Vegetation | Nectandra Rol. ex Rottb. | genus |
| Vegetation | Nephrolepis Schott | genus |
| Vegetation | Neptunia Lour. | genus |
| Vegetation | Ocotea Aubl. | genus |
| Vegetation | Olyra L. | genus |
| Vegetation | Ormosia Jacks. | genus |
| Vegetation | Ouratea Aubl. | genus |
| Vegetation | Oxypetalum R.Br. | genus |
| Vegetation | Pagamea Aubl. | genus |
| Vegetation | Palicourea Aubl. | genus |
| Vegetation | Panicum L. | genus |
| Vegetation | Parahancornia Ducke | genus |
| Vegetation | Parkia R.Br. | genus |
| Vegetation | Paspalum L. | genus |
| Vegetation | Passiflora L. | genus |
| Vegetation | Paullinia L. | genus |
| Vegetation | Pavonia Cav. | genus |
| Vegetation | Phaseolus L. | genus |
| Vegetation | Philodendron Schott | genus |
| Vegetation | Phyllanthus L. | genus |
| Vegetation | Pilocarpus Vahl | genus |
| Vegetation | Piper L. | genus |
| Vegetation | Pithecellobium Mart. | genus |
| Vegetation | Polygala L. | genus |
| Vegetation | Polygonum L. | genus |
| Vegetation | Posoqueria Aubl. | genus |
| Vegetation | Pouteria Aubl. | genus |
| Vegetation | Prosopis L. | genus |
| Vegetation | Protium Burm.f. | genus |
| Vegetation | Psidium L. | genus |
| Vegetation | Psittacanthus Mart. | genus |
| Vegetation | Psychotria L. | genus |
| Vegetation | Pterolepis (DC.) Miq. | genus |
| Vegetation | Qualea Aubl. | genus |
| Vegetation | Quararibea Aubl. | genus |
| Vegetation | Rhynchanthera DC. | genus |
| Vegetation | Rhynchospora Vahl | genus |
| Vegetation | Rinorea Aubl. | genus |
| Vegetation | Rondeletia L. | genus |
| Vegetation | Roupala Aubl. | genus |
| Vegetation | Rourea Aubl. | genus |
| Vegetation | Ruellia L. | genus |
| Vegetation | Sapindus L. | genus |
| Vegetation | Sauvagesia L. | genus |
| Vegetation | Schefflera J.R.Forst. & G.Forst. | genus |
| Vegetation | Schultesia Mart. | genus |
| Vegetation | Scleria P.J.Bergius | genus |
| Vegetation | Senna Mill. | genus |
| Vegetation | Serjania Mill. | genus |
| Vegetation | Sida L. | genus |
| Vegetation | Sideroxylon L. | genus |
| Vegetation | Simaba Aubl. | genus |
| Vegetation | Simarouba Aubl. | genus |
| Vegetation | Sloanea L. | genus |
| Vegetation | Smilax L. | genus |
| Vegetation | Solanum L. | genus |
| Vegetation | Spermacoce L. | genus |
| Vegetation | Spondias L. | genus |
| Vegetation | Stachys L. | genus |
| Vegetation | Sterculia L. | genus |
| Vegetation | Stigmaphyllon A.Juss. | genus |
| Vegetation | Stryphnodendron Mart. | genus |
| Vegetation | Syagrus Mart. | genus |
| Vegetation | Tabebuia Gomes ex DC. | genus |
| Vegetation | Tabernaemontana L. | genus |
| Vegetation | Tachigali Aubl. | genus |
| Vegetation | Talisia Aubl. | genus |
| Vegetation | Tapirira Aubl. | genus |
| Vegetation | Terminalia L. | genus |
| Vegetation | Tetrapterys Cav. | genus |
| Vegetation | Theobroma L. | genus |
| Vegetation | Tibouchina Aubl. | genus |
| Vegetation | Tococa Aubl. | genus |
| Vegetation | Tocoyena Aubl. | genus |
| Vegetation | Trattinnickia Willd. | genus |
| Vegetation | Triplaris L. | genus |
| Vegetation | Triumfetta L. | genus |
| Vegetation | Turnera L. | genus |
| Vegetation | Typha L. | genus |
| Vegetation | Urochloa P.Beauv. | genus |
| Vegetation | Utricularia L. | genus |
| Vegetation | Vatairea Aubl. | genus |
| Vegetation | Vernonia Schreb. | genus |
| Vegetation | Virola Aubl. | genus |
| Vegetation | Vismia Vand. | genus |
| Vegetation | Vitex L. | genus |
| Vegetation | Vochysia Aubl. | genus |
| Vegetation | Ximenia L. | genus |
| Vegetation | Xylopia L. | genus |
| Vegetation | Xyris Gronov. ex L. | genus |
| Vegetation | Zanthoxylum L. | genus |
| Vegetation | Zornia J.F.Gmel. | genus |
| Vegetation | Abarema campestris (Spruce ex Benth.) Barneby & J.W.Grimes | species |
| Vegetation | Abarema cochleata (Willd.) Barneby & J.W.Grimes | species |
| Vegetation | Abarema jupunba (Willd.) Britton & Killip | species |
| Vegetation | Abrus fruticulosus Wight & Arn. | species |
| Vegetation | Abrus precatorius L. | species |
| Vegetation | Acacia auriculiformis A.Cunn. ex Benth. | species |
| Vegetation | Acacia longifolia (Andrews) Willd. | species |
| Vegetation | Acacia mangium Willd. | species |
| Vegetation | Acanthospermum australe (Loefl.) Kuntze | species |
| Vegetation | Acanthospermum hispidum DC. | species |
| Vegetation | Achyrocline alata (Kunth) DC. | species |
| Vegetation | Aciotis annua (Mart. ex DC.) Triana | species |
| Vegetation | Acmella uliginosa (Sw.) Cass. | species |
| Vegetation | Acrocomia aculeata (Jacq.) Lodd. ex Mart. | species |
| Vegetation | Acrostichum aureum L. | species |
| Vegetation | Acrostichum danaeifolium Langsd. & Fisch. | species |
| Vegetation | Actinostemon concepcionis (Chodat & Hassl.) Hochr. | species |
| Vegetation | Actinostemon klotzschii (Didr.) Pax | species |
| Vegetation | Adenanthera pavonina L. | species |
| Vegetation | Adenocalymma allamandiflorum (Bureau ex K.Schum.) L.G.Lohmann | species |
| Vegetation | Adenocalymma comosum (Cham.) DC. | species |
| Vegetation | Adenocalymma scabriusculum Mart. ex DC. | species |
| Vegetation | Adenocalymma schomburgkii (DC.) L.G.Lohmann | species |
| Vegetation | Adenocalymma validum L.G.Lohmann | species |
| Vegetation | Adiantum argutum Splitg. | species |
| Vegetation | Adiantum deflectens Mart. | species |
| Vegetation | Adiantum dolosum Kunze | species |
| Vegetation | Adiantum petiolatum Desv. | species |
| Vegetation | Adiantum pulverulentum L. | species |
| Vegetation | Adiantum terminatum Kunze ex Miq. | species |
| Vegetation | Adonidia merrillii (Becc.) Becc. | species |
| Vegetation | Aechmea angustifolia Poepp. & Endl. | species |
| Vegetation | Aechmea castelnavii Baker | species |
| Vegetation | Aeschynomene denticulata Rudd | species |
| Vegetation | Aeschynomene evenia C.Wright & Sauvalle | species |
| Vegetation | Aeschynomene fluminensis Vell. | species |
| Vegetation | Aeschynomene rudis Benth. | species |
| Vegetation | Aeschynomene sensitiva Sw. | species |
| Vegetation | Agave americana L. | species |
| Vegetation | Agave angustifolia Haw. | species |
| Vegetation | Agave sisalana Perrine ex Engelm. | species |
| Vegetation | Ageratum conyzoides L. | species |
| Vegetation | Agonandra brasiliensis Miers ex Benth. & Hook.f. | species |
| Vegetation | Agonandra silvatica Ducke | species |
| Vegetation | Aiouea myristicoides Mez | species |
| Vegetation | Albizia lebbeck (L.) Benth. | species |
| Vegetation | Albizia pedicellaris (DC.) L.Rico | species |
| Vegetation | Albizia polycephala (Benth.) Killip ex Record | species |
| Vegetation | Alcantarea geniculata (Wawra) J.R.Grant | species |
| Vegetation | Alchornea discolor Poepp. | species |
| Vegetation | Alexa grandiflora  Ducke | species |
| Vegetation | Alibertia edulis (Rich.) A.Rich. | species |
| Vegetation | Allagoptera campestris (Mart.) Kuntze | species |
| Vegetation | Allamanda blanchetii A.DC. | species |
| Vegetation | Allamanda cathartica L. | species |
| Vegetation | Allamanda doniana Müll. Arg. | species |
| Vegetation | Allophylus edulis (A. St.-Hil., A. Juss. & Cambess.) Hieron. ex Niederl. | species |
| Vegetation | Alternanthera bettzichiana (Regel) G.Nicholson | species |
| Vegetation | Alternanthera brasiliana (L.) Kuntze | species |
| Vegetation | Alternanthera dentata (Moench) Stuchlík ex R.E. Fr. | species |
| Vegetation | Alternanthera littoralis P. Beauv. ex Moq. | species |
| Vegetation | Alternanthera paronychioides A.St.-Hil. | species |
| Vegetation | Alternanthera philoxeroides (Mart.) Griseb. | species |
| Vegetation | Alternanthera sessilis (L.) R. Br. ex DC. | species |
| Vegetation | Alternanthera tenella Colla | species |
| Vegetation | Alysicarpus vaginalis (L.) DC. | species |
| Vegetation | Amaioua guianensis Aubl. | species |
| Vegetation | Amanoa congesta W.J.Hayden | species |
| Vegetation | Amaranthus blitum L. | species |
| Vegetation | Amaranthus caudatus L. | species |
| Vegetation | Amaranthus deflexus L. | species |
| Vegetation | Amaranthus retroflexus L. | species |
| Vegetation | Amaranthus spinosus L. | species |
| Vegetation | Amaranthus viridis L. | species |
| Vegetation | Amaryllis belladonna L. | species |
| Vegetation | Amasonia arborea Kunth | species |
| Vegetation | Amasonia calycina Hook. f. | species |
| Vegetation | Amasonia campestris (Aubl.) Moldenke | species |
| Vegetation | Ambrosia cumanensis Kunth | species |
| Vegetation | Ambrosia microcephala DC. | species |
| Vegetation | Amphilophium racemosum (Bureau & K.Schum.) L.G.Lohmann | species |
| Vegetation | Anacardium giganteum W. Hancock ex Engl. | species |
| Vegetation | Anacardium humile A.St.-Hil. | species |
| Vegetation | Anacardium occidentale L. | species |
| Vegetation | Anadenanthera colubrina (Vell.) Brenan | species |
| Vegetation | Ananas bracteatus (Lindl.) Schult. & Schult.f. | species |
| Vegetation | Ananas comosus (L.) Merril | species |
| Vegetation | Anaxagorea brevipes Benth. | species |
| Vegetation | Anaxagorea dolichocarpa Sprague & Sandwith | species |
| Vegetation | Anaxagorea phaeocarpa Mart. | species |
| Vegetation | Anaxagorea prinoides (Dunal) A. DC. | species |
| Vegetation | Ancistrotropis peduncularis (Fawc. & Rendle) A. Delgado | species |
| Vegetation | Andira cujabensis Benth. | species |
| Vegetation | Andira fraxinifolia Benth. | species |
| Vegetation | Andira legalis (Vell.) Toledo | species |
| Vegetation | Andira micrantha Ducke | species |
| Vegetation | Andira surinamensis (Bondt) Splitg. ex Amshoff | species |
| Vegetation | Andropogon bicornis L. | species |
| Vegetation | Andropogon leucostachyus Kunth | species |
| Vegetation | Anemopaegma chamberlaynii (Sims) Bureau & K.Schum. | species |
| Vegetation | Anemopaegma glaucum Mart. ex DC. | species |
| Vegetation | Anemopaegma parkeri Sprague | species |
| Vegetation | Aniba megaphylla Mez | species |
| Vegetation | Aniba panurensis (Meisn.) Mez | species |
| Vegetation | Aniba parviflora (Meisn.) Mez | species |
| Vegetation | Aniba terminalis Ducke | species |
| Vegetation | Aniseia cernua Moric. | species |
| Vegetation | Annona crassiflora Mart. | species |
| Vegetation | Annona densicoma Mart. | species |
| Vegetation | Annona dolabripetala Raddi | species |
| Vegetation | Annona exsucca DC. | species |
| Vegetation | Annona glabra L. | species |
| Vegetation | Annona montana Macfad. | species |
| Vegetation | Annona mucosa Jacq. | species |
| Vegetation | Annona muricata L. | species |
| Vegetation | Annona neoinsignis H. Rainer | species |
| Vegetation | Annona paludosa Aubl. | species |
| Vegetation | Annona reticulata L. | species |
| Vegetation | Annona squamosa L. | species |
| Vegetation | Annona sylvatica A. St.-Hil. | species |
| Vegetation | Annona tomentosa R.E.Fr. | species |
| Vegetation | Anthurium andraeanum Linden ex André | species |
| Vegetation | Anthurium sinuatum Benth. ex Schott | species |
| Vegetation | Apeiba echinata Gaertn. | species |
| Vegetation | Apeiba tibourbou Aubl. | species |
| Vegetation | Apteria aphylla (Nutt.) Barnhart ex Small | species |
| Vegetation | Apuleia leiocarpa (Vogel) J.F.Macbr. | species |
| Vegetation | Arachis pintoi Krapov. & W.C.Greg. | species |
| Vegetation | Araucaria columnaris (J.R.Forst.) Hook. | species |
| Vegetation | Arenga caudata (Lour.) H.E.Moore | species |
| Vegetation | Aristolochia chiquitensis Duch. | species |
| Vegetation | Aristolochia odoratissima L. | species |
| Vegetation | Artemisia vulgaris L. | species |
| Vegetation | Artocarpus altilis (Parkinson) Fosberg | species |
| Vegetation | Artocarpus heterophyllus Lam. | species |
| Vegetation | Artocarpus integrifolia L.f. | species |
| Vegetation | Artocarpus heterophyllus Lam. | species |
| Vegetation | Asclepias curassavica L. | species |
| Vegetation | Asemeia hirsuta (A.St.-Hil. & Moq.) J.F.B.Pastore & J.R.Abbott | species |
| Vegetation | Asemeia martiana (A.W.Benn.) J.F.B.Pastore & J.R.Abbott | species |
| Vegetation | Asemeia violacea (Aubl.) J.F.B.Pastore & J.R.Abbott | species |
| Vegetation | Aspidosperma album (Vahl) Benoist ex Pichon | species |
| Vegetation | Aspidosperma auriculatum Markgr. | species |
| Vegetation | Aspidosperma brasiliense A.S.S.Pereira & A.C.D.Castello | species |
| Vegetation | Aspidosperma centrale Markgr. | species |
| Vegetation | Aspidosperma desmanthum Benth. ex Müll.Arg. | species |
| Vegetation | Aspidosperma discolor A.DC. | species |
| Vegetation | Aspidosperma eteanum Markgr. | species |
| Vegetation | Aspidosperma excelsum Benth. | species |
| Vegetation | Aspidosperma marcgravianum Woodson | species |
| Vegetation | Aspidosperma nitidum Benth. ex Müll.Arg. | species |
| Vegetation | Aspidosperma sandwithianum Markgr. | species |
| Vegetation | Aspidosperma spruceanum Benth. ex Müll.Arg. | species |
| Vegetation | Aspidosperma verruculosum Müll.Arg. | species |
| Vegetation | Astraea lobata (L.) Klotzsch | species |
| Vegetation | Astraea surinamensis (Miq.) O.L.M. Silva & Cordeiro | species |
| Vegetation | Astrocaryum aculeatum G.Mey. | species |
| Vegetation | Astrocaryum jauari Mart. | species |
| Vegetation | Astrocaryum tucuma Mart. | species |
| Vegetation | Astrocaryum vulgare Mart. | species |
| Vegetation | Astronium fraxinifolium Schott | species |
| Vegetation | Astronium lecointei Ducke | species |
| Vegetation | Astronium urundeuva (M.Allemão) Engl. | species |
| Vegetation | Attalea dahlgreniana (Bondar) Wess.Boer | species |
| Vegetation | Attalea dubia (Mart.) Burret | species |
| Vegetation | Attalea maripa (Aubl.) Mart. | species |
| Vegetation | Attalea speciosa Mart. | species |
| Vegetation | Aucuba japonica Thunb. | species |
| Vegetation | Averrhoa bilimbi L. | species |
| Vegetation | Avicennia germinans (L.) L. | species |
| Vegetation | Avicennia schaueriana Stapf & Leechm. ex Moldenke | species |
| Vegetation | Axonopus pubivaginatus Henrard | species |
| Vegetation | Axonopus purpusii (Mez) Chase | species |
| Vegetation | Axonopus scoparius (Flüggé) Kuhlm. | species |
| Vegetation | Baccharis crispa Spreng. | species |
| Vegetation | Bactris acanthocarpa Mart. | species |
| Vegetation | Bactris brongniartii Mart. | species |
| Vegetation | Bactris gasipaes Kunth | species |
| Vegetation | Bactris maraja Mart. | species |
| Vegetation | Bactris setosa Mart. | species |
| Vegetation | Bagassa guianensis Aubl. | species |
| Vegetation | Balfourodendron riedelianum (Engl.) Engl. | species |
| Vegetation | Bambusa vulgaris Schrad. ex J.C.Wendl. | species |
| Vegetation | Banara arguta Briq. | species |
| Vegetation | Banara guianensis Aubl. | species |
| Vegetation | Banisteriopsis muricata (Cav.) Cuatrec. | species |
| Vegetation | Barleria cristata L. | species |
| Vegetation | Barrosoa apiculata (Gardner) R.M.King & H.Rob. | species |
| Vegetation | Basella alba L. | species |
| Vegetation | Bathysa australis (A.St.-Hil.) K.Schum. | species |
| Vegetation | Batis maritima L. | species |
| Vegetation | Bauhinia acreana Harms | species |
| Vegetation | Bauhinia dubia G.Don | species |
| Vegetation | Bauhinia longicuspis Benth. | species |
| Vegetation | Bauhinia longifolia (Bong.) Steud. | species |
| Vegetation | Bauhinia platypetala Burch. ex Benth. | species |
| Vegetation | Bauhinia smilacifolia Burch. ex Benth. | species |
| Vegetation | Bauhinia tenella Benth. | species |
| Vegetation | Bauhinia ungulata L. | species |
| Vegetation | Begonia cucullata Willd. | species |
| Vegetation | Bellis perennis L. | species |
| Vegetation | Bellucia grossularioides  (L.) Triana | species |
| Vegetation | Bidens bipinnata L. | species |
| Vegetation | Bidens pilosa L. | species |
| Vegetation | Bidens riparia Kunth | species |
| Vegetation | Bignonia aequinoctialis L. | species |
| Vegetation | Bignonia corymbosa (Vent.) L.G.Lohmann | species |
| Vegetation | Bixa arborea Huber | species |
| Vegetation | Bixa orellana L. | species |
| Vegetation | Blainvillea acmella (L.) Philipson | species |
| Vegetation | Blutaparon portulacoides (A.St.-Hil.) Mears | species |
| Vegetation | Blutaparon vermiculare (L.) Mears | species |
| Vegetation | Bocageopsis multiflora (Mart.) R.E.Fr. | species |
| Vegetation | Boehmeria nivea (L.) Gaudich. | species |
| Vegetation | Boerhavia diffusa L. | species |
| Vegetation | Bomarea edulis (Tussac) Herb. | species |
| Vegetation | Borreria capitata (Ruiz & Pav.) DC. | species |
| Vegetation | Borreria latifolia (Aubl.) K.Schum. | species |
| Vegetation | Borreria suaveolens G. Mey. | species |
| Vegetation | Borreria verticillata (L.) G.Mey. | species |
| Vegetation | Bougainvillea spectabilis Willd. | species |
| Vegetation | Bowdichia virgilioides Kunth | species |
| Vegetation | Bromelia balansae Mez | species |
| Vegetation | Bromelia laciniosa Mart. ex Schult. & Schult.f. | species |
| Vegetation | Brosimum guianense (Aubl.) Huber | species |
| Vegetation | Brosimum gaudichaudii Trécul | species |
| Vegetation | Buchnera nordestina Scatigna | species |
| Vegetation | Buchnera palustris (Aubl.) Spreng. | species |
| Vegetation | Bulbostylis capillaris (L.) C.B.Clarke | species |
| Vegetation | Bulbostylis capillaris (L.) Kunth ex C.B. Clarke | species |
| Vegetation | Bunchosia maritima (Vell.) J.F.Macbr. | species |
| Vegetation | Burmannia capitata (Walter ex J.F.Gmel.) Mart. | species |
| Vegetation | Byrsonima aerugo Sagot | species |
| Vegetation | Byrsonima chrysophylla Kunth | species |
| Vegetation | Byrsonima crassifolia (L.) Kunth | species |
| Vegetation | Byrsonima crispa  A.Juss. | species |
| Vegetation | Byrsonima cydoniifolia A.Juss. | species |
| Vegetation | Byrsonima laxiflora Griseb. | species |
| Vegetation | Byrsonima sericea DC. | species |
| Vegetation | Byrsonima spicata (Cav.) DC. | species |
| Vegetation | Byrsonima verbascifolia (L.) DC. | species |
| Vegetation | Caesalpinia pulcherrima (L.) Sw. | species |
| Vegetation | Cajanus cajan (L.) Huth | species |
| Vegetation | Calliandra brevipes Benth. | species |
| Vegetation | Calliandra virgata Benth. | species |
| Vegetation | Callisthene fasciculata Mart. | species |
| Vegetation | Callisthene minor Mart. | species |
| Vegetation | Calophyllum brasiliense Cambess. | species |
| Vegetation | Calopogonium caeruleum (Benth.) C.Wright | species |
| Vegetation | Calopogonium mucunoides Desv. | species |
| Vegetation | Calotropis procera (Aiton) W.T.Aiton | species |
| Vegetation | Calycolpus goetheanus (Mart. ex DC.) O.Berg | species |
| Vegetation | Calyptrocarya glomerulata (Brongn.) Urb. | species |
| Vegetation | Camonea umbellata (L.) A.R. Simões & Staples | species |
| Vegetation | Campomanesia aromatica (Aubl.) Griseb. | species |
| Vegetation | Campomanesia lineatifolia Ruiz & Pav. | species |
| Vegetation | Campomanesia xanthocarpa Mart. ex O. Berg | species |
| Vegetation | Canavalia brasiliensis Mart. ex Benth. | species |
| Vegetation | Canavalia rosea (Sw.) DC. | species |
| Vegetation | Canna × generalis L.H. Bailey | species |
| Vegetation | Canna indica L. | species |
| Vegetation | Cantinoa mutabilis (Rich.) Harley & J.F.B.Pastore | species |
| Vegetation | Caperonia palustris (L.) A.St.-Hil. | species |
| Vegetation | Capraria biflora L. | species |
| Vegetation | Caraipa densifolia Mart. | species |
| Vegetation | Carapa guianensis Aubl. | species |
| Vegetation | Carica papaya L. | species |
| Vegetation | Cariniana estrellensis (Raddi) Kuntze | species |
| Vegetation | Cariniana legalis (Mart.) Kuntze | species |
| Vegetation | Caryocar brasiliense Cambess. | species |
| Vegetation | Caryocar villosum (Aubl.) Pers. | species |
| Vegetation | Casearia arborea (Rich.) Urb. | species |
| Vegetation | Casearia decandra Jacq. | species |
| Vegetation | Casearia grandiflora Cambess. | species |
| Vegetation | Casearia javitensis Kunth | species |
| Vegetation | Casearia mariquitensis Kunth | species |
| Vegetation | Casearia negrensis Eichler | species |
| Vegetation | Casearia pitumba Sleumer | species |
| Vegetation | Casearia sylvestris Sw. | species |
| Vegetation | Cassia fastuosa Willd. ex Benth. | species |
| Vegetation | Cassia fistula L. | species |
| Vegetation | Cassia leiandra Benth. | species |
| Vegetation | Cassipourea guianensis Aubl. | species |
| Vegetation | Cassytha filiformis L. | species |
| Vegetation | Catharanthus roseus (L.) Don | species |
| Vegetation | Cecropia angustifolia Trécul | species |
| Vegetation | Cecropia concolor Willd. | species |
| Vegetation | Cecropia distachya Huber | species |
| Vegetation | Cecropia hololeuca Miq. | species |
| Vegetation | Cecropia membranacea Trécul | species |
| Vegetation | Cecropia pachystachya Trécul | species |
| Vegetation | Cecropia palmata Willd. | species |
| Vegetation | Cecropia peltata L. | species |
| Vegetation | Cecropia polystachya Trécul | species |
| Vegetation | Cedrela odorata L. | species |
| Vegetation | Ceiba pentandra (L.) Gaertn. | species |
| Vegetation | Ceiba speciosa (A.St.-Hil.) Ravenna | species |
| Vegetation | Celosia argentea L. | species |
| Vegetation | Celosia grandifolia Moq. | species |
| Vegetation | Celtis iguanaea (Jacq.) Sarg. | species |
| Vegetation | Cenchrus americanus (L.) Morrone | species |
| Vegetation | Cenchrus echinatus L. | species |
| Vegetation | Cenchrus polystachios (L.) Morrone | species |
| Vegetation | Cenostigma bracteosum (Tul.) Gagnon & G.P.Lewis | species |
| Vegetation | Cenostigma macrophyllum Tul. | species |
| Vegetation | Cenostigma nordestinum Gagnon & G.P.Lewis | species |
| Vegetation | Cenostigma pyramidale (Tul.) Gagnon & G.P.Lewis | species |
| Vegetation | Cenostigma tocantinum Ducke | species |
| Vegetation | Centratherum punctatum Cass. | species |
| Vegetation | Centrolobium tomentosum Guillem. ex Benth. | species |
| Vegetation | Centrosema arenarium Benth. | species |
| Vegetation | Centrosema brasilianum (L.) Benth. | species |
| Vegetation | Centrosema plumieri (Turpin ex Pers.) Benth. | species |
| Vegetation | Centrosema pubescens Benth. | species |
| Vegetation | Centrosema rotundifolium Mart. ex Benth. | species |
| Vegetation | Ceratopteris thalictroides (L.) Brongn. | species |
| Vegetation | Ceratosanthes palmata (L.) Urb. | species |
| Vegetation | Cereus jamacaru DC. | species |
| Vegetation | Cereus mirabella N.P.Taylor | species |
| Vegetation | Cestrum latifolium Lam. | species |
| Vegetation | Chamaecrista apoucouita (Aubl.) H.S.Irwin & Barneby | species |
| Vegetation | Chamaecrista calycioides (DC. ex Collad.) Greene | species |
| Vegetation | Chamaecrista desvauxii (Collad.) Killip | species |
| Vegetation | Chamaecrista diphylla (L.) Greene | species |
| Vegetation | Chamaecrista eitenorum (H.S.Irwin & Barneby) H.S.Irwin & Barneby | species |
| Vegetation | Chamaecrista flexuosa (L.) Greene | species |
| Vegetation | Chamaecrista glandulosa (L.) Greene | species |
| Vegetation | Chamaecrista hispidula (Vahl) H.S.Irwin & Barneby | species |
| Vegetation | Chamaecrista nictitans (L.) Moench | species |
| Vegetation | Chamaecrista ramosa (Vogel) H.S.Irwin & Barneby | species |
| Vegetation | Chamaecrista rotundifolia (Pers.) Greene | species |
| Vegetation | Chamaecrista viscosa (Kunth) H.S.Irwin & Barneby | species |
| Vegetation | Chamissoa altissima (Jacq.) Kunth | species |
| Vegetation | Chaptalia nutans (L.) Pol. | species |
| Vegetation | Cheiloclinium cognatum (Miers) A.C.Sm. | species |
| Vegetation | Chelonanthus alatus (Aubl.) Pulle | species |
| Vegetation | Chevreulia sarmentosa (Pers.) Blake | species |
| Vegetation | Chimarrhis turbinata DC. | species |
| Vegetation | Chiococca alba (L.) Hitchc. | species |
| Vegetation | Chiococca nitida Benth. | species |
| Vegetation | Chloris barbata Sw. | species |
| Vegetation | Chloroleucon acacioides (Ducke) Barneby & J.W.Grimes | species |
| Vegetation | Chloroleucon foliolosum (Benth.) G.P.Lewis | species |
| Vegetation | Chloroleucon tortum (Mart.) Pittier ex Barneby & J.W. Grimes | species |
| Vegetation | Chomelia obtusa Cham. & Schltdl. | species |
| Vegetation | Chomelia tenuiflora Benth. | species |
| Vegetation | Chromolaena laevigata (Lam.) R.M.King & H.Rob. | species |
| Vegetation | Chromolaena maximiliani (Schrad. ex DC.) R.M.King & H.Rob. | species |
| Vegetation | Chromolaena odorata (L.) R.M.King & H.Rob. | species |
| Vegetation | Chrysobalanus icaco L. | species |
| Vegetation | Chrysolaena cognata (Less.) Dematt. | species |
| Vegetation | Chrysophyllum amazonicum T.D.Penn. | species |
| Vegetation | Chrysophyllum pomiferum (Eyma) T.D.Penn. | species |
| Vegetation | Cichorium intybus L. | species |
| Vegetation | Cissus erosa Rich. | species |
| Vegetation | Cissus verticillata (L.) Nicolson & C.E.Jarvis | species |
| Vegetation | Citrus × limon (L.) Osbeck | species |
| Vegetation | Clidemia hirta (L.) D.Don | species |
| Vegetation | Clitoria amazonum Mart. ex Benth. | species |
| Vegetation | Clitoria fairchildiana R.A.Howard | species |
| Vegetation | Clitoria falcata Lam. | species |
| Vegetation | Clitoria guianensis (Aubl.) Benth. | species |
| Vegetation | Clitoria laurifolia Poir. | species |
| Vegetation | Clitoria ternatea L. | species |
| Vegetation | Cnidoscolus adenochlamys Fern.Casas | species |
| Vegetation | Cnidoscolus obtusifolius Pohl ex Baill. | species |
| Vegetation | Cnidoscolus urens (L.) Arthur | species |
| Vegetation | Coccoloba densifrons Mart. ex Meisn. | species |
| Vegetation | Coccoloba latifolia Lam. | species |
| Vegetation | Coccoloba mollis Casar. | species |
| Vegetation | Coccoloba ovata Benth. | species |
| Vegetation | Coccoloba ramosissima Wedd. | species |
| Vegetation | Cochlospermum orinocense (Kunth) Steud. | species |
| Vegetation | Cochlospermum regium (Mart. ex Schrank) Pilg. | species |
| Vegetation | Cochlospermum vitifolium (Willd.) Spreng. | species |
| Vegetation | Cocos nucifera L. | species |
| Vegetation | Coix lacryma-jobi L. | species |
| Vegetation | Combretum lanceolatum Pohl ex Eichler | species |
| Vegetation | Commelina benghalensis L. | species |
| Vegetation | Commelina erecta L. | species |
| Vegetation | Commelina virginica L. | species |
| Vegetation | Connarus favosus Planch. | species |
| Vegetation | Connarus perrottetii (DC.) Planch. | species |
| Vegetation | Connarus regnellii G.Schellenb. | species |
| Vegetation | Connarus suberosus Planch. | species |
| Vegetation | Conocarpus erectus L. | species |
| Vegetation | Conyza bonariensis (L.) Cronquist | species |
| Vegetation | Copaifera duckei Dwyer | species |
| Vegetation | Copaifera guyanensis Desf. | species |
| Vegetation | Copaifera langsdorffii Desf. | species |
| Vegetation | Copaifera martii Hayne | species |
| Vegetation | Copaifera multijuga Hayne | species |
| Vegetation | Copaifera oblongifolia Mart. ex Hayne | species |
| Vegetation | Copaifera officinalis (Jacq.) L. | species |
| Vegetation | Copernicia prunifera (Mill.) H.E.Moore | species |
| Vegetation | Corchorus argutus Kunth | species |
| Vegetation | Cordia bicolor A.DC. | species |
| Vegetation | Cordia exaltata Lam. | species |
| Vegetation | Cordia fallax I.M.Johnst. | species |
| Vegetation | Cordia goeldiana Huber | species |
| Vegetation | Cordia nodosa Lam. | species |
| Vegetation | Cordia scabrifolia A.DC. | species |
| Vegetation | Cordia sellowiana Cham. | species |
| Vegetation | Cordia tetrandra Aubl. | species |
| Vegetation | Cordia toqueve Aubl. | species |
| Vegetation | Cordia trichotoma (Vell.) Arráb. ex Steud. | species |
| Vegetation | Cordiera myrciifolia (K.Schum.) C.H.Perss. & Delprete | species |
| Vegetation | Costus arabicus L. | species |
| Vegetation | Costus spicatus (Jacq.) Sw. | species |
| Vegetation | Costus spiralis (Jacq.) Roscoe | species |
| Vegetation | Couepia bracteosa Benth. | species |
| Vegetation | Couepia guianensis Aubl. | species |
| Vegetation | Couratari guianensis Aubl. | species |
| Vegetation | Couratari macrosperma A.C.Sm. | species |
| Vegetation | Couratari multiflora (Sm.) Eyma | species |
| Vegetation | Couroupita guianensis Aubl. | species |
| Vegetation | Coutoubea spicata Aubl. | species |
| Vegetation | Crateva tapia L. | species |
| Vegetation | Cratylia argentea (Desv.) Kuntze | species |
| Vegetation | Crescentia cujete L. | species |
| Vegetation | Crinum americanum L. | species |
| Vegetation | Crotalaria breviflora DC. | species |
| Vegetation | Crotalaria incana L. | species |
| Vegetation | Crotalaria lanceolata E.Mey. | species |
| Vegetation | Crotalaria micans Link | species |
| Vegetation | Crotalaria pallida Aiton | species |
| Vegetation | Crotalaria retusa L. | species |
| Vegetation | Crotalaria spectabilis Roth | species |
| Vegetation | Croton adamantinus Müll.Arg. | species |
| Vegetation | Croton agoensis Baill. | species |
| Vegetation | Croton argenteus L. | species |
| Vegetation | Croton cajucara Benth. | species |
| Vegetation | Croton campestris A.St.-Hil. | species |
| Vegetation | Croton cuneatus Klotzsch | species |
| Vegetation | Croton draconoides Müll.Arg. | species |
| Vegetation | Croton echioides Baill. | species |
| Vegetation | Croton floribundus Spreng. | species |
| Vegetation | Croton glandulosus L. | species |
| Vegetation | Croton hirtus L'Hér. | species |
| Vegetation | Croton lundianus (Didr.) Müll.Arg. | species |
| Vegetation | Croton matourensis Aubl. | species |
| Vegetation | Croton nepetifolius Baill. | species |
| Vegetation | Croton paraensis Müll.Arg. | species |
| Vegetation | Croton sipaliwinensis Lanj. | species |
| Vegetation | Croton trinitatis Millsp. | species |
| Vegetation | Croton triqueter Lam. | species |
| Vegetation | Croton urucurana Baill. | species |
| Vegetation | Cryptostegia grandiflora R.Br. | species |
| Vegetation | Ctenodon brasilianus (Poir.) D.B.O.S.Cardoso, P.L.R.Moraes & H.C.Lima | species |
| Vegetation | Ctenodon brevipes (Benth.) D.B.O.S.Cardoso, P.L.R.Moraes & H.C.Lima | species |
| Vegetation | Ctenodon histrix (Poir.) D.B.O.S.Cardoso, P.L.R.Moraes & H.C.Lima | species |
| Vegetation | Ctenodon paniculatus (Willd. ex Vogel) D.B.O.S.Cardoso, P.L.R.Moraes & H.C.Lima | species |
| Vegetation | Cucumis anguria L. | species |
| Vegetation | Cucurbita pepo L. | species |
| Vegetation | Cupania diphylla Vahl | species |
| Vegetation | Cupania olivacea Gleason & A.C.Sm. | species |
| Vegetation | Cupania scrobiculata Rich. | species |
| Vegetation | Cupania vernalis Cambess. | species |
| Vegetation | Cuphea flava Spreng. | species |
| Vegetation | Cuphea racemosa (L.f.) Spreng. | species |
| Vegetation | Curatella americana L. | species |
| Vegetation | Cyanthillium cinereum (L.) H.Rob. | species |
| Vegetation | Cybianthus spicatus (Kunth) G.Agostini | species |
| Vegetation | Cycas revoluta Thunb. | species |
| Vegetation | Cyclosorus interruptus (Willd.) H. Ito | species |
| Vegetation | Cymbopetalum euneurum N.A.Murray | species |
| Vegetation | Cynodon dactylon (L.) Pers. | species |
| Vegetation | Cynophalla flexuosa (L.) J.Presl | species |
| Vegetation | Cyperus aggregatus (Willd.) Endl. | species |
| Vegetation | Cyperus articulatus L. | species |
| Vegetation | Cyperus blepharoleptos Steud. | species |
| Vegetation | Cyperus brevifolius (Rottb.) Endl. ex Hassk. | species |
| Vegetation | Cyperus compressus L. | species |
| Vegetation | Cyperus crassipes Vahl | species |
| Vegetation | Cyperus difformis L. | species |
| Vegetation | Cyperus digitatus Roxb. | species |
| Vegetation | Cyperus distans L. | species |
| Vegetation | Cyperus esculentus L. | species |
| Vegetation | Cyperus flavescens L. | species |
| Vegetation | Cyperus giganteus Vahl | species |
| Vegetation | Cyperus haspan L. | species |
| Vegetation | Cyperus hermaphroditus (Jacq.) Standl. | species |
| Vegetation | Cyperus hortensis (Salzm. ex Steud.) Dorr | species |
| Vegetation | Cyperus iria L. | species |
| Vegetation | Cyperus lanceolatus Poir. | species |
| Vegetation | Cyperus laxus Lam. | species |
| Vegetation | Cyperus ligularis L. | species |
| Vegetation | Cyperus luzulae (L.) Retz. | species |
| Vegetation | Cyperus macrostachyos Lam. | species |
| Vegetation | Cyperus mundtii (Nees) Kunth | species |
| Vegetation | Cyperus obtusatus (J. Presl & C. Presl) Mattf. & Kük. | species |
| Vegetation | Cyperus odoratus L. | species |
| Vegetation | Cyperus polystachyos Rottb. | species |
| Vegetation | Cyperus reflexus Vahl | species |
| Vegetation | Cyperus rotundus L. | species |
| Vegetation | Cyperus sesquiflorus (Torr.) Mattf. & Kük. | species |
| Vegetation | Cyperus sphacelatus Rottb. | species |
| Vegetation | Cyperus subsquarrosus (Muhl.) Bauters | species |
| Vegetation | Cyperus surinamensis Rottb. | species |
| Vegetation | Cyperus virens Michx. | species |
| Vegetation | Cyrtocymura scorpioides (Lam.) H.Rob. | species |
| Vegetation | Cyrtopodium holstii L.C.Menezes | species |
| Vegetation | Dactyloctenium aegyptium (L.) Willd. | species |
| Vegetation | Dalbergia ecastaphyllum (L.) Taub. | species |
| Vegetation | Dalbergia monetaria L.f. | species |
| Vegetation | Dalbergia nigra (Vell.) Allemão ex Benth. | species |
| Vegetation | Dalbergia riparia (Mart. ex Benth.) Benth. | species |
| Vegetation | Dalbergia spruceana Benth. | species |
| Vegetation | Dalechampia affinis Müll. Arg. | species |
| Vegetation | Dalechampia caperonioides Baill. | species |
| Vegetation | Dalechampia ficifolia Lam. | species |
| Vegetation | Dalechampia pernambucensis Baill. | species |
| Vegetation | Dalechampia scandens L. | species |
| Vegetation | Dalechampia tiliifolia Lam. | species |
| Vegetation | Davilla elliptica A.St.-Hil. | species |
| Vegetation | Davilla flexuosa A.St.-Hil. | species |
| Vegetation | Davilla latifolia Casar. | species |
| Vegetation | Davilla nitida (Vahl) Kubitzki | species |
| Vegetation | Davilla rugosa Poir. | species |
| Vegetation | Delonix regia (Bojer ex Hook.) Raf. | species |
| Vegetation | Desmodium adscendens (Sw.) DC. | species |
| Vegetation | Desmodium affine Schltdl. | species |
| Vegetation | Desmodium axillare (Sw.) DC. | species |
| Vegetation | Desmodium barbatum (L.) Benth. | species |
| Vegetation | Desmodium distortum (Aubl.) J.F.Macbr. | species |
| Vegetation | Desmodium incanum (Sw.) DC. | species |
| Vegetation | Desmodium subsecundum Vogel | species |
| Vegetation | Desmodium triflorum (L.) DC. | species |
| Vegetation | Desmoncus horridus Splitg. ex Mart. | species |
| Vegetation | Desmoncus orthacanthos Mart. | species |
| Vegetation | Desmoncus polyacanthos Mart. | species |
| Vegetation | Dialium guianense (Aubl.) Sandwith | species |
| Vegetation | Dichapetalum latifolium Baill. | species |
| Vegetation | Dichorisandra hexandra (Aubl.) C.B.Clarke | species |
| Vegetation | Dicliptera ciliaris Juss. | species |
| Vegetation | Dicorynia guianensis Amshoff | species |
| Vegetation | Didymopanax morototoni (Aubl.) Decne. & Planch. | species |
| Vegetation | Dieffenbachia seguine (Jacq.) Schott | species |
| Vegetation | Digitaria bicornis (Lam.) Roem. & Schult. | species |
| Vegetation | Digitaria ciliaris (Retz.) Koeler | species |
| Vegetation | Digitaria horizontalis Willd. | species |
| Vegetation | Digitaria sanguinalis (L.) Scop. | species |
| Vegetation | Dimorphandra gardneriana Tul. | species |
| Vegetation | Dimorphandra mollis Benth. | species |
| Vegetation | Dioclea burkartii R.H.Maxwell | species |
| Vegetation | Dioclea guianensis Benth. | species |
| Vegetation | Dioclea virgata (Rich.) Amshoff | species |
| Vegetation | Dioscorea piperifolia Humb. & Bonpl. ex Willd. | species |
| Vegetation | Diplopterys pubipetala (A.Juss.) W.R.Anderson & C.C.Davis | species |
| Vegetation | Diplotropis purpurea (Rich.) Amshoff | species |
| Vegetation | Dipteryx alata Vogel | species |
| Vegetation | Dipteryx lacunifera Ducke | species |
| Vegetation | Dipteryx magnifica (Ducke) Ducke | species |
| Vegetation | Dipteryx odorata (Aubl.) Forsyth f. | species |
| Vegetation | Dipteryx polyphylla Huber | species |
| Vegetation | Distimake aegyptius (L.) A.R. Simões & Staples | species |
| Vegetation | Distimake cissoides (Lam.) A.R. Simões & Staples | species |
| Vegetation | Distimake macrocalyx (Ruiz & Pav.) A.R. Simões & Staples | species |
| Vegetation | Distimake tuberosus (L.) A.R. Simões & Staples | species |
| Vegetation | Ditassa blanchetii Decne. | species |
| Vegetation | Ditassa hispida (Vell.) Fontella | species |
| Vegetation | Dodonaea viscosa Jacq. | species |
| Vegetation | Dolichandra unguis-cati (L.) L.G.Lohmann | species |
| Vegetation | Doliocarpus dentatus (Aubl.) Standl. | species |
| Vegetation | Doliocarpus magnificus Sleumer | species |
| Vegetation | Dracontium asperum K.Koch | species |
| Vegetation | Duckeodendron cestroides Kuhlm. | species |
| Vegetation | Duguetia argentea (R.E.Fr.) R.E.Fr. | species |
| Vegetation | Duguetia cadaverica Huber | species |
| Vegetation | Duguetia echinophora R.E.Fr. | species |
| Vegetation | Duguetia flagellaris Huber | species |
| Vegetation | Duguetia furfuracea (A.St.-Hil.) Saff. | species |
| Vegetation | Duguetia marcgraviana Mart. | species |
| Vegetation | Duguetia megalocarpa Maas | species |
| Vegetation | Duguetia surinamensis R.E.Fr. | species |
| Vegetation | Dulacia candida (Poepp.) Kuntze | species |
| Vegetation | Dulacia guianensis (Engl.) Kuntze | species |
| Vegetation | Duranta erecta L. | species |
| Vegetation | Duroia macrophylla Huber | species |
| Vegetation | Dypsis lutescens (H.Wendl.) Beentje & J.Dransf. | species |
| Vegetation | Dysphania ambrosioides (L.) Mosyakin & Clemants | species |
| Vegetation | Echinochloa colona (L.) Link | species |
| Vegetation | Echinochloa polystachya (Kunth) Hitchc. | species |
| Vegetation | Echinodorus grandiflorus (Cham. & Schltr.) Micheli | species |
| Vegetation | Echinodorus lanceolatus Rataj | species |
| Vegetation | Echinodorus paniculatus Micheli | species |
| Vegetation | Eclipta prostrata (L.) L. | species |
| Vegetation | Eichhornia crassipes (Mart.) Solms | species |
| Vegetation | Eleocharis acutangula (Roxb.) Schult. | species |
| Vegetation | Eleocharis elegans (Kunth) Roem. & Schult. | species |
| Vegetation | Eleocharis filiculmis Kunth | species |
| Vegetation | Eleocharis geniculata (L.) Roem. & Schult. | species |
| Vegetation | Eleocharis interstincta (Vahl) Roem. & Schult. | species |
| Vegetation | Eleocharis mutata (L.) Roem. & Schult. | species |
| Vegetation | Eleocharis nudipes (Kunth) Palla | species |
| Vegetation | Eleocharis obtusetrigona (Lindl. & Nees) Steud. | species |
| Vegetation | Eleocharis sellowiana Kunth | species |
| Vegetation | Elephantopus mollis Kunth | species |
| Vegetation | Eleusine indica (L.) Gaertn. | species |
| Vegetation | Eleutheranthera ruderalis (Sw.) Sch. Bip. | species |
| Vegetation | Emilia fosbergii Nicolson | species |
| Vegetation | Emilia sonchifolia (L.) DC. | species |
| Vegetation | Emmotum nitens (Benth.) Miers | species |
| Vegetation | Entada polyphylla Benth. | species |
| Vegetation | Entada polystachya (L.) DC. | species |
| Vegetation | Enterolobium schomburgkii (Benth.) Benth. | species |
| Vegetation | Enterolobium timbouva Mart. | species |
| Vegetation | Enterolobium maximum Ducke | species |
| Vegetation | Epaltes brasiliensis DC. | species |
| Vegetation | Eperua glabriflora (Ducke) R.S.Cowan | species |
| Vegetation | Ephedranthus parviflorus S.Moore | species |
| Vegetation | Ephedranthus pisocarpus R.E.Fr. | species |
| Vegetation | Epipremnum pinnatum (L.) Engl. | species |
| Vegetation | Eragrostis ciliaris (L.) R.Br. | species |
| Vegetation | Eragrostis maypurensis (Kunth) Steud. | species |
| Vegetation | Eragrostis pilosa (L.) P.Beauv. | species |
| Vegetation | Eragrostis rufescens Schrad. ex Schult. | species |
| Vegetation | Eragrostis tenella (L.) P.Beauv. ex Roem. & Schult. | species |
| Vegetation | Erechtites hieracifolius (L.) Raf. ex DC. | species |
| Vegetation | Eriotheca globosa (Aubl.) A.Robyns | species |
| Vegetation | Erythrina amazonica Krukoff | species |
| Vegetation | Erythrina crista-galli L. | species |
| Vegetation | Erythrina fusca Lour. | species |
| Vegetation | Erythrina mulungu Mart. ex Benth. | species |
| Vegetation | Erythrina speciosa Andrews | species |
| Vegetation | Erythrina velutina Willd. | species |
| Vegetation | Erythrina verna Vell. | species |
| Vegetation | Erythroxylum barbatum O.E.Schulz | species |
| Vegetation | Erythroxylum bezerrae Plowman | species |
| Vegetation | Erythroxylum citrifolium A.St.-Hil. | species |
| Vegetation | Erythroxylum deciduum A.St.-Hil. | species |
| Vegetation | Erythroxylum ligustrinum DC. | species |
| Vegetation | Erythroxylum passerinum Mart. | species |
| Vegetation | Erythroxylum pungens O.E.Schulz | species |
| Vegetation | Eschweilera atropetiolata S.A.Mori | species |
| Vegetation | Eschweilera coriacea (DC.) S.A.Mori | species |
| Vegetation | Eschweilera grandiflora (Aubl.) Sandwith | species |
| Vegetation | Eschweilera ovata (Cambess.) Mart. ex Miers | species |
| Vegetation | Eschweilera pedicellata (Rich.) S.A.Mori | species |
| Vegetation | Eugenia biflora (L.) DC. | species |
| Vegetation | Eugenia cachoeirensis O.Berg | species |
| Vegetation | Eugenia cupulata Amshoff | species |
| Vegetation | Eugenia densiracemosa Mazine & Faria | species |
| Vegetation | Eugenia dysenterica (Mart.) DC. | species |
| Vegetation | Eugenia egensis DC. | species |
| Vegetation | Eugenia flavescens DC. | species |
| Vegetation | Eugenia florida DC. | species |
| Vegetation | Eugenia lambertiana DC. | species |
| Vegetation | Eugenia patrisii Vahl | species |
| Vegetation | Eugenia polystachya Rich. | species |
| Vegetation | Eugenia protenta McVaugh | species |
| Vegetation | Eugenia punicifolia (Kunth) DC. | species |
| Vegetation | Eugenia stictopetala Mart. ex DC. | species |
| Vegetation | Eugenia citrifolia Poir. | species |
| Vegetation | Eugenia longiracemosa Kiaersk. | species |
| Vegetation | Eugenia omissa McVaugh | species |
| Vegetation | Eugenia stictopetala DC. | species |
| Vegetation | Euphorbia adenoptera Bertol. | species |
| Vegetation | Euphorbia heterophylla L. | species |
| Vegetation | Euphorbia hirta L. | species |
| Vegetation | Euphorbia hypericifolia L. | species |
| Vegetation | Euphorbia hyssopifolia L. | species |
| Vegetation | Euphorbia milii Des Moul. | species |
| Vegetation | Euphorbia prostrata Aiton | species |
| Vegetation | Euphorbia tirucalli L. | species |
| Vegetation | Euphorbia tithymaloides L. | species |
| Vegetation | Euploca filiformis (Lehm.) J.I.M.Melo & Semir | species |
| Vegetation | Euploca polyphylla (Lehm.) J.I.M.Melo & Semir | species |
| Vegetation | Euterpe edulis Mart. | species |
| Vegetation | Euterpe oleracea Mart. | species |
| Vegetation | Euterpe precatoria Mart. | species |
| Vegetation | Evolvulus glomeratus Nees & Mart. | species |
| Vegetation | Falcataria moluccana  (Miq.) Barneby & J.W.Grimes | species |
| Vegetation | Faramea marginata Cham. | species |
| Vegetation | Faramea nitida Benth. | species |
| Vegetation | Ficus aripuanensis C.C.Berg & Kooy | species |
| Vegetation | Ficus benjamina L. | species |
| Vegetation | Ficus catappifolia Kunth & C.D.Bouché | species |
| Vegetation | Ficus christianii Carauta | species |
| Vegetation | Ficus citrifolia Mill. | species |
| Vegetation | Ficus dendrocida Kunth | species |
| Vegetation | Ficus nymphaeifolia Mill. | species |
| Vegetation | Ficus obtusifolia Kunth | species |
| Vegetation | Ficus subapiculata (Miq.) Miq. | species |
| Vegetation | Ficus nymphaeifolia Mill. | species |
| Vegetation | Ficus tapajozensis Standl. | species |
| Vegetation | Fimbristylis autumnalis (L.) Roem. & Schult. | species |
| Vegetation | Fimbristylis cymosa R.Br. | species |
| Vegetation | Fimbristylis dichotoma (L.) Vahl | species |
| Vegetation | Fimbristylis littoralis Gaudich. | species |
| Vegetation | Fimbristylis spadicea (L.) Vahl | species |
| Vegetation | Fridericia caudigera (S.Moore) L.G.Lohmann | species |
| Vegetation | Fridericia conjugata (Vell.) L.G.Lohmann | species |
| Vegetation | Fridericia dispar (Bureau ex K.Schum.) L.G.Lohmann | species |
| Vegetation | Fridericia japurensis (DC.) L.G.Lohmann | species |
| Vegetation | Fridericia nigrescens (Sandwith) L.G.Lohmann | species |
| Vegetation | Fridericia platyphylla (Cham.) L.G.Lohmann | species |
| Vegetation | Fridericia triplinervia (Mart. ex DC.) L.G.Lohmann | species |
| Vegetation | Fuirena robusta Kunth | species |
| Vegetation | Fuirena umbellata Rottb. | species |
| Vegetation | Funastrum clausum (Jacq.) Schltr. | species |
| Vegetation | Fusaea longifolia (Aubl.) Saff. | species |
| Vegetation | Galactia jussiaeana Kunth | species |
| Vegetation | Galactia striata (Jacq.) Urb. | species |
| Vegetation | Galinsoga quadriradiata Ruiz & Pav. | species |
| Vegetation | Galipea jasminiflora (A.St.-Hil.) Engl. | species |
| Vegetation | Garcinia gardneriana (Planch. & Triana) Zappi | species |
| Vegetation | Garcinia macrophylla Mart. | species |
| Vegetation | Garcinia madruno (Kunth) Hammel | species |
| Vegetation | Gaya pilosa K.Schum. | species |
| Vegetation | Genipa americana L. | species |
| Vegetation | Gliricidia sepium (Jacq.) Kunth ex Walp. | species |
| Vegetation | Gmelina arborea Roxb. ex Sm. | species |
| Vegetation | Goeppertia arrabidae (Körn.) Borchs. & S. Suárez | species |
| Vegetation | Goeppertia bachemiana (É.Morren) Borchs. & S.Suárez | species |
| Vegetation | Goeppertia villosa (Lodd. ex Sweet) Borchs. & S. Suárez | species |
| Vegetation | Gomphrena celosioides Mart. | species |
| Vegetation | Gomphrena globosa L. | species |
| Vegetation | Gomphrena leucocephala Mart. | species |
| Vegetation | Gossypium barbadense L. | species |
| Vegetation | Gossypium hirsutum L. | species |
| Vegetation | Gossypium mustelinum Miers ex G. Watt | species |
| Vegetation | Gouania cornifolia Reissek | species |
| Vegetation | Guapira hirsuta (Choisy) Lundell | species |
| Vegetation | Guapira opposita (Vell.) Reitz | species |
| Vegetation | Guapira pernambucensis (Casar.) Lundell | species |
| Vegetation | Guarea guidonia (L.) Sleumer | species |
| Vegetation | Guarea kunthiana  A.Juss. | species |
| Vegetation | Guatteria citriodora Ducke | species |
| Vegetation | Guatteria foliosa Benth. | species |
| Vegetation | Guatteria punctata (Aubl.) R.A.Howard | species |
| Vegetation | Guatteria schomburgkiana Mart. | species |
| Vegetation | Guazuma ulmifolia Lam. | species |
| Vegetation | Guettarda angelica Mart. ex Müll.Arg. | species |
| Vegetation | Guettarda argentea Lam. | species |
| Vegetation | Guettarda platypoda DC. | species |
| Vegetation | Guettarda spruceana Müll.Arg. | species |
| Vegetation | Guettarda viburnoides Cham. & Schltdl. | species |
| Vegetation | Guibourtia hymenaeifolia (Moric.) J.Léonard | species |
| Vegetation | Guilandina bonduc L. | species |
| Vegetation | Gustavia augusta L. | species |
| Vegetation | Gymnanthemum amygdalinum (Delile) Sch.Bip. ex Walp. | species |
| Vegetation | Handroanthus arianeae (A.H.Gentry) S.Grose | species |
| Vegetation | Handroanthus chrysotrichus (Mart. ex DC.) Mattos | species |
| Vegetation | Handroanthus heptaphyllus (Mart.) Mattos | species |
| Vegetation | Handroanthus impetiginosus (Mart. ex DC.) Mattos | species |
| Vegetation | Handroanthus ochraceus (Cham.) Mattos | species |
| Vegetation | Handroanthus serratifolius (Vahl) S.Grose | species |
| Vegetation | Heisteria acuminata (Humb. & Bonpl.) Engl. | species |
| Vegetation | Heisteria laxiflora Engl. | species |
| Vegetation | Heisteria ovata Benth. | species |
| Vegetation | Heisteria silvianii Schwacke | species |
| Vegetation | Helanthium tenellum (Mart. ex Schult. f.) Britton | species |
| Vegetation | Heliconia acuminata L.C.Rich. | species |
| Vegetation | Heliconia bihai (L.) L. | species |
| Vegetation | Heliconia densiflora Verl. | species |
| Vegetation | Heliconia hirsuta L.f. | species |
| Vegetation | Heliconia psittacorum L.f. | species |
| Vegetation | Heliconia rostrata Ruiz & Pav. | species |
| Vegetation | Helicteres heptandra L.B.Sm. | species |
| Vegetation | Heliotropium funkiae Feuillet | species |
| Vegetation | Heliotropium indicum L. | species |
| Vegetation | Heteropterys eglandulosa A.Juss. | species |
| Vegetation | Hexasepalum teres (Walter) J.H.Kirkbr. | species |
| Vegetation | Hibiscus dimidiatus Schrank | species |
| Vegetation | Hibiscus rosa-sinensis L. | species |
| Vegetation | Hibiscus sabdariffa L. | species |
| Vegetation | Hibiscus schizopetalus (Dyer) Hook. f. | species |
| Vegetation | Hieronyma alchorneoides Allemão | species |
| Vegetation | Himatanthus articulatus (Vahl) Woodson | species |
| Vegetation | Himatanthus drasticus (Mart.) Plumel | species |
| Vegetation | Himatanthus obovatus (Müll. Arg.) Woodson | species |
| Vegetation | Hippeastrum elegans (Spreng.) H.E.Moore | species |
| Vegetation | Hippeastrum puniceum (Lam.) Voss | species |
| Vegetation | Hippeastrum stylosum Herb. | species |
| Vegetation | Hirtella racemosa Lam. | species |
| Vegetation | Homalolepis cedron (Planch.) Devecchi & Pirani | species |
| Vegetation | Homalolepis paraensis (Ducke) Devecchi & Pirani | species |
| Vegetation | Hortia brasiliana Vand. ex DC. | species |
| Vegetation | Hydrocleys modesta Pedersen | species |
| Vegetation | Hydrocotyle bonariensis Lam. | species |
| Vegetation | Hydrolea spinosa L. | species |
| Vegetation | Hylenaea comosa (Sw.) Miers | species |
| Vegetation | Hymenachne amplexicaulis (Rudge) Nees | species |
| Vegetation | Hymenachne pernambucensis (Spreng.) Zuloaga | species |
| Vegetation | Hymenaea courbaril L. | species |
| Vegetation | Hymenaea intermedia Ducke | species |
| Vegetation | Hymenaea maranhensis Lee & Lang. | species |
| Vegetation | Hymenaea oblongifolia Huber | species |
| Vegetation | Hymenaea parvifolia Huber | species |
| Vegetation | Hymenaea stigonocarpa Mart. ex Hayne | species |
| Vegetation | Hymenolobium excelsum Ducke | species |
| Vegetation | Hymenolobium petraeum Ducke | species |
| Vegetation | Hymenopus heteromorphus (Benth.) Sothers & Prance | species |
| Vegetation | Hymenopus reticulatus (Prance) Sothers & Prance | species |
| Vegetation | Hyparrhenia rufa (Nees) Stapf | species |
| Vegetation | Hyptis atrorubens Poit. | species |
| Vegetation | Ibatia ganglinosa (Vell.) Morillo | species |
| Vegetation | Ibatia maritima (Jacq.) Decne. | species |
| Vegetation | Impatiens walleriana Hook.f. | species |
| Vegetation | Imperata brasiliensis Trin. | species |
| Vegetation | Indigofera blanchetiana Benth. | species |
| Vegetation | Indigofera hirsuta L. | species |
| Vegetation | Indigofera microcarpa Desv. | species |
| Vegetation | Indigofera suffruticosa Mill. | species |
| Vegetation | Indigofera truxillensis Kunth | species |
| Vegetation | Inga alba (Sw.) Willd. | species |
| Vegetation | Inga bourgonii (Aubl.) DC. | species |
| Vegetation | Inga capitata Desv. | species |
| Vegetation | Inga cayennensis Sagot ex Benth. | species |
| Vegetation | Inga cinnamomea Spruce ex Benth. | species |
| Vegetation | Inga cylindrica (Vell.) Mart. | species |
| Vegetation | Inga disticha Benth. | species |
| Vegetation | Inga edulis Mart. | species |
| Vegetation | Inga gracilifolia Ducke | species |
| Vegetation | Inga grandiflora Ducke | species |
| Vegetation | Inga heterophylla Willd. | species |
| Vegetation | Inga ingoides (Rich.) Willd. | species |
| Vegetation | Inga laurina (Sw.) Willd. | species |
| Vegetation | Inga macrophylla Humb. & Bonpl. ex Willd. | species |
| Vegetation | Inga marginata Willd. | species |
| Vegetation | Inga nobilis Willd. | species |
| Vegetation | Inga obtusata Spruce ex Benth. | species |
| Vegetation | Inga paraensis Ducke | species |
| Vegetation | Inga pilosula (Rich.) J.F.Macbr. | species |
| Vegetation | Inga punctata Willd. | species |
| Vegetation | Inga sertulifera DC. | species |
| Vegetation | Inga sessilis (Vell.) Mart. | species |
| Vegetation | Inga thibaudiana DC. | species |
| Vegetation | Inga umbellifera (Vahl) DC. | species |
| Vegetation | Inga velutina Willd. | species |
| Vegetation | Inga vera Willd. | species |
| Vegetation | Inga gracilifolia Ducke | species |
| Vegetation | Inga heterophylla Willd. | species |
| Vegetation | Inga macrophylla Humb. & Bonpl. ex Willd. | species |
| Vegetation | Inga thibaudiana DC. | species |
| Vegetation | Ipomoea acanthocarpa (Choisy) Aschers. & Schweinf. | species |
| Vegetation | Ipomoea alba L. | species |
| Vegetation | Ipomoea aristolochiifolia G.Don | species |
| Vegetation | Ipomoea asarifolia (Desr.) Roem. & Schult. | species |
| Vegetation | Ipomoea bahiensis Willd. ex Roem. & Schult. | species |
| Vegetation | Ipomoea batatas (L.) Lam. | species |
| Vegetation | Ipomoea blanchetii Choisy | species |
| Vegetation | Ipomoea cairica (L.) Sweet | species |
| Vegetation | Ipomoea carnea Jacq. | species |
| Vegetation | Ipomoea imperati (Vahl) Griseb. | species |
| Vegetation | Ipomoea incarnata (Vahl) Choisy | species |
| Vegetation | Ipomoea indica (Burm.) Merr. | species |
| Vegetation | Ipomoea indivisa (Vell.) Hallier f. | species |
| Vegetation | Ipomoea mauritiana Jacq. | species |
| Vegetation | Ipomoea nil (L.) Roth | species |
| Vegetation | Ipomoea pes-caprae (L.) R.Br. | species |
| Vegetation | Ipomoea philomega (Vell.) House | species |
| Vegetation | Ipomoea platensis Ker Gawl. | species |
| Vegetation | Ipomoea purpurea (L.) Roth | species |
| Vegetation | Ipomoea quamoclit L. | species |
| Vegetation | Ipomoea setifera Poir. | species |
| Vegetation | Ipomoea squamosa Choisy | species |
| Vegetation | Ipomoea triloba L. | species |
| Vegetation | Ischaemum rugosum Salisb. | species |
| Vegetation | Ischnosiphon arouma (Aubl.) Körn. | species |
| Vegetation | Isertia spiciformis DC. | species |
| Vegetation | Ixora casei Hance | species |
| Vegetation | Ixora chinensis Lam. | species |
| Vegetation | Ixora coccinea L. | species |
| Vegetation | Jacaranda brasiliana (Lam.) Pers. | species |
| Vegetation | Jacaranda copaia (Aubl.) D.Don | species |
| Vegetation | Jacaranda cuspidifolia Mart. | species |
| Vegetation | Jacaranda duckei Vattimo | species |
| Vegetation | Jacaranda heterophylla M.M.Silva | species |
| Vegetation | Jacaratia spinosa (Aubl.) A.DC. | species |
| Vegetation | Jacquemontia guyanensis (Aubl.) Meisn. | species |
| Vegetation | Jatropha curcas L. | species |
| Vegetation | Jatropha gossypiifolia L. | species |
| Vegetation | Juncus microcephalus Kunth | species |
| Vegetation | Justicia brandegeeana Wassh. & L.B.Sm. | species |
| Vegetation | Justicia calycina (Nees) V.A.W.Graham | species |
| Vegetation | Justicia laevilinguis (Nees) Lindau | species |
| Vegetation | Justicia pectoralis Jacq. | species |
| Vegetation | Justicia secunda Vahl | species |
| Vegetation | Lacistema aggregatum (P.J. Bergius) Rusby | species |
| Vegetation | Lacistema pubescens Mart. | species |
| Vegetation | Lactuca sativa L. | species |
| Vegetation | Lacunaria crenata (Tul.) A.C.Sm. | species |
| Vegetation | Laetia suaveolens (Poepp.) Benth. | species |
| Vegetation | Laguncularia racemosa (L.) C.F.Gaertn. | species |
| Vegetation | Lantana camara L. | species |
| Vegetation | Leandra laevigata (Triana) Cogn. | species |
| Vegetation | Lecythis chartacea O.Berg | species |
| Vegetation | Lecythis idatimon Aubl. | species |
| Vegetation | Lecythis lanceolata Poir. | species |
| Vegetation | Lecythis lurida (Miers) S.A.Mori | species |
| Vegetation | Lecythis pisonis Cambess. | species |
| Vegetation | Leea rubra Blume ex Spreng. | species |
| Vegetation | Lemna aequinoctialis Welw. | species |
| Vegetation | Lepidagathis alopecuroidea (Vahl) R.Br. ex Griseb. | species |
| Vegetation | Lepidaploa arenaria (Mart. ex DC.) H.Rob. | species |
| Vegetation | Lepidaploa helophila (Mart. ex DC.) H.Rob. | species |
| Vegetation | Lepidaploa muricata (DC.) H.Rob. | species |
| Vegetation | Lepidaploa remotiflora (Rich.) H.Rob. | species |
| Vegetation | Lepidaploa rufogrisea (A.St.-Hil.) H.Rob. | species |
| Vegetation | Lepidium bonariense L. | species |
| Vegetation | Lepidium virginicum L. | species |
| Vegetation | Leptobalanus apetalus (E.Mey.) Sothers & Prance | species |
| Vegetation | Leptobalanus longistylus (Hook.f.) Sothers & Prance | species |
| Vegetation | Leptobalanus octandrus (Hoffmanns. ex Roem. & Schult.) Sothers & Prance | species |
| Vegetation | Leptolobium dasycarpum Vogel | species |
| Vegetation | Leptospron adenanthum (G.Mey.) A.Delgado | species |
| Vegetation | Leucaena leucocephala (Lam.) de Wit | species |
| Vegetation | Libidibia ferrea (Mart. ex Tul.) L.P.Queiroz | species |
| Vegetation | Licania bracteata Prance | species |
| Vegetation | Licania gracilipes Taub. | species |
| Vegetation | Licania incana Aubl. | species |
| Vegetation | Licania kunthiana Hook.f. | species |
| Vegetation | Licania membranacea Sagot ex Laness. | species |
| Vegetation | Licania micrantha Miq. | species |
| Vegetation | Licania pallida Spruce ex Sagot | species |
| Vegetation | Limnocharis flava (L.) Buchenau | species |
| Vegetation | Lindackeria paraensis Kuhlm. | species |
| Vegetation | Lindackeria pauciflora Benth. | species |
| Vegetation | Lindernia crustacea (L.) F.Muell. | species |
| Vegetation | Lippia alba (Mill.) N.E.Br. ex Britton & P.Wilson | species |
| Vegetation | Lonchocarpus sericeus (Poir.) Kunth ex DC. | species |
| Vegetation | Ludwigia affinis (DC.) H.Hara | species |
| Vegetation | Ludwigia hyssopifolia (G.Don) Exell | species |
| Vegetation | Ludwigia leptocarpa (Nutt.) H. Hara | species |
| Vegetation | Ludwigia octovalvis (Jacq.) P.H.Raven | species |
| Vegetation | Luehea divaricata Mart. | species |
| Vegetation | Luehea grandiflora Mart. | species |
| Vegetation | Luehea speciosa Willd. | species |
| Vegetation | Lygodium venustum Sw. | species |
| Vegetation | Lygodium volubile Sw. | species |
| Vegetation | Mabea angustifolia Spruce ex Benth. | species |
| Vegetation | Mabea fistulifera Mart. | species |
| Vegetation | Mabea paniculata Spruce ex Benth. | species |
| Vegetation | Mabea speciosa Müll. Arg. | species |
| Vegetation | Machaerium aculeatum Raddi | species |
| Vegetation | Machaerium lunatum (L.f.) Ducke | species |
| Vegetation | Machaerium quinata (Aubl.) Sandwith | species |
| Vegetation | Machaerium stipitatum Vogel | species |
| Vegetation | Macrolobium acaciifolium (Benth.) Benth. | species |
| Vegetation | Macrolobium campestre Huber | species |
| Vegetation | Macropsychanthus bicolor (Benth.) L.P.Queiroz & Snak | species |
| Vegetation | Macropsychanthus comosus (G.Mey.) L.P.Queiroz & Snak | species |
| Vegetation | Macropsychanthus grandiflorus (Mart. ex Benth.) L.P.Queiroz & Snak | species |
| Vegetation | Macropsychanthus sclerocarpus (Ducke) L.P.Queiroz | species |
| Vegetation | Macropsychanthus violaceus (Mart. ex Benth.) L.P.Queiroz & Snak | species |
| Vegetation | Macroptilium atropurpureum (DC.) Urb. | species |
| Vegetation | Macroptilium gracile (Poepp. ex Benth.) Urb. | species |
| Vegetation | Macroptilium lathyroides (L.) Urb. | species |
| Vegetation | Macroptilium panduratum (Benth.) Maréchal & Baudet | species |
| Vegetation | Malachra fasciata Jacq. | species |
| Vegetation | Malpighia emarginata DC. | species |
| Vegetation | Malvastrum coromandelianum (L.) Garcke | species |
| Vegetation | Mammea americana L. | species |
| Vegetation | Mandevilla hirsuta (Rich.) K. Schum. | species |
| Vegetation | Mandevilla illustris (Vell.) Woodson | species |
| Vegetation | Mandevilla martii (Müll.Arg.) Pichon | species |
| Vegetation | Mandevilla scaberula N.E.Br. | species |
| Vegetation | Mandevilla scabra (Hoffmanns. ex Roem. & Schult.) K. Schum. | species |
| Vegetation | Mangifera indica L. | species |
| Vegetation | Manihot baccata Allem | species |
| Vegetation | Manihot caerulescens Pohl | species |
| Vegetation | Manihot compositifolia Allem | species |
| Vegetation | Manihot esculenta Crantz | species |
| Vegetation | Manihot leptophylla Pax | species |
| Vegetation | Manihot quinquepartita Huber ex D.J.Rogers & Appan | species |
| Vegetation | Manihot tristis Müll.Arg. | species |
| Vegetation | Manilkara amazonica (Huber) Standl. | species |
| Vegetation | Manilkara bidentata (A.DC.) A.Chev. | species |
| Vegetation | Manilkara cavalcantei Pires & W.A.Rodrigues ex T.D.Penn. | species |
| Vegetation | Manilkara elata (Allemão ex Miq.) Monach. | species |
| Vegetation | Manilkara salzmannii (A. DC.) H. J. Lam | species |
| Vegetation | Manilkara subsericea (Mart.) Dubard | species |
| Vegetation | Manilkara triflora (Allemão) Monach. | species |
| Vegetation | Manilkara zapota (L.) P.Royen | species |
| Vegetation | Mansoa alliacea (Lam.) A.H.Gentry | species |
| Vegetation | Maprounea brasiliensis A.St.-Hil. | species |
| Vegetation | Maprounea guianensis Aubl. | species |
| Vegetation | Maquira guianensis Aubl. | species |
| Vegetation | Margaritaria nobilis L.f. | species |
| Vegetation | Maripa reticulata Ducke | species |
| Vegetation | Marsdenia macrophylla (Humb. & Bonpl. ex Schult.) E.Fourn. | species |
| Vegetation | Marsilea polycarpa Hook. & Grev. | species |
| Vegetation | Marsypianthes chamaedrys (Vahl) Kuntze | species |
| Vegetation | Matayba arborescens (Aubl.) Radlk. | species |
| Vegetation | Matayba discolor (Spreng.) Radlk. | species |
| Vegetation | Matayba elaeagnoides Radlk. | species |
| Vegetation | Matayba guianensis Aubl. | species |
| Vegetation | Mauritia flexuosa L.f. | species |
| Vegetation | Mauritiella armata (Mart.) Burret | species |
| Vegetation | Maytenus guyanensis Klotzsch ex Reissek | species |
| Vegetation | Megathyrsus maximus (Jacq.) B.K.Simon & S.W.L.Jacobs | species |
| Vegetation | Melampodium divaricatum (Rich.) DC. | species |
| Vegetation | Melinis minutiflora P.Beauv. | species |
| Vegetation | Melinis repens (Willd.) Zizka | species |
| Vegetation | Melochia parvifolia Kunth | species |
| Vegetation | Melochia pyramidata L. | species |
| Vegetation | Meniscium hostmannii (Klotzsch) R.S. Fernandes & Salino | species |
| Vegetation | Meniscium serratum Cav. | species |
| Vegetation | Mesosphaerum suaveolens (L.) Kuntze | species |
| Vegetation | Mezilaurus synandra (Mez) Kosterm. | species |
| Vegetation | Miconia alata (Aubl.) DC. | species |
| Vegetation | Miconia ciliata  (Rich.) DC. | species |
| Vegetation | Miconia cuspidata  Naudin | species |
| Vegetation | Miconia gratissima  Benth. ex Triana | species |
| Vegetation | Miconia phanerostila  Pilger | species |
| Vegetation | Miconia prasina (Sw.) DC. | species |
| Vegetation | Miconia serialis DC. | species |
| Vegetation | Micropholis cylindrocarpa (Poepp.) Pierre | species |
| Vegetation | Micropholis gardneriana (A.DC.) Pierre | species |
| Vegetation | Micropholis guyanensis (A.DC.) Pierre | species |
| Vegetation | Micropholis mensalis (Baehni) Aubrév. | species |
| Vegetation | Micropholis williamii Aubrév. & Pellegr. | species |
| Vegetation | Microstachys corniculata (Vahl) Griseb. | species |
| Vegetation | Microtea celosioides Moq. ex Sennikov & Sukhor. | species |
| Vegetation | Mikania cardiophylla B.L.Rob. | species |
| Vegetation | Mikania congesta DC. | species |
| Vegetation | Mikania cordifolia (L. f.) Willd. | species |
| Vegetation | Mikania micrantha Kunth | species |
| Vegetation | Mikania psilostachya DC. | species |
| Vegetation | Mimosa acutistipula (Mart.) Benth. | species |
| Vegetation | Mimosa balansae Micheli | species |
| Vegetation | Mimosa bimucronata (DC.) Kuntze | species |
| Vegetation | Mimosa caesalpiniifolia Benth. | species |
| Vegetation | Mimosa candollei R.Grether | species |
| Vegetation | Mimosa hirsutissima Mart. | species |
| Vegetation | Mimosa invisa Mart. ex Colla | species |
| Vegetation | Mimosa misera Benth. | species |
| Vegetation | Mimosa pigra L. | species |
| Vegetation | Mimosa pudica L. | species |
| Vegetation | Mimosa schomburgkii Benth. | species |
| Vegetation | Mimosa sensitiva L. | species |
| Vegetation | Mimosa velloziana Mart. | species |
| Vegetation | Mimosa caesalpiniifolia Benth. | species |
| Vegetation | Mitracarpus hirtus (L.) DC. | species |
| Vegetation | Mitracarpus strigosus (Thunb.) P.L.R.Moraes, De Smedt & Hjertson | species |
| Vegetation | Mollugo verticillata L. | species |
| Vegetation | Momordica charantia L. | species |
| Vegetation | Monteverdia erythroxyla (Reissek) Biral | species |
| Vegetation | Monteverdia obtusifolia (Mart.) Biral | species |
| Vegetation | Monteverdia robusta (Reissek) Biral | species |
| Vegetation | Montrichardia arborescens (L.) Schott | species |
| Vegetation | Montrichardia linifera (Arruda) Schott | species |
| Vegetation | Moquilea egleri (Prance) Sothers & Prance | species |
| Vegetation | Moquilea tomentosa Benth. | species |
| Vegetation | Mouriri acutiflora Naudin | species |
| Vegetation | Mouriri cearensis Huber | species |
| Vegetation | Mouriri duckeana Morley | species |
| Vegetation | Mouriri grandiflora DC. | species |
| Vegetation | Mouriri guianensis Aubl. | species |
| Vegetation | Mouriri vernicosa Naudin | species |
| Vegetation | Mouriri huberi Cogn. | species |
| Vegetation | Mucuna pruriens (L.) DC. | species |
| Vegetation | Mucuna rostrata Benth. | species |
| Vegetation | Mucuna sloanei Fawc. & Rendle | species |
| Vegetation | Mucuna urens (L.) Medik. | species |
| Vegetation | Muellera campestris (Mart. ex Benth.) M.J. Silva & A.M.G. Azevedo | species |
| Vegetation | Muntingia calabura L. | species |
| Vegetation | Murdannia nudiflora (L.) Brenan | species |
| Vegetation | Murraya paniculata (L.) Jack | species |
| Vegetation | Musa paradisiaca L. | species |
| Vegetation | Myrcia amazonica DC. | species |
| Vegetation | Myrcia bracteata (Rich.) DC. | species |
| Vegetation | Myrcia cuprea (O.Berg) Kiaersk. | species |
| Vegetation | Myrcia guianensis (Aubl.) DC. | species |
| Vegetation | Myrcia inaequiloba (DC.) Lemée | species |
| Vegetation | Myrcia multiflora (Lam.) DC. | species |
| Vegetation | Myrcia neesiana DC. | species |
| Vegetation | Myrcia neospeciosa A.R. Lourenço & E. Lucas | species |
| Vegetation | Myrcia selloi (Spreng.) N.Silveira | species |
| Vegetation | Myrcia splendens (Sw.) DC. | species |
| Vegetation | Myrcia sylvatica (G.Mey.) DC. | species |
| Vegetation | Myrcia bracteata (Rich.) DC. | species |
| Vegetation | Myrcia caudata (McVaugh) E. Lucas & C.E. Wilson | species |
| Vegetation | Myrcia rufipila McVaugh | species |
| Vegetation | Myrcia selloi (Spreng.) N.Silveira | species |
| Vegetation | Myrcia tomentosa (Aubl.) DC. | species |
| Vegetation | Myrcia umbraticola (Kunth) E. Lucas & C.E. Wilson | species |
| Vegetation | Myrciaria cuspidata O.Berg | species |
| Vegetation | Myrciaria tenella (DC.) O.Berg | species |
| Vegetation | Myriopus candidulus (Miers) Feuillet | species |
| Vegetation | Myriopus rubicundus (Salzm. ex DC.) Luebert | species |
| Vegetation | Myriopus villosus (Salzm. ex DC.) J.I.M.Melo | species |
| Vegetation | Myriopus volubilis Small | species |
| Vegetation | Nealchornea yapurensis Huber | species |
| Vegetation | Nectandra reticulata (Ruiz & Pav.) Mez | species |
| Vegetation | Neea floribunda Poepp. & Endl. | species |
| Vegetation | Neea oppositifolia Ruiz & Pav. | species |
| Vegetation | Neoblechnum brasiliense (Desv.) Gasper & V.A.O. Dittrich | species |
| Vegetation | Neomarica northiana (Schneev.) Sprague | species |
| Vegetation | Neoptychocarpus apodanthus (Kuhlm.) Buchheim | species |
| Vegetation | Nephrolepis biserrata (Sw.) Schott | species |
| Vegetation | Nepsera aquatica (Aubl.) Naudin | species |
| Vegetation | Neptunia oleracea Lour. | species |
| Vegetation | Neptunia plena (L.) Benth. | species |
| Vegetation | Nerium oleander L. | species |
| Vegetation | Neustanthus phaseoloides (Roxb.) Benth. var. phaseoloides | species |
| Vegetation | Niedenzuella acutifolia (Cav.) W.R.Anderson | species |
| Vegetation | Noterophila bivalvis (Aubl.) Kriebel & M.J.R.Rocha | species |
| Vegetation | Noterophila crassipes (Naudin) Kriebel & M.J.R.Rocha | species |
| Vegetation | Nymphoides indica (L.) Kuntze | species |
| Vegetation | Ocimum basilicum L. | species |
| Vegetation | Ocotea cernua (Nees) Mez | species |
| Vegetation | Ocotea cujumary Mart. | species |
| Vegetation | Ocotea floribunda (Sw.) Mez | species |
| Vegetation | Ocotea neesiana (Miq.) Kosterm. | species |
| Vegetation | Ocotea nigrescens Vicent. | species |
| Vegetation | Ocotea puberula (Rich.) Nees | species |
| Vegetation | Ocotea pulchella (Nees & Mart.) Mez | species |
| Vegetation | Odontocarya duckei Barneby | species |
| Vegetation | Oeceoclades maculata (Lindl.) Lindl. | species |
| Vegetation | Oenocarpus distichus Mart. | species |
| Vegetation | Oldenlandia corymbosa L. | species |
| Vegetation | Oldenlandia tenuis K.Schum. | species |
| Vegetation | Operculina hamiltonii (G.Don) D.F.Austin & Staples | species |
| Vegetation | Operculina macrocarpa (L.) Urb. | species |
| Vegetation | Oplismenus hirtellus (L.) P.Beauv. | species |
| Vegetation | Ormosia arborea (Vell.) Harms | species |
| Vegetation | Ormosia paraensis Ducke | species |
| Vegetation | Ormosia stipularis Ducke | species |
| Vegetation | Osteophloeum platyspermum (Spruce ex A. DC.) Warb. | species |
| Vegetation | Ouratea castaneifolia (DC.) Engl. | species |
| Vegetation | Ouratea fieldingiana (Gardner) Engl. | species |
| Vegetation | Ouratea hexasperma (A.St.-Hil.) Baill. | species |
| Vegetation | Ouratea spectabilis (Mart.) Engl. | species |
| Vegetation | Ouratea castaneifolia (DC.) Engl. | species |
| Vegetation | Oxandra reticulata Maas | species |
| Vegetation | Oxandra sessiliflora R.E.Fr. | species |
| Vegetation | Oxypetalum banksii R.Br. ex Schult. | species |
| Vegetation | Pachira aquatica Aubl. | species |
| Vegetation | Pachira glabra Pasq. | species |
| Vegetation | Pachira nervosa (Uittien) Fern.Alonso | species |
| Vegetation | Pachira paraensis (Ducke) W.S.Alverson | species |
| Vegetation | Pachystachys lutea Nees | species |
| Vegetation | Pachystroma longifolium (Nees) I.M.Johnst. | species |
| Vegetation | Paepalanthus bifidus (Schrad.) Kunth | species |
| Vegetation | Paepalanthus lamarckii Kunth | species |
| Vegetation | Paepalanthus polytrichoides Kunth | species |
| Vegetation | Paepalanthus subtilis Miq. | species |
| Vegetation | Pagamea guianensis Aubl. | species |
| Vegetation | Palhinhaea cernua (L.) Franco & Vasc. | species |
| Vegetation | Palicourea hoffmannseggiana (Schult.) Borhidi | species |
| Vegetation | Palicourea marcgravii A.St.-Hil. | species |
| Vegetation | Panicum campestre Nees ex Trin. | species |
| Vegetation | Panicum cayennense Lam. | species |
| Vegetation | Panicum hirtum Lam. | species |
| Vegetation | Panicum repens L. | species |
| Vegetation | Panicum trichoides Sw. | species |
| Vegetation | Paratheria prostrata Griseb. | species |
| Vegetation | Pariana campestris Aubl. | species |
| Vegetation | Parinari rodolphii Huber | species |
| Vegetation | Parkia pendula (Willd.) Benth. ex Walp. | species |
| Vegetation | Parkia platycephala Benth. | species |
| Vegetation | Parkia nitida Miq. | species |
| Vegetation | Parkia pendula (Willd.) Benth. ex Walp. | species |
| Vegetation | Parodiophyllochloa ovulifera (Trin.) Zuloaga & Morrone | species |
| Vegetation | Paspalidium geminatum (Forssk.) Stapf | species |
| Vegetation | Paspalum arenarium Schrad. | species |
| Vegetation | Paspalum conjugatum P.J.Bergius | species |
| Vegetation | Paspalum ligulare Nees | species |
| Vegetation | Paspalum maritimum Trin. | species |
| Vegetation | Paspalum melanospermum Desv. ex Poir. | species |
| Vegetation | Paspalum millegrana Schrad. ex Schult. | species |
| Vegetation | Paspalum notatum Flüggé | species |
| Vegetation | Paspalum repens P.J.Bergius | species |
| Vegetation | Paspalum vaginatum Sw. | species |
| Vegetation | Passiflora acuminata DC. | species |
| Vegetation | Passiflora cincinnata Mast. | species |
| Vegetation | Passiflora coccinea Aubl. | species |
| Vegetation | Passiflora edulis Sims | species |
| Vegetation | Passiflora foetida L. | species |
| Vegetation | Passiflora glandulosa Cav. | species |
| Vegetation | Passiflora nitida Kunth | species |
| Vegetation | Passiflora porophylla Vell. | species |
| Vegetation | Passiflora silvestris Vell. | species |
| Vegetation | Passiflora subrotunda Mast. | species |
| Vegetation | Paubrasilia echinata (Lam.) Gagnon, H.C.Lima & G.P.Lewis | species |
| Vegetation | Paullinia pinnata L. | species |
| Vegetation | Pavonia cancellata (L.) Cav. | species |
| Vegetation | Pavonia malacophylla (Link & Otto) Garcke | species |
| Vegetation | Pavonia sidifolia Kunth | species |
| Vegetation | Pectis brevipedunculata (Gardner) Sch.Bip. | species |
| Vegetation | Pectis elongata Kunth | species |
| Vegetation | Peltogyne catingae Ducke | species |
| Vegetation | Peltogyne maranhensis Huber ex Ducke | species |
| Vegetation | Peltophorum dubium (Spreng.) Taub. | species |
| Vegetation | Peperomia pellucida (L.) Kunth | species |
| Vegetation | Pera bicolor (Klotzsch) Müll.Arg. | species |
| Vegetation | Pera glabrata (Schott) Poepp. ex Baill. | species |
| Vegetation | Periandra coccinea (Schrad.) Benth. | species |
| Vegetation | Periandra mediterranea (Vell.) Taub. | species |
| Vegetation | Persea americana Mill. | species |
| Vegetation | Petrea volubilis L. | species |
| Vegetation | Pfaffia glomerata (Spreng.) Pedersen | species |
| Vegetation | Phaseolus lunatus L. | species |
| Vegetation | Phaseolus vulgaris L. | species |
| Vegetation | Phenakospermum guyannense (Rich.) Endl. ex Miq. | species |
| Vegetation | Philodendron acutatum Schott | species |
| Vegetation | Philodendron cordatum Kunth ex Schott | species |
| Vegetation | Philodendron imbe Schott ex Kunth. | species |
| Vegetation | Philonotis cernua (Wilson) Griffin & W.R.Buck | species |
| Vegetation | Philonotis uncinata (Schwägr.) Brid. | species |
| Vegetation | Phlebodium aureum (L.) J.Sm. | species |
| Vegetation | Phlebodium decumanum (Willd.) J.Sm. | species |
| Vegetation | Phoenix roebelenii O'Brien | species |
| Vegetation | Phoradendron quadrangulare (Kunth) Griseb. | species |
| Vegetation | Phyllanthus niruri L. | species |
| Vegetation | Phyllanthus orbiculatus Rich. | species |
| Vegetation | Phyllanthus tenellus Roxb. | species |
| Vegetation | Physalis angulata L. | species |
| Vegetation | Piper aduncum L. | species |
| Vegetation | Piper crassinervium Kunth | species |
| Vegetation | Piptadenia trisperma (Vell.) Benth. | species |
| Vegetation | Piriqueta duarteana (A. St.-Hil., A. Juss. & Cambess.) Urb. | species |
| Vegetation | Piriqueta hapala Arbo | species |
| Vegetation | Piriqueta viscosa Griseb. | species |
| Vegetation | Pistia stratiotes L. | species |
| Vegetation | Pithecellobium dulce (Roxb.) Benth. | species |
| Vegetation | Pityrogramma calomelanos (L.) Link | species |
| Vegetation | Plathymenia reticulata Benth. | species |
| Vegetation | Platonia insignis Mart. | species |
| Vegetation | Pleonotoma castelnaei (Bureau) Sandwith | species |
| Vegetation | Pleonotoma jasminifolia (Kunth) Miers | species |
| Vegetation | Pleonotoma orientalis Sandwith | species |
| Vegetation | Plumbago scandens L. | species |
| Vegetation | Plumeria pudica Jacq. | species |
| Vegetation | Plumeria rubra L. | species |
| Vegetation | Poecilanthe grandiflora Benth. | species |
| Vegetation | Pogonophora schomburgkiana Miers ex Benth. | species |
| Vegetation | Polygala glochidata Kunth | species |
| Vegetation | Polygala paniculata L. | species |
| Vegetation | Polygala trichosperma Jacq. | species |
| Vegetation | Pombalia calceolaria (L.) Paula-Souza | species |
| Vegetation | Pontederia cordata L. | species |
| Vegetation | Pontederia parviflora Alexander | species |
| Vegetation | Porophyllum ruderale (Jacq.) Cass. | species |
| Vegetation | Portulaca halimoides L. | species |
| Vegetation | Portulaca oleracea L. | species |
| Vegetation | Posoqueria acutifolia Mart. | species |
| Vegetation | Posoqueria latifolia (Rudge) Schult. | species |
| Vegetation | Pourouma guianensis Aubl. | species |
| Vegetation | Pouteria anomala (Pires) T.D.Penn. | species |
| Vegetation | Pouteria caimito (Ruiz & Pav.) Radlk. | species |
| Vegetation | Pouteria campanulata Baehni | species |
| Vegetation | Pouteria furcata T.D.Penn. | species |
| Vegetation | Pouteria glomerata (Miq.) Radlk. | species |
| Vegetation | Pouteria grandiflora (A.DC.) Baehni | species |
| Vegetation | Pouteria guianensis Aubl. | species |
| Vegetation | Pouteria krukovii (A.C.Sm.) Baehni | species |
| Vegetation | Pouteria laevigata (Mart.) Radlk. | species |
| Vegetation | Pouteria macrophylla (Lam.) Eyma | species |
| Vegetation | Pouteria manaosensis (Aubrév. & Pellegr.) T.D.Penn. | species |
| Vegetation | Pouteria multiflora (A.DC.) Eyma | species |
| Vegetation | Pouteria oblanceolata Pires | species |
| Vegetation | Pouteria opposita (Ducke) T.D.Penn. | species |
| Vegetation | Pouteria ramiflora (Mart.) Radlk. | species |
| Vegetation | Pouteria reticulata (Engl.) Eyma | species |
| Vegetation | Pouteria retinervis T.D.Penn. | species |
| Vegetation | Pouteria sagotiana (Baill.) Eyma | species |
| Vegetation | Pradosia surinamensis (Eyma) T.D.Penn. | species |
| Vegetation | Praxelis diffusa (Rich.) Pruski | species |
| Vegetation | Priva bahiensis A.DC. | species |
| Vegetation | Prosopis juliflora (Sw.) DC. | species |
| Vegetation | Protium altissimum (Aubl.) Marchand | species |
| Vegetation | Protium apiculatum Swart | species |
| Vegetation | Protium heptaphyllum (Aubl.) Marchand | species |
| Vegetation | Protium paniculatum Engl. | species |
| Vegetation | Protium spruceanum (Benth.) Engl. | species |
| Vegetation | Protium tenuifolium (Engl.) Engl. | species |
| Vegetation | Pseuderanthemum carruthersii (Seem.) Guillaumin | species |
| Vegetation | Pseudima frutescens (Aubl.) Radlk. | species |
| Vegetation | Pseudobombax grandiflorum (Cav.) A.Robyns | species |
| Vegetation | Psidium cattleyanum Sabine | species |
| Vegetation | Psidium firmum O.Berg | species |
| Vegetation | Psidium grandifolium Mart. ex DC. | species |
| Vegetation | Psidium guajava L. | species |
| Vegetation | Psidium kennedyanum Morong | species |
| Vegetation | Psidium cupreum O. Berg | species |
| Vegetation | Psidium guyanense Pers. | species |
| Vegetation | Psittacanthus dichroos (Mart.) Mart. | species |
| Vegetation | Psophocarpus tetragonolobus (L.) DC. | species |
| Vegetation | Psychotria carthagenensis Jacq. | species |
| Vegetation | Pteridium aquilinum (L.) Kuhn | species |
| Vegetation | Pteris tripartita Sw. | species |
| Vegetation | Pteris vittata L. | species |
| Vegetation | Pterocarpus officinalis Jacq. | species |
| Vegetation | Pterocarpus rohrii Vahl | species |
| Vegetation | Pterodon emarginatus Vogel | species |
| Vegetation | Pterolepis glomerata (Rottb.) Miq. | species |
| Vegetation | Pterolepis trichotoma (Rottb.) Cogn. | species |
| Vegetation | Qualea parviflora Mart. | species |
| Vegetation | Randia armata (Sw.) DC. | species |
| Vegetation | Rauvolfia ligustrina Willd. | species |
| Vegetation | Rhabdadenia biflora (Jacq.) Müll.Arg. | species |
| Vegetation | Rhipsalis baccifera (J.M.Muell.) Stearn | species |
| Vegetation | Rhizophora harrisonii Leechm. | species |
| Vegetation | Rhizophora mangle L. | species |
| Vegetation | Rhizophora racemosa G.Mey. | species |
| Vegetation | Rhynchosia minima (L.) DC. | species |
| Vegetation | Rhynchosia phaseoloides (Sw.) DC. | species |
| Vegetation | Rhynchospora cephalotes (L.) Vahl | species |
| Vegetation | Rhynchospora ciliata (Vahl) Kük. | species |
| Vegetation | Rhynchospora corymbosa (L.) Britton | species |
| Vegetation | Rhynchospora divaricata (Ham.) M.T.Strong | species |
| Vegetation | Rhynchospora hirsuta (Vahl) Vahl | species |
| Vegetation | Rhynchospora holoschoenoides (Rich.) Herter | species |
| Vegetation | Rhynchospora nervosa (Vahl) Boeckeler | species |
| Vegetation | Rhynchospora riparia (Nees) Boeckeler | species |
| Vegetation | Rhynchospora tenerrima Nees ex Spreng. | species |
| Vegetation | Richardia brasiliensis Gomes | species |
| Vegetation | Richardia grandiflora (Cham. & Schltdl.) Steud. | species |
| Vegetation | Richeria grandis Vahl | species |
| Vegetation | Ricinus communis L. | species |
| Vegetation | Rinorea guianensis Aubl. | species |
| Vegetation | Rinorea pubiflora (Benth.) Sprague & Sandwith | species |
| Vegetation | Rolandra fruticosa (L.) Kuntze | species |
| Vegetation | Rorippa nasturtium-aquaticum (L.) Hayek | species |
| Vegetation | Rosa spinosissima L. | species |
| Vegetation | Rosenbergiodendron formosum (Jacq.) Fagerl. | species |
| Vegetation | Rottboellia cochinchinensis (Lour.) Clayton | species |
| Vegetation | Roupala montana Aubl. | species |
| Vegetation | Rourea doniana Baker | species |
| Vegetation | Roystonea regia (Kunth) O.F.Cook | species |
| Vegetation | Rudgea cornifolia (Kunth) Standl. | species |
| Vegetation | Rudgea crassiloba (Benth.) B.L.Rob. | species |
| Vegetation | Rudgea jasminoides (Cham.) Müll.Arg. | species |
| Vegetation | Ruellia paniculata L. | species |
| Vegetation | Ruellia simplex C.Wright | species |
| Vegetation | Rugoloa pilosa (Sw.) Zuloaga | species |
| Vegetation | Rugoloa polygonata (Schrad.) Zuloaga | species |
| Vegetation | Ruprechtia laxiflora Meisn. | species |
| Vegetation | Russelia equisetiformis Schltdl. & Cham. | species |
| Vegetation | Sabicea cinerea Aubl. | species |
| Vegetation | Sacciolepis indica (L.) Chase | species |
| Vegetation | Sacciolepis vilvoides (Trin.) Chase | species |
| Vegetation | Sacoglottis guianensis Benth. | species |
| Vegetation | Sacoila lanceolata (Aubl.) Garay | species |
| Vegetation | Sagittaria guayanensis Kunth | species |
| Vegetation | Salvia splendens Sellow ex Nees | species |
| Vegetation | Salvinia auriculata Aubl. | species |
| Vegetation | Samanea inopinata (Harms) Barneby & J.W.Grimes | species |
| Vegetation | Samanea saman (Jacq.) Merr. | species |
| Vegetation | Samanea tubulosa (Benth.) Barneby & J.W.Grimes | species |
| Vegetation | Sapindus saponaria L. | species |
| Vegetation | Sapium glandulosum (L.) Morong | species |
| Vegetation | Sapium laurifolium (A.Rich.) Griseb. | species |
| Vegetation | Sapium marmieri Huber | species |
| Vegetation | Sarcoglottis amazonica Pabst | species |
| Vegetation | Sarcomphalus cinnamomum (Triana & Planch.)Hauenshild | species |
| Vegetation | Sauvagesia erecta L. | species |
| Vegetation | Schefflera umbrosa Frodin & Fiaschi | species |
| Vegetation | Schinus terebinthifolia Raddi | species |
| Vegetation | Schnella glabra (Jacq.) Dugand | species |
| Vegetation | Schnella outimouta (Aubl.) Wunderlin | species |
| Vegetation | Schnella platycalyx (Benth.) Wunderlin | species |
| Vegetation | Schnella rutilans (Spruce ex Benth.) Pittier | species |
| Vegetation | Schoepfia brasiliensis A.DC. | species |
| Vegetation | Schubertia grandiflora Mart. | species |
| Vegetation | Schultesia guianensis (Aubl.) Malme | species |
| Vegetation | Schwartzia brasiliensis (Choisy) Bedell ex Gir.-Cañas | species |
| Vegetation | Schwenckia americana Rooyen ex L. | species |
| Vegetation | Scleria bracteata Cav. | species |
| Vegetation | Scleria gaertneri Raddi | species |
| Vegetation | Scleria hirtella Sw. | species |
| Vegetation | Scleria latifolia Sw. | species |
| Vegetation | Scoparia dulcis L. | species |
| Vegetation | Secondatia densiflora A.DC. | species |
| Vegetation | Securidaca bialata Benth. | species |
| Vegetation | Securidaca divaricata Nees & Mart. | species |
| Vegetation | Securidaca diversifolia (L.) S.F.Blake | species |
| Vegetation | Selenicereus setaceus (Salm-Dyck) A. Berger ex Werderm. | species |
| Vegetation | Senegalia bonariensis (Gillies ex Hook. & Arn.) Seigler & Ebinger | species |
| Vegetation | Senegalia polyphylla (DC.) Britton & Rose | species |
| Vegetation | Senna aculeata (Pohl ex Benth.) H.S.Irwin & Barneby | species |
| Vegetation | Senna alata (L.) Roxb. | species |
| Vegetation | Senna alexandrina Mill. | species |
| Vegetation | Senna cernua (Balb.) H.S.Irwin & Barneby | species |
| Vegetation | Senna corymbosa (Lam.) H.S.Irwin & Barneby | species |
| Vegetation | Senna fruticosa (Mill.) H.S.Irwin & Barneby | species |
| Vegetation | Senna georgica H.S.Irwin & Barneby | species |
| Vegetation | Senna hirsuta (L.) H.S.Irwin & Barneby | species |
| Vegetation | Senna latifolia (G.Mey.) H.S.Irwin & Barneby | species |
| Vegetation | Senna macranthera (DC. ex Collad.) H.S.Irwin & Barneby | species |
| Vegetation | Senna multijuga (Rich.) H.S.Irwin & Barneby | species |
| Vegetation | Senna obtusifolia (L.) H.S.Irwin & Barneby | species |
| Vegetation | Senna occidentalis (L.) Link | species |
| Vegetation | Senna polyphylla (Jacq.) H.S.Irwin & Barneby | species |
| Vegetation | Senna siamea (Lam.) H.S.Irwin & Barneby | species |
| Vegetation | Senna silvestris (Vell.) H.S.Irwin & Barneby | species |
| Vegetation | Senna undulata (Benth.) H.S.Irwin & Barneby | species |
| Vegetation | Serenoa repens (W. Bartram) Small | species |
| Vegetation | Serjania paucidentata DC. | species |
| Vegetation | Serjania salzmanniana Schltdl. | species |
| Vegetation | Sesbania exasperata Kunth | species |
| Vegetation | Sesuvium portulacastrum (L.) L. | species |
| Vegetation | Setaria parviflora (Poir.) Kerguélen | species |
| Vegetation | Setaria tenax (Rich.) Desv. | species |
| Vegetation | Setaria vulpiseta (Lam.) Roem. & Schult. | species |
| Vegetation | Sida acuta Burm.f. | species |
| Vegetation | Sida ciliaris L. | species |
| Vegetation | Sida cordifolia L. | species |
| Vegetation | Sida glaziovii K.Schum. | species |
| Vegetation | Sida rhombifolia L. | species |
| Vegetation | Sidastrum micranthum (A.St.-Hil.) Fryxell | species |
| Vegetation | Sigesbeckia orientalis L. | species |
| Vegetation | Simaba guianensis Aubl. | species |
| Vegetation | Simarouba amara Aubl. | species |
| Vegetation | Simarouba versicolor A.St.-Hil. | species |
| Vegetation | Siparuna guianensis Aubl. | species |
| Vegetation | Sloanea grandiflora Sm. | species |
| Vegetation | Sloanea synandra Spruce ex Benth. | species |
| Vegetation | Smilax japicanga Griseb. | species |
| Vegetation | Smilax syphilitica Humb. & Bonpl. ex Willd. | species |
| Vegetation | Solanum aculeatissimum Jacq. | species |
| Vegetation | Solanum americanum Mill. | species |
| Vegetation | Solanum apiculatum Sendtn. | species |
| Vegetation | Solanum asperolanatum Ruiz & Pav. | species |
| Vegetation | Solanum asperum Rich. | species |
| Vegetation | Solanum caavurana Vell. | species |
| Vegetation | Solanum crinitum Lam. | species |
| Vegetation | Solanum erianthum D.Don | species |
| Vegetation | Solanum grandiflorum Ruiz & Pav. | species |
| Vegetation | Solanum jamaicense Mill. | species |
| Vegetation | Solanum lycocarpum A.St.-Hil. | species |
| Vegetation | Solanum palinacanthum Dunal | species |
| Vegetation | Solanum paludosum Moric. | species |
| Vegetation | Solanum paniculatum L. | species |
| Vegetation | Solanum stramoniifolium Jacq. | species |
| Vegetation | Solanum subinerme Jacq. | species |
| Vegetation | Solidago chilensis Meyen | species |
| Vegetation | Sonchus oleraceus L. | species |
| Vegetation | Sorghum halepense (L.) Pers. | species |
| Vegetation | Sparattosperma leucanthum (Vell.) K. Schum. | species |
| Vegetation | Spartina alterniflora Loisel. | species |
| Vegetation | Spathiphyllum gardneri Schott | species |
| Vegetation | Spathodea campanulata P. Beauv. | species |
| Vegetation | Sphagneticola brachycarpa (Baker) Pruski | species |
| Vegetation | Sphagneticola trilobata (L.) Pruski | species |
| Vegetation | Spigelia anthelmia L. | species |
| Vegetation | Spondias mombin L. | species |
| Vegetation | Spondias venulosa (Mart. ex Engl.) Engl. | species |
| Vegetation | Sporobolus indicus (L.) R. Br. | species |
| Vegetation | Sporobolus virginicus (L.) Kunth | species |
| Vegetation | Stachytarpheta cayennensis (Rich.) Vahl | species |
| Vegetation | Staelia virgata (Link ex Roem. & Schult.) K.Schum. | species |
| Vegetation | Stemodia foliosa Benth. | species |
| Vegetation | Sterculia apetala (Jacq.) H.Karst. | species |
| Vegetation | Sterculia pruriens (Aubl.) K.Schum. | species |
| Vegetation | Sterculia striata A.St.-Hil. & Naudin | species |
| Vegetation | Stigmaphyllon bannisterioides (L.) C.E.Anderson | species |
| Vegetation | Streptostachys asperifolia Desv. | species |
| Vegetation | Stryphnodendron adstringens (Mart.) Coville | species |
| Vegetation | Stryphnodendron guianense (Aubl.) Benth. | species |
| Vegetation | Stryphnodendron platyspicum Rizzini & Heringer | species |
| Vegetation | Stryphnodendron polyphyllum Mart. | species |
| Vegetation | Stryphnodendron racemiferum (Ducke) W.A.Rodrigues | species |
| Vegetation | Stryphnodendron polyphyllum Mart. | species |
| Vegetation | Stryphnodendron pulcherrimum (Willd.) Hochr. | species |
| Vegetation | Stylosanthes angustifolia Vogel | species |
| Vegetation | Stylosanthes guianensis (Aubl.) Sw. | species |
| Vegetation | Stylosanthes humilis Kunth | species |
| Vegetation | Stylosanthes scabra Vogel | species |
| Vegetation | Swartzia flaemingii Raddi | species |
| Vegetation | Swartzia oblata R.S.Cowan | species |
| Vegetation | Swartzia psilonema Harms | species |
| Vegetation | Swartzia recurva Poepp. | species |
| Vegetation | Swartzia schomburgkii Benth. | species |
| Vegetation | Syagrus cocoides Mart. | species |
| Vegetation | Syagrus oleracea (Mart.) Becc. | species |
| Vegetation | Symphonia globulifera L.f. | species |
| Vegetation | Symphyotrichum squamatum (Spreng.) G.L.Nesom | species |
| Vegetation | Synedrella nodiflora (L.) Gaertn. | species |
| Vegetation | Syngonanthus cuyabensis (Bong.) Giul., Hensold & L.R. Parra | species |
| Vegetation | Syngonanthus gracilis (Bong.) Ruhland | species |
| Vegetation | Syzygium cumini (L.) Skeels | species |
| Vegetation | Syzygium jambos (L.) Alston | species |
| Vegetation | Syzygium malaccense (L.) Merr. & L.M. Perry | species |
| Vegetation | Tabebuia aurea (Silva Manso) Benth. & Hook.f. ex S.Moore | species |
| Vegetation | Tabebuia roseoalba (Ridl.) Sandwith | species |
| Vegetation | Tabernaemontana angulata Mart. ex Müll. Arg. | species |
| Vegetation | Tabernaemontana flavicans Willd. ex Roem. & Schult. | species |
| Vegetation | Tabernaemontana laeta Mart. | species |
| Vegetation | Tabernaemontana macrocalyx Müll. Arg. | species |
| Vegetation | Taccarum ulei Engl. & K.Krause | species |
| Vegetation | Taccarum weddellianum Brongn. ex Schott | species |
| Vegetation | Tachigali aurea Tul. | species |
| Vegetation | Tachigali setifera (Ducke) Zarucchi & Herend. | species |
| Vegetation | Tachigali subvelutina (Benth.) Oliveira-Filho | species |
| Vegetation | Tachigali vulgaris L.G.Silva & H.C.Lima | species |
| Vegetation | Tagetes erecta L. | species |
| Vegetation | Tagetes minuta L. | species |
| Vegetation | Tagetes patula L. | species |
| Vegetation | Talinum fruticosum (L.) Juss. | species |
| Vegetation | Talinum paniculatum (Jacq.) Gaertn. | species |
| Vegetation | Talisia cupularis Radlk. | species |
| Vegetation | Talisia esculenta (A.St.-Hil.) Radlk. | species |
| Vegetation | Talisia guianensis Aubl. | species |
| Vegetation | Talisia mollis Kunth ex Cambess. | species |
| Vegetation | Talisia retusa Cowan | species |
| Vegetation | Talisia subalbens (Mart.) Radlk. | species |
| Vegetation | Tamarindus indica L. | species |
| Vegetation | Tanacetum parthenium (L.) Sch.Bip. | species |
| Vegetation | Tanaecium decorticans Frazão & L.G. Lohmann | species |
| Vegetation | Tapirira guianensis Aubl. | species |
| Vegetation | Tapirira obtusa (Benth.) J.D.Mitch. | species |
| Vegetation | Tarenaya aculeata (L.) Soares Neto & Roalson | species |
| Vegetation | Tarenaya spinosa (Jacq.) Raf. | species |
| Vegetation | Tassadia propinqua Decne. | species |
| Vegetation | Tecoma stans (L.) Juss. ex Kunth | species |
| Vegetation | Tectona grandis L.f. | species |
| Vegetation | Telmatoblechnum serrulatum (Rich.) Perrie, D.J. Ohlsen & Brownsey | species |
| Vegetation | Terminalia amazonia (J.F.Gmel.) Exell | species |
| Vegetation | Terminalia argentea Mart. & Zucc. | species |
| Vegetation | Terminalia catappa L. | species |
| Vegetation | Terminalia dichotoma G.Mey. | species |
| Vegetation | Terminalia glabrescens Mart. | species |
| Vegetation | Terminalia grandis (Ducke) Gere & Boatwr. | species |
| Vegetation | Terminalia guyanensis Eichler | species |
| Vegetation | Terminalia lucida Hoffmanns. ex Mart. & Zucc. | species |
| Vegetation | Terminalia macrophylla (Spruce ex Eichler) Gere & Boatwr. | species |
| Vegetation | Tetracera breyniana Schltdl. | species |
| Vegetation | Tetracera willdenowiana Steud. | species |
| Vegetation | Tetrapterys discolor (G.Mey.) DC. | species |
| Vegetation | Tetrapterys maranhamensis A.Juss. | species |
| Vegetation | Tetraulacium veroniciforme Turcz. | species |
| Vegetation | Thalia geniculata L. | species |
| Vegetation | Theobroma grandiflorum (Willd. ex Spreng.) K.Schum. | species |
| Vegetation | Theobroma speciosum Willd. ex Spreng. | species |
| Vegetation | Thevetia peruviana (Pers.) K.Schum. | species |
| Vegetation | Thunbergia erecta (Benth.) T. Anderson | species |
| Vegetation | Thyrsodium spruceanum Benth. | species |
| Vegetation | Tilesia baccata (L.) Pruski | species |
| Vegetation | Tillandsia paraensis Mez | species |
| Vegetation | Tithonia diversifolia (Hemsl.) A.Gray | species |
| Vegetation | Tococa guianensis Aubl. | species |
| Vegetation | Tocoyena brasiliensis Mart. | species |
| Vegetation | Tocoyena formosa (Cham. & Schltdl.) K.Schum. | species |
| Vegetation | Tocoyena sellowiana (Cham. & Schltdl.) K.Schum. | species |
| Vegetation | Tovomita choisyana Planch. & Triana | species |
| Vegetation | Tradescantia pallida (Rose) D.R.Hunt | species |
| Vegetation | Trattinnickia rhoifolia Willd. | species |
| Vegetation | Trema micrantha (L.) Blume | species |
| Vegetation | Trichilia cipo (A.Juss.) C.DC. | species |
| Vegetation | Trichilia micrantha Benth. | species |
| Vegetation | Trichospira verticillata (L.) S.F.Blake | species |
| Vegetation | Tridax procumbens L. | species |
| Vegetation | Triumfetta semitriloba Jacq. | species |
| Vegetation | Turnera melochioides Cambess. | species |
| Vegetation | Turnera pumilea L. | species |
| Vegetation | Turnera scabra Millsp. | species |
| Vegetation | Turnera subulata Sm. | species |
| Vegetation | Turnera ulmifolia L. | species |
| Vegetation | Unonopsis guatterioides (A.DC.) R.E.Fr. | species |
| Vegetation | Urena lobata L. | species |
| Vegetation | Urochloa brizantha (Hochst. ex A.Rich.) R.D.Webster | species |
| Vegetation | Urochloa decumbens (Stapf) R.D.Webster | species |
| Vegetation | Urochloa fusca (Sw.) B.F.Hansen & Wunderlin | species |
| Vegetation | Urochloa humidicola (Rendle) Morrone & Zuloaga | species |
| Vegetation | Urochloa mutica (Forssk.) T.Q.Nguyen | species |
| Vegetation | Urochloa plantaginea (Link) R.D.Webster | species |
| Vegetation | Urochloa platyphylla (Munro ex C.Wright) R.D.Webster | species |
| Vegetation | Urtica dioica L. | species |
| Vegetation | Utricularia fimbriata Kunth | species |
| Vegetation | Utricularia foliosa L. | species |
| Vegetation | Utricularia nigrescens Sylvén | species |
| Vegetation | Utricularia simulans Pilg. | species |
| Vegetation | Vachellia farnesiana (L.) Wight & Arn. | species |
| Vegetation | Vanilla palmarum (Salzm. ex Lindl.) Lindl. | species |
| Vegetation | Vantanea guianensis Aubl. | species |
| Vegetation | Varronia curassavica Jacq. | species |
| Vegetation | Varronia globosa Jacq. | species |
| Vegetation | Varronia multispicata (Cham.) Borhidi | species |
| Vegetation | Vasconcellea microcarpa (Jacq.) A.DC. | species |
| Vegetation | Vatairea erythrocarpa (Ducke) Ducke | species |
| Vegetation | Vataireopsis speciosa Ducke | species |
| Vegetation | Vernicia fordii (Hemsl.) Airy Shaw | species |
| Vegetation | Vernonanthura brasiliana (L.) H.Rob. | species |
| Vegetation | Vernonanthura chamaedrys (Less.) H.Rob. | species |
| Vegetation | Vernonanthura polyanthes (Sprengel) Vega & Dematteis | species |
| Vegetation | Vicia faba L. | species |
| Vegetation | Vigna lasiocarpa (Mart. ex Benth.) Verdc. | species |
| Vegetation | Vigna longifolia (Benth.) Verdc. | species |
| Vegetation | Vigna luteola (Jacq.) Benth. | species |
| Vegetation | Vigna unguiculata (L.) Walp. | species |
| Vegetation | Viola odorata L. | species |
| Vegetation | Virola calophylla Warb. | species |
| Vegetation | Virola sebifera Aubl. | species |
| Vegetation | Virola surinamensis (Rol. ex Rottb.) Warb. | species |
| Vegetation | Virola venosa (Benth.) Warb. | species |
| Vegetation | Vismia brasiliensis Choisy | species |
| Vegetation | Vismia guianensis (Aubl.) Choisy | species |
| Vegetation | Vismia japurensis Reichardt | species |
| Vegetation | Vismia sandwithii Ewan | species |
| Vegetation | Vitex cymosa Bertero ex Spreng. | species |
| Vegetation | Vitex polygama Cham. | species |
| Vegetation | Vitex schomburgkiana Schauer | species |
| Vegetation | Vittaria lineata (L.) Sm. | species |
| Vegetation | Waltheria indica L. | species |
| Vegetation | Waltheria viscosissima A.St.-Hil. | species |
| Vegetation | Wedelia goyazensis Gardner | species |
| Vegetation | Wedelia rudis (Baker) H.Rob. | species |
| Vegetation | Ximenia americana L. | species |
| Vegetation | Xylopia aromatica (Lam.) Mart. | species |
| Vegetation | Xylopia brasiliensis Spreng. | species |
| Vegetation | Xylopia emarginata Mart. | species |
| Vegetation | Xylopia frutescens Aubl. | species |
| Vegetation | Xylopia nitida Dunal | species |
| Vegetation | Xylopia parviflora Spruce | species |
| Vegetation | Xylopia sericea A.St.-Hil. | species |
| Vegetation | Xyris anceps Lam. | species |
| Vegetation | Xyris jupicai Rich. | species |
| Vegetation | Xyris macrocephala Vahl | species |
| Vegetation | Zanthoxylum acuminatum (Sw.) Sw. | species |
| Vegetation | Zanthoxylum monogynum A.St.-Hil. | species |
| Vegetation | Zanthoxylum rhoifolium Lam. | species |
| Vegetation | Zea mays L. | species |
| Vegetation | Zinnia elegans Jacq. | species |
| Vegetation | Zollernia paraensis Huber | species |
| Vegetation | Zornia curvata Mohlenbr. | species |
| Vegetation | Zornia diphylla (L.) Pers. | species |
| Vegetation | Zornia guanipensis Pittier | species |
| Vegetation | Zornia latifolia Sm. | species |
| Vegetation | Zornia reticulata Sm. | species |
| Vegetation | Bambusoideae Luerss. | subfamily |
| Vegetation | Ficus americana subsp. guianensis (Desv.) C.C.Berg | subspecies |
| Vegetation | Inga sertulifera DC. subsp. sertulifera | subspecies |
| Vegetation | Inga vera subsp. affinis (DC.) T.D.Penn. | subspecies |
| Vegetation | Ipomoea carnea subsp. fistulosa (Mart. ex Choisy) D.F.Austin | subspecies |
| Vegetation | Ipomoea pes-caprae subsp. brasiliensis (L.) Ooststr. | subspecies |
| Vegetation | Porophyllum ruderale (Jacq.) Cass. subsp. ruderale | subspecies |
| Vegetation | Senna silvestris (Vell.) H.S.Irwin & Barneby subsp. silvestris | subspecies |
| Vegetation | Abarema cochleata (Willd.) Barneby & J.W.Grimes var. cochleata | variety |
| Vegetation | Abarema jupunba (Willd.) Britton & Killip var. jupunba | variety |
| Vegetation | Asparagus densiflorus var. sprengeri Kunth | variety |
| Vegetation | Bactris acanthocarpa var. exscapa Barb.Rodr. | variety |
| Vegetation | Bactris gasipaes var. chichagui (H.Karst.) A.J.Hend. | variety |
| Vegetation | Boerhaavia diffusa var. hirsuta Kuntze | variety |
| Vegetation | Chamaecrista ensiformis var. maranonica (H.S.Irwin) H.S.Irwin & Barneby | variety |
| Vegetation | Clitoria falcata Lam. var. falcata | variety |
| Vegetation | Ipomoea squamosa Choisy var. squamosa | variety |
| Vegetation | Ixora coccinea var. lutea (Hutch.) Corner | variety |
| Vegetation | Mimosa pudica var. tetrandra (Humb. & Bonpl. ex Willd.) DC. | variety |
| Vegetation | Philodendron hederaceum (Jacq.) Schott var. hederaceum | variety |
| Vegetation | Platymiscium trinitatis var. duckei (Huber) Klitg. | variety |
| Vegetation | Stylosanthes guianensis var. pauciflora M.B.Ferreira & Sousa Costa | variety |
| Vegetation | Zygia latifolia var. communis Barneby & J.W.Grimes | variety |
